# Why the Same Dose of Botulinum Toxin A Is Less Predictable in Small Muscles: A Discrete Threshold Model

**DOI:** 10.3390/toxins18090403

**Published:** 2026-09-21

**Authors:** Andrea Felice Armenti, Francesco Armenti

**Affiliations:** 1 LA VISION Training Institute, Via Magenta, 5, 00185 Rome, Italy; 2 Independent Researcher, 00185 Rome, Italy

**Keywords:** botulinum toxin, dose–response, threshold models, finite-size scaling, identifiability, neuromuscular junction, pharmacodynamics

## Abstract

Pharmacodynamic models of botulinum toxin type A map a mean concentration onto a smooth dose–response curve, yet the outcome is binary and arises from a finite population of near-threshold motor units. We analyse a discrete model in which *N* units are silenced once local SNAP-25 cleavage exceeds a unit-specific threshold, and a functional block of neuromuscular transmission follows once the silenced fraction exceeds a collective threshold. Whether that collective threshold is a fraction of the population or a fixed count changes the location of the dose–response curve and not its steepness: under a fraction, potency is invariant to unit number at fixed dose–concentration gain, and the transition contracts in absolute dose where fixed-count architectures require it to widen. The width contracts as $N^{-1/2}$, an exponent that is prior and not claimed here. One consequence needs no measurement: a quantal terminal response caps the silenced fraction below unity, so any fixed count fails above a target size it determines, whereas the toxin block targets differ by orders of magnitude. A second reinterprets existing data, flat cohort dose–response curves being what a near-step individual response predicts once convolved with between-subject dispersion. Outcome is accordingly less repeatable in the smallest targets. No parameter is fitted.

## 1. Introduction

### 1.1. Molecular Background

Botulinum toxin type A (BoNT-A) acts through a specific molecular cascade that disrupts vesicular neurotransmitter release at cholinergic nerve terminals [1,2,3,4]. High-affinity binding to SV2 with ganglioside co-receptors is followed by receptor-mediated endocytosis, conformational change in the acidic endosome, and translocation of the light chain to the cytosol [2,5,6,7,8]. There, the toxin cleaves SNAP-25 at the Gln197–Arg198 bond [5], disrupting the SNARE complexes required for calcium-dependent vesicle fusion [9,10,11].

SNAP-25 cleavage kinetics follow a characteristic temporal pattern, with initial enzymatic activity occurring within hours of toxin application, progressing to near-complete substrate depletion over 24–48 h [1]. The suppression of synaptic vesicle release is strongly linked to SNAP-25 cleavage, but the functional relationship is not represented here as a fully resolved molecular map. In the present framework, cleavage is treated as an effective intermediate variable summarising a broader cascade that ultimately reduces acetylcholine release.

### 1.2. Limitations of Continuous Mean-Field Models

Current pharmacokinetic and pharmacodynamic models for botulinum toxin predominantly employ continuous population-level approaches that aggregate individual terminal responses into smooth dose-response curves. These models typically assume Hill-type or logistic dose–response relationships [12,13], where the probability of unit silencing increases monotonically with local toxin concentration according to a sigmoidal function. In that formulation, the sigmoidicity exponent *n* is a shape parameter fitted to the data and carrying no mechanistic commitment [13]; Section 3.7 shows that the present architecture instead fixes it, as $n_{\mathrm{eff}}\propto \sqrt{N}$. Such formulations treat local concentration as a sufficient statistic for terminal behaviour, neglecting the discreteness of binding events and the threshold-dependent nature of individual terminal responses [14,15], the spatial heterogeneity of toxin distribution [16], and the transition from single-terminal behaviour to collective dynamics [17,18,19] (Supplementary Materials S2.4).

### 1.3. An Observation That Motivates a Threshold Account

One empirical pattern in the BoNT-A literature is difficult to accommodate within a continuous dose–effect description, and it motivates what follows. Cleavage of only 10–15% of total SNAP-25 is sufficient to abolish neurotransmission at the frog neuromuscular junction and in rodent spinal cord cultures [20,21], with as little as 2–3% sufficient to silence miniature postsynaptic currents [22]; and across such preparations the measured amount of cleaved SNAP-25 correlates poorly with the extent of neuroparalysis [1].

For BoNT-E, by contrast, cleavage and inhibition of exocytosis track one another closely [1]. The absence of correlation is the signature of a threshold: if outcome is binary in a collective variable, an assay that varies smoothly across the transition appears uncorrelated with an outcome that steps (Section 2.5, and Supplementary Materials S1.2).

The two serotypes cleave the same substrate but leave different products, and the difference is structural rather than quantitative. BoNT-A cuts at Gln197–Arg198, removing the last nine residues of SNAP-25; BoNT-E cuts at Arg180–Ile181, removing twenty-six [3]. The shorter truncation is still incorporated into the SNARE complex, which it renders non-functional, whereas the longer one is not [23]. The two toxins therefore inhibit transmission by different molecular routes—a poisoned complex against an absent one—and any account of the serotype asymmetry has to accommodate that rather than assume the two are the same reaction at different rates.

### 1.4. Objectives

One claim in this paper is new, and we state it here so that a reader need not look for it: the collective threshold that governs functional block is a fraction of the unit population and not a fixed count, which makes potency invariant to target size and predictability dependent on it. Everything else below is either prior, or an observation, or a condition on testing that claim.

We analyse a discrete model in which each unit is silenced once local cleavage exceeds a unit-specific threshold, and a functional block of neuromuscular transmission follows once the silenced fraction exceeds a collective threshold. Our aim is to identify which macroscopic regularities require this architecture, and to express them in quantities that can be measured. The comparison class is a stochastic mean-only model $Y∼\mathrm{Bernoulli}(g\left(\bar{C}\right))$ rather than the deterministic mapping that such comparisons usually adopt (Section 3.1), and two properties of the discrete model do not survive it and are recorded as observations rather than results.

Two properties of the discrete model survive that benchmark, and the analysis rests on them; neither is claimed as new in itself, and Section 3.11 states the perimeter of what is:

(i) A sharpness law linking transition width, independent-unit number, and threshold dispersion. The width of the population dose–response transition scales as $N^{-1/2}$ with a prefactor set by $\Theta_{\mathrm{sys}}$ and by the slope of the terminal silencing probability at the critical concentration—and that slope is controlled by the dispersion of unit thresholds. A mean-only mapping contains no *N*, so it cannot express this relation and cannot be transported between injection targets of different size. The law is exact in the central-limit regime, testable by comparing dose–response transitions across targets, and it converts $\sigma_{\theta }$ from an unobservable microscopic parameter into an estimable one.
(ii) Geometry non-sufficiency at matched mean dose. Two spatial concentration fields with identical spatial mean produce different block probabilities. The field family is a Gaussian diffusion kernel and not a point mass, for the reason given in Section 2.9, and its summary is a critical dispersion radius $s^{*}$ that maps onto injection volume and dilution.

The claim stated at the head of this section is the one this paper advances on its own account, and Section 3.8 makes it quantitative. Fixed-count architectures require the opposite in both—potency and width—and Section 3.2 rules them out above a target size that the silenceability ceiling fixes at a few hundred to a few thousand units, without any new measurement.

We also examine two dynamical extensions (progressive threshold drift and reduced effective uptake) as illustrations of how the same architecture behaves under repeated exposure, and we are explicit that these are phenomenological compressions rather than mechanistic derivations.

### 1.5. Scope, and How to Read the Results

This is a theoretical paper. No parameter is estimated by fitting the model to data; each is derived, declared or constrained by inverting a published measurement, and results are of four kinds distinguished in Table 1. What the central relation does not require is worth stating at the outset, since the list is short and unusual: no calibration of local concentration, no single-terminal measurement, no knowledge of lot potency or of the terminal pool fraction, and no absolute dose scale; responder status against the administered dose, in targets whose independent-unit count differs, suffices.

Four results follow, and they differ in what they demand. Two require no new measurement of any kind and are the ones a reader can act on today. Because the quantal terminal response caps the attainable silenced fraction below unity, a collective threshold set at a fixed count implies a target size above which block cannot occur, and the range over which botulinum toxin works excludes every such count (Section 3.2). And the flatness of published cohort dose–response curves, read ordinarily as evidence of a graded mechanism, is what a near-step individual response looks like once convolved with between-subject dispersion in dose requirement—a reinterpretation of data already collected, which the same relation makes exclusive (Section 4.8). The third result requires a measurement nobody has made, but that is not difficult: the fraction of neuronal SNAP-25 in the releasable terminal pool (Section 2.5). The fourth requires a dose–response experiment of a shape that has not been run: potency invariant to target size, against the fixed-count alternative in which it moves (Section 3.8, with the design in Section 4.8).

Three limitations govern the whole of what follows and are stated here rather than deferred: *N* must be instantiated as an effective independent-unit count and not an anatomical one, because sibling terminals are correlated (Section 6); all absolute concentrations depend on an unmeasured pool fraction (Section 2.5); and the model has no time axis, so nothing here describes onset, duration or recovery (Section 2.10).

Three tables carry the epistemic bookkeeping, and they answer different questions. Table 1 classifies results by how they were obtained; Table 2 states which unmeasured quantity each reported number depends on, and by how much; Supplementary Table S8 states what the paper does not claim. A hedged formulation anywhere in this paper is a pointer to the second table unless the third says otherwise. A model of this kind should be readable by someone who later measures one of those quantities, which requires every dependence to be a stated map rather than a buried choice.

## 2. The Discrete Threshold Model

We model the target muscle as a population of *N* discrete units. A unit is the smallest element that fails independently; anatomically, it is a nerve terminal, but terminals belonging to one motor unit share the neuronal determinants of their threshold, so the count that enters every result below is the number of independent units and not the anatomical terminal count (Section 6, Supplementary Materials S1). We write *N* throughout and state where the distinction matters.

The construction is deliberately reduced: the mapping from uptake to SNAP-25 cleavage, and from cleavage to terminal silencing, is a phenomenological compression of a multi-step cascade (receptor binding, endocytosis, translocation, intracellular trafficking, SNARE turnover, vesicle-cycle recovery), not a resolved mechanistic reconstruction. Complete derivations and assumptions are in Supplementary Materials S1.

### 2.1. Terminal State Variable

Each terminal i=1,…,N carries a binary state

(1) $$S_{i}\in \left\{0,\;1\right\},$$

with $S_{i}=1$ denoting a functionally silenced terminal. Section 4.3 replaces this binary indicator with a graded response and identifies the conditions under which the binary idealisation is adequate.

### 2.2. Terminal Uptake and Cleavage

Toxin entry into terminal *i* is represented by a count of productive translocation events—light-chain molecules that reach the cytosol in catalytically competent form—modelled as

(2) $$K_{i}∼\mathrm{Poisson}\left(\lambda C_{i}\right),$$

where $C_{i}$ is the local extracellular concentration in reference units and λ is the expected number of productive events per terminal per unit concentration. Each event contributes multiplicatively to substrate depletion, so the cleaved SNAP-25 fraction is

(3) $$f_{i}=1-e^{-\eta K_{i}},$$

with η being the per-event cleavage yield, absorbing the post-internalisation reaction time. The count scale λ and the per-event yield η are carried separately rather than as their product κ:=λη, because collapsing them places the expected count below one event per unit and the model degenerates into a Poisson entry gate (Supplementary Materials S1.4).

We therefore treat λ as an explicit parameter with a stated biological meaning and adopt λ=35 as the reference, giving 14.3 productive events per unit at ${\bar{C}}_{\mathrm{crit}}$. The value is declared and not measured; Section 4.6 and Supplementary Table S18 report the sweep λ∈[1,500] and the λ-dependence of every conclusion.

### 2.3. Terminal Threshold

Terminal *i* is silenced when the cleaved fraction reaches its threshold:

(4) $$S_{i}=I\left(f_{i}\geq \theta_{i}\right).$$

Because $f_{i}$ is a fraction, $\theta_{i}$ must be supported on [0,1]: a threshold above unity would describe a terminal requiring more than complete cleavage; hence, one that cannot be silenced at any dose. We therefore take

(5) $$\theta_{i}∼\mathrm{Beta}\left(a,b\right),\;a=\mu_{\theta }\nu ,\;b=\left(1-\mu_{\theta }\right)\nu ,\;\nu =\frac{\mu_{\theta }\left(1-\mu_{\theta }\right)}{\sigma_{\theta }^{2}}-1,$$

which realises any admissible mean and variance on the unit interval; at the human reference, this is Beta(21.72,21.72). An unbounded surrogate such as a log-normal law assigns positive mass above unity and introduces a spurious ceiling on the attainable silenced fraction, distinct from the quantal ceiling of Section 3.2, which is a property of the mechanism and is retained (Supplementary Materials S1.13).

The mechanistic case for a sharp rather than graded terminal response rests on the dominant-negative behaviour of the cleavage product: SNAP-25_197_ is retained in the SNARE complex and inhibits exocytosis beyond the simple loss of intact SNAP-25, so effective release probability falls faster than the cleaved fraction rises [23], and cleavage and functional blockade are correspondingly non-proportional in cultured neurons [24]. A binary indicator is the limiting case of that steepening; Section 4.3 quantifies how steep the cleavage-to-release map must be for the idealisation to hold.

### 2.4. Mechanistic Grounding of the Threshold

Quantal transmission theory constrains $\theta_{i}$ without determining it, and the construction is set out in Supplementary Materials S1.13. Endplate potential amplitude is proportional to the quantal content *m*, the number of vesicles released per nerve impulse [25]; transmission is secured by a safety factor SF, the ratio of endplate potential amplitude to the action-potential threshold [26], lying in the range 3–5 in normal adult mammals [27]; and transmission fails when *m* falls below $m_{0}/\mathrm{SF}$.

### 2.5. Relating the Model to Cleavage Assays

The quantity $f_{i}$ in Equations (2) and (3) is the cleaved fraction of the SNAP-25 pool that participates in release at unit *i*. Published cleavage measurements are not that quantity. They are densitometric estimates from immunoblots of whole-tissue or whole-culture lysates, in which the denominator is all neuronal SNAP-25 present in the preparation—terminal, preterminal, axonal and somatic—and the numerator is summed over all units, intoxicated or not. Writing ρ for the fraction of neuronal SNAP-25 residing in the releasable terminal pool, the assay reports

(6) measuredcleavage=ρ〈f〉,

where 〈f〉 is the mean over units of the model’s $f_{i}$. The distinction is not interpretive licence; it follows from what is in the lysate.

Equation (6) converts the empirical figure into a constraint that the model must satisfy rather than a value it may assume. At ${\bar{C}}_{\mathrm{crit}}$, the model gives 〈f〉=0.549, so reproducing a measured 12.5% requires ρ≃0.23. A second anchor, mammalian and in vivo, is available: in rat gastrocnemius, paralysis was probable once the ratio of cleaved to intact SNAP-25 exceeded 0.35 [28], that is a cleaved fraction of 0.259, which gives ρ≃0.47. That the two quantities separate at all is independently visible in the mouse hemidiaphragm, where SNAP-25 immunoreactivity was still largely intact one hour after paralysis and largely lost three hours later [29]. The model therefore predicts

(7) ρ∈[0.23,0.47],

a factor of two, set by which preparation the anchor is taken from and not by any freedom in the model. This is a prediction and not an added degree of freedom: ρ is in principle measurable by subcellular fractionation, and a measured value near unity would refute the present parameterisation outright. A second reading, in which ρ≈1 and the small measured fractions are carried by a strong dominant-negative term instead, leaves the sharpness law intact and rescales only its constants; both readings, and the species-specific safety factor that changes every constant and no exponent, are set out in Supplementary Materials S1.16–S1.17.

The two readings make opposite predictions for ρ, so the question is empirical rather than a matter of preference. We adopt the first for the numerical work, and the structural results do not depend on which is correct.

### 2.6. System-Level Integration

The silenced fraction and the clinical readout are

(8) $$\Phi =\frac{1}{N}\sum_{i=1}^{N}S_{i},\;Y=I\left(\Phi \geq \Theta_{\mathrm{sys}}\right).$$

The collective threshold $\Theta_{\mathrm{sys}}$ is the fraction of units that must be silenced before the clinical readout changes. It is not the safety factor of neuromuscular transmission, which enters one level below through the unit threshold $\theta_{i}$ of Section 2.4: the safety factor governs when a junction fails, and $\Theta_{\mathrm{sys}}$ governs how many must fail before weakness is detectable. It therefore depends on the endpoint, no measurement of it exists, and $\Theta_{\mathrm{sys}}=0.60$ is a declared assumption carried with its map. Its influence on the falsifiable form is a cancellation rather than a small partial derivative: over [0.30,0.80], both ${\bar{C}}_{\mathrm{crit}}$ and K move by more than 100% against 24% for their ratio (Supplementary Materials S1.13).

Under a uniform field, the $S_{i}$ are independent and identically distributed, so NΦ is binomial and P(Y=1) is available in closed form; the Lindeberg–Feller theorem [30,31] supplies the normal approximation used in Section 3, with Berry–Esseen bounds in Supplementary Materials S2.

### 2.7. Gradedness of the Collective Readout

Equation (8) makes the readout a step in Φ. A clinical endpoint is not: an observer-rated scale is graded, carries measurement error, and is dichotomised at analysis, so the probability of being classified as a responder is P(Y=1∣Φ)=G(Φ) with *G* smooth. Section 4.3 asked the corresponding question one level below and found the binary idealisation inadequate; the same question here has a different answer. Convolving the step with a smooth readout leaves the $N^{-1/2}$ term intact and adds an *N*-independent one, of the same form and the same consequence as the between-subject dispersion of Equation (23). The idealisation is adequate precisely where the paper needs it—at N=20–100, where the finite-size band spans 22–49% of the critical dose—and inadequate at $N=10^{4}$, which is the regime Section 4.8 already excludes from testing on other grounds. A readout of unknown gradedness is therefore not a separate limitation but the third contaminant of that design (Supplementary Materials S1.22).

### 2.8. Multiple Endplates per Fibre: A Second Nested Layer

Human facial muscles depart from the classical innervation pattern in a way that bears directly on what *N* counts. Acetylcholinesterase staining of ten mimetic muscles in eleven cadavers shows motor endplates distributed outside a narrow band and, on teased single fibres, more than one endplate per fibre [32]; in facial and laryngeal muscles, these endplates lie typically under 150 μm apart [33], and serial sectioning with double staining establishes that closely spaced endplates on one laryngeal fibre are supplied by a single motoneuron [34].

Sibling endplates share the neuronal determinants of their threshold and one local concentration but take up toxin independently, and a fibre fails only if all *k* of its endplates fail:

(9) $$p_{\mathrm{fib}}\left(C\right)\;=\;E_{\theta }\;\left[\;q{\left(\theta ,C\right)}^{\;k}\;\right],\;q\left(\theta ,C\right)\;=\;P\;\left(K\geq \left\lceil -\ln(1-\theta )/\eta \right\rceil \right),$$

with K∼Poisson(λC). The readout unit is therefore the fibre, the count entering Proposition 1 is $N_{\mathrm{fib}}=N_{\mathrm{term}}/k$, and no value of *k* is claimed for any named muscle, so results are given as functions of *k* throughout (Supplementary Materials S1.11, which gives the full hierarchy and the exact evaluation).

### 2.9. Spatial Concentration Field

Geometry enters through the field $\left\{C_{i}\right\}$ at fixed spatial mean $\bar{C}$. Terminals are taken uniformly distributed on a disc of radius *R*, the injection is centred, and the concentration profile is Gaussian with dispersion radius *s*:

(10) $$C\left(r\right)=\frac{\bar{C}\;e^{-r^{2}/2s^{2}}}{{\langle e^{-r^{2}/2s^{2}}\rangle }_{\;R}},\;0\leq r\leq R,$$

normalised so that the mean over terminals equals $\bar{C}$ exactly for every *s*. The limit s/R→∞ recovers the uniform field; decreasing s/R concentrates the same total dose progressively. The kernel replaces the point-mass construction used in earlier formulations of this class of model, in which the geometry effect is a restatement of mass conservation rather than a property of the response (Supplementary Materials S1.19); its natural summary is the critical dispersion radius $s^{*}$ of Section 4.2, which maps onto injection volume and dilution.

Two bodies of work bear on this and agree. Injecting the same dose in a larger volume produces a measurably larger area of effect [35], the empirical counterpart of varying *s* at fixed $\bar{C}$; and multiscale transport models of BoNT-A in the glabella find injection volume to be the dominant determinant of spread [36]. They describe one trade-off from opposite sides: spread too small, it leaves the target under-covered, which is the regime Proposition 2 quantifies through $s^{*}$, while spread too wide, it reaches neighbouring muscles. The present model addresses only the first, and represents transport by a one-parameter kernel rather than by tissue mechanics.

### 2.10. Temporal Scope

The model as constructed has no time axis within a treatment cycle. The cleaved fraction $f_{i}$ of Equation (3) is the value at the moment of readout, and the per-event yield η absorbs the post-internalisation reaction time; nothing in Section 2 and Section 3 represents onset, plateau or recovery, and the extensions of Section 4.9 step between cycles rather than through one. Section 4.10 adds a minimal recovery law and asks what the architecture then implies about the duration of effect; what it delivers, and what it fails to deliver, are stated there. The absolute quantities are conditional on the readout time; the falsifiable content is not, because a persistence acting as a common gain leaves *W* invariant (Supplementary Materials S1.2). This omission is material for a comparison between serotypes, because the light chain of BoNT-A persists in the cytosol for months while that of BoNT-E is cleared within days [1,24]: two preparations assayed at a common time post-exposure are sampled at different points of their own time courses.

## 3. Theoretical Properties

We first dispose of two properties that look like results but are not, then state the two that are. Proofs are in Supplementary Materials S2.

### 3.1. The Comparison Class

Two benchmarks are needed, and they do different work. The first is a stochastic mean-only mapping $Y^{B}∼\mathrm{Bernoulli}\left(g\left(\bar{C}\right)\right)$ rather than the deterministic $R=g(\bar{C})$ that such comparisons usually adopt, because a deterministic benchmark has zero variance everywhere and any stochastic model beats it vacuously (Supplementary Materials S2.3). Its role is negative: it establishes what a mapping without a unit count cannot express.

The second benchmark is the one that carries the discriminating content, and it is not a mean-only mapping at all. Architectures in which a collective outcome follows from a finite population of independently failing units are established in several fields, and they differ from the present model in one respect that turns out to be decisive: whether the collective threshold is an absolute count or a fraction of the population. Three published families take the first option. In multi-hit target theory, the outcome follows once *n* hits accumulate at a fixed target. In the critical subset model of antiviral pharmacodynamics, replication requires at least *c* functional copies of a target with *c* fixed as the copy number varies. In the multi-unit, multi-path model of anaesthetic potency [37], immobility requires that every one of *m* conduction pathways fail, each pathway failing once any one of its *n* units is blocked. The present model takes the second option: block follows once the silenced fraction reaches $\Theta_{\mathrm{sys}}$, so the threshold scales with the population rather than sitting at a fixed count.

The distinction is not a matter of parameterisation. It places the two classes in different asymptotic regimes—extremes and fixed counts belong to extreme-value and Poisson-tail behaviour, a fixed fraction to the central limit —and it produces opposite predictions for a quantity that every dose-ranging study measures. Section 3.8 sets those predictions out.

### 3.2. An Absolute Threshold Is Excluded by Clinical Practice Alone

The preceding comparison requires a measurement. One consequence of the same distinction does not.

Under the quantal terminal response of Section 4.3, a unit with threshold θ has per-impulse failure probability ${\left[1+\theta^{\gamma }\right]}^{-1}$ even at complete cleavage, so the silenced fraction is bounded above by $\phi_{\max}=0.9787$ at the human reference and not by unity. That ceiling is a property of the quantal derivation and not of the model’s distributional choices, and it has a consequence for absolute thresholds that fractional thresholds do not share.

If a block requires N−c+1 units silenced with *c* fixed, then the required fraction is (N−c+1)/N, which exceeds $\phi_{\max}$ once

(11) $$N>\frac{c-1}{1-\phi_{\max}},$$

beyond which a block is unattainable at any dose. The threshold size is sensitive to $\phi_{\max}$ in a way the qualitative claim is not, because $1-\phi_{\max}$ is a difference between quantities near unity. Evaluated over the nine combinations of the response steepness admitted by quantal content (${\bar{m}}_{0}=20$–30, hence, γ=5.7–6.9) and the mean threshold over its declared range ($\mu_{\theta }=0.40$–0.60), the bound of Equation (11) at c=21 runs from N≈330 to N≈5900 over the nine cells, evaluated at the rounded γ=5.7–6.9; the exact Poisson values move the upper end to N≈5830. That is a factor of eighteen either way, and the sensitivity is carried almost entirely by $\mu_{\theta }$ rather than by γ. The threshold size is therefore a few hundred to a few thousand units and should not be quoted more precisely. What does not move is the existence of the bound: in none of the nine combinations does $\phi_{\max}$ reach unity, the smallest value of $1-\phi_{\max}$ being 0.0034, so a finite threshold size exists throughout. For every fixed *c*, there is therefore a target size above which the toxin cannot work. Botulinum toxin type A produces functional block across targets whose independent-unit counts differ by orders of magnitude, from the small perioral muscles to large limb muscles treated for spasticity. No single absolute count is compatible with that range. Either the collective threshold scales with the population, or the toxin should fail in large muscles and does not.

The argument requires no dose–response measurement, no estimate of *N* beyond its ordering, and no new experiment. It uses only the existence of the clinical practice. We note that it turns on the same ceiling that Section 2.3 treats as a defect when it arises from an unbounded threshold law: the two are not the same object, and the distinction is stated there.

### 3.3. Observation 1—The Variance Peak Is a Bernoulli Identity

Since *Y* is binary, Var(Y)=P(Y=1)(1−P(Y=1)), maximised at 1/4 when P(Y=1)=1/2. The concentration at which this occurs, ${\bar{C}}_{\mathrm{crit}}$, is defined by $E\left[\Phi \left({\bar{C}}_{\mathrm{crit}}\right)\right]=\Theta_{\mathrm{sys}}$ and is simply the median effective concentration of the binary readout.

We state plainly that this is not a result. It is an identity of the Bernoulli distribution; it holds for the benchmark of Supplementary Equation (S34) identically, and describing it as a critical transition, a phase transition, or an amplification cascade imports statistical-physics vocabulary onto an arithmetic fact. The genuine content of a finite discrete population appears not in the height of the variance maximum, which is fixed at 1/4 by construction, but in its width, which is the subject of Proposition 1.

### 3.4. Observation 2—Mean-Curve Reproduction Is Not Discriminating

By construction, *g* can be calibrated to reproduce $P(Y=1|\bar{C})$ under a uniform field to arbitrary accuracy. Agreement between the discrete model and a calibrated Hill curve on the uniform-field dose–response therefore carries no evidence about architecture, and we do not present it as such.

### 3.5. Four Statements Withdrawn

Observations 1 and 2 are two of four statements that earlier formulations of this framework advanced and that we do not advance here; the third concerns the cleavage–effect correlation coefficient, and the fourth the route by which the serotype ordering was derived, which is superseded although the ordering itself is retained (Section 4.5). For two reasons, we collect them in Supplementary Table S8 rather than leaving them where each arises: They are the claims most easily mistaken for evidence in this class of model, so a reader is entitled to find them in one place. And they are categorically different from the conditional quantities of Table 2: a conditional quantity is a number this paper reports subject to a stated dependence, and a future measurement updates it by substitution; a withdrawn claim is an inference this paper does not make at all, and no measurement reinstates it. The distinction is not rhetorical. The hedged formulations throughout this paper are of the first kind unless Supplementary Table S8 says otherwise.

### 3.6. Proposition 1—Sharpness Law Linking N and Threshold Dispersion

Let $p\left(\bar{C}\right)=P\left(f_{i}\geq \theta_{i}|\bar{C}\right)$ be the terminal silencing probability under a uniform field, continuously differentiable and strictly increasing near ${\bar{C}}_{\mathrm{crit}}$, and let ${\bar{C}}_{q}$ solve $P(Y=1|{\bar{C}}_{q})=q$. Then, for q∈(0,1) and $\Delta {\bar{C}}_{q}:={\bar{C}}_{q}-{\bar{C}}_{1-q}$ with q>1/2,

(12) $$\begin{array}{c} \;\Delta {\bar{C}}_{q}\;\sqrt{N}\;⟶\;\frac{2\;z_{q}\;\sqrt{\Theta_{\mathrm{sys}}\;\left(1-\Theta_{\mathrm{sys}}\right)}}{p^{\prime }\left({\bar{C}}_{\mathrm{crit}}\right)}\;=:\;K\; \end{array}$$

as N→∞, where $z_{q}$ is the standard normal quantile at *q*. The limit K is independent of *N*.

Proof sketch. $N\Phi ∼\mathrm{Binomial}(N,p\left(\bar{C}\right))$, so $P\left(Y=1\right)=\Psi \;\left(\sqrt{N}\;\left[p\left(\bar{C}\right)-\Theta_{\mathrm{sys}}\right]/\sqrt{p(1-p)}\right)+O\left(N^{-1/2}\right)$ by Lindeberg–Feller, with Ψ being the standard normal CDF and the error controlled by the Berry–Esseen bound [38]. Linearising *p* about ${\bar{C}}_{\mathrm{crit}}$, where $p=\Theta_{\mathrm{sys}}$, and solving for the quantiles gives Equation (12). Full argument in Supplementary Materials S2.

The closed form is accurate to the first order in $N^{-1}$ rather than $N^{-1/2}$, because the two quantiles are displaced in the same direction and the difference gains a further half order (Supplementary Materials S2.8).

Three consequences carry the weight of this paper.

(i) The law is quantitative, not merely qualitative. It predicts not that transitions sharpen with *N*, but by exactly how much: the product $\Delta {\bar{C}}_{q}\sqrt{N}$ is constant. Numerically, K=0.378 at the reference parameters with q=0.75, and Equation (12) reproduces the exact binomial widths with relative error 1.08/N—better than 0.54% across N=200–50,000 (Section 4.1).
(ii) It cannot be expressed by a mapping in mean concentration alone. The claim is that *N* must enter the mapping, and that once it does, the relation between targets is fixed rather than free (Supplementary Materials S2.8, which also derives the effective count $N_{\mathrm{eff}}$ under which the exponent survives correlated units). The benchmark $g(\bar{C})$ contains no *N*. It can be calibrated to any single target, reproducing that target’s transition exactly; it then makes no prediction whatever for a target with different *N*, whereas Equation (12) fixes the second target’s transition with no free parameters once the first is calibrated. The magnitude is not marginal: a surrogate matched at N=10,000 mispredicts the transition width by −92.8% at N=50 and, by the same relation, by +123.6% at N=50,000; Supplementary Figure S2 plots the error over $N=10^{2}$–$10^{4}$. This is the sense in which the architecture is not redundant, and it is a testable difference rather than a definitional one.
(iii) It makes threshold dispersion estimable. The prefactor depends on $\sigma_{\theta }$ through $p^{\prime }\left({\bar{C}}_{\mathrm{crit}}\right)$: broader dispersion flattens *p* and widens the transition at every *N*.

Since $\sigma_{\theta }$ cannot be recovered from a single dose–response curve—where it is confounded with κ and $\Theta_{\mathrm{sys}}$—while the *N*-dependence of the width breaks that confounding, the law converts a microscopic parameter into an estimable one. Section 4.7 verifies this by synthetic recovery.

### 3.7. Corollary—A Scale-Free Form That Is Measurable in Administered Dose

Equation (12) is stated in local concentration, which is not observed. The corresponding statement in relative width is, and it is free of the quantities this model does not measure. Define

(13) $$W\;:=\;\frac{\Delta {\bar{C}}_{q}}{{\bar{C}}_{\mathrm{crit}}}\;\sqrt{N}\;=\;\frac{K}{{\bar{C}}_{\mathrm{crit}}}.$$

Suppose the administered dose *D* maps to local concentration by a target-specific gain, $\bar{C}=cD$. The model depends on dose only through the product λcD at fixed per-event yield η, so *c* rescales the dose axis and nothing else: both ${\bar{C}}_{\mathrm{crit}}$ and K scale as $c^{-1}$ and *W* is invariant. Hence,

(14) $$W\;=\;\frac{\Delta D_{q}}{D_{50}}\;\sqrt{N},$$

with $D_{50}$ the median effective dose in the sense of [39] and $\Delta D_{q}$ the interquantile width of the responder curve—both read directly off a dose–responder experiment, with no diffusion, potency or pool-fraction calibration.

Five quantities that do not enter. The consequence worth stating in one place is how much *W* does not depend on. Anything acting as a multiplicative gain on effective exposure leaves it unchanged, and four of the five quantities this model cannot measure are such gains: the uptake–cleavage composite κ, which cancels identically in Supplementary Equation (S44); lot potency; the terminal pool fraction ρ of Section 2.5, on which every absolute concentration in this paper depends; and the readout time relative to a serotype’s intracellular persistence (Section 2.10). The fifth is not a gain at all: the shape of the threshold law, which moves the mean-field prefactor by 9.1% across three distributional families at matched moments and moves *W* by 0.25%, because Poisson uptake convolves the law before the collective integration (Section 3). A relation of this kind is worth having precisely because it survives the parameters nobody can supply; it is what makes the law transportable between targets rather than re-fitted at each.

This also settles in advance the question a pharmacometric reader asks first, and the answer is worth stating affirmatively rather than leaving it to be inferred. The model is not identifiable in its microscopic parameters, and we do not claim that it is: the uptake count scale and the per-event yield are separable only through the weak curvature that uptake stochasticity adds, and at achievable noise their product is bounded only to within a factor of 5.7 (Section 4.7). The falsifiable content does not rest on estimating any of them. It rests on an invariance—the constancy of *W* across targets—which is a restriction relating two observed curves rather than a value to be fitted, and which is therefore testable whether or not the parameters generating it can ever be separated. Identifiability would be required of a model used to estimate a mechanism; it is not required of one used to forbid a relation between two experiments.

The same statement in standard pharmacodynamic language. Sharpness is conventionally carried by the Hill exponent *n*, which in pharmacodynamic practice is a fitted shape parameter without mechanistic content [13]. Matching the interquartile ratio of a Hill curve, $D_{0.75}/D_{0.25}=3^{2/n}$, to Equation (14) gives, for narrow transitions,

(15) $$n_{\mathrm{eff}}\;=\;\frac{2\ln3}{W}\;\sqrt{N},$$

so the architecture predicts that the effective Hill exponent of a responder-rate curve scales as the square root of the independent-unit count, with coefficient 2ln3/W=2.382 under the quantal response of Section 4.3 and 3.723 in the binary limit. This is Proposition 1 restated in the quantity pharmacodynamic practice already fits, and it makes the test concrete: fit a Hill curve to responder rate against administered dose in targets of differing size; the ratio $n_{\mathrm{eff}}/\sqrt{N}$ should be constant across targets. We recommend reporting $W=\left(\Delta D_{q}/D_{50}\right)\sqrt{N}$ directly, which is convention-free.

### 3.8. The Discriminating Prediction: Location, Not Steepness

The three architectures of Section 3.1 share the exponent of Proposition 1, and the reason they nonetheless make opposite predictions is elementary. Under a fractional threshold, the silenced fraction required for block is $\Theta_{\mathrm{sys}}$ whatever the population size, so ${\bar{C}}_{\mathrm{crit}}$ solves $p\left(\bar{C}\right)=\Theta_{\mathrm{sys}}$ and carries no *N*: the median effective dose is a property of the per-unit curve alone. Under a fixed reserve of *c* units, the required fraction is (N−c+1)/N, which rises towards unity as the population grows, so the dose must rise with it. Under multi-hit target theory, the median sits at $\lambda_{50}\approx n$, so the dose is very nearly proportional to the number of hits required—but *n* counts hits at a target and not units in a muscle, so that family is separated by the absolute width rather than by the location. Against the fixed-count architecture, which is the one with an established pharmacodynamic instantiation, potency and not steepness is where the two divide. The discriminator is a comparison of scaling and not of level, so it survives the endpoint dependence of $\Theta_{\mathrm{sys}}$; what it does not survive is the mixing of endpoints across the targets compared, which is a constraint on the experiment and not on the claim (Supplementary Materials S6.6, which also records the one coefficient that does not discriminate).

**Figure 1:**
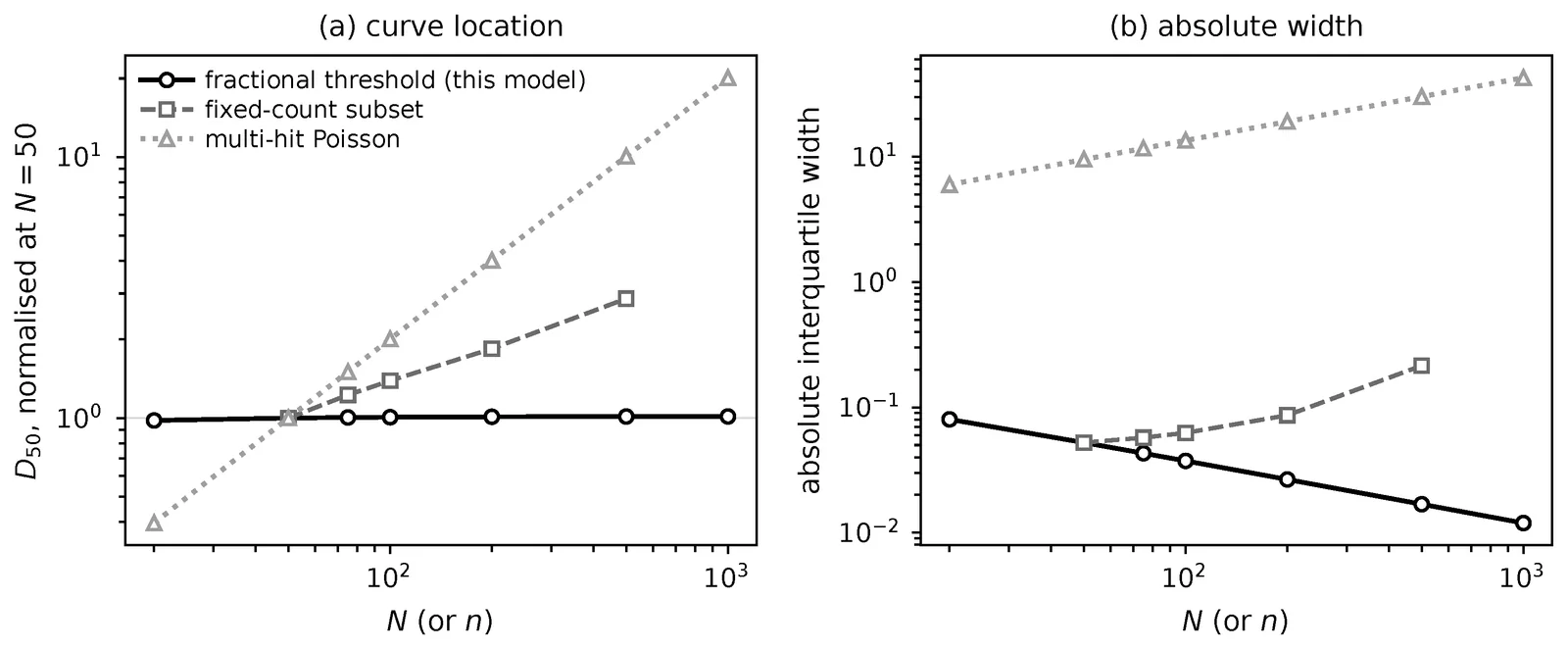
The three architectures across target size, on logarithmic axes. (**a**) Median effective dose, normalised at N=50. The fractional threshold is flat; the fixed-count and multi-hit families rise, the latter very nearly in proportion to the number of hits required. (**b**) Interquartile width in absolute dose units. The fractional width contracts and the two alternatives expand, so the discriminator does not depend on any normalisation of the dose axis. Values are those of Supplementary Table S22; the fixed-count family is plotted only over the range on which it is defined.

The residual *N*-dependence of the fractional architecture is not a weak trend but a finite-size correction of the order the theory requires: the product of relative displacement and *N* is constant to within 4.1% across the whole range, and $D_{50}$ converges on the mean-field value from below. The second row of the comparison needs no normalisation at all, because it is read in units of administered dose: the interquartile width contracts under a fractional threshold and expands under both alternatives, with slopes of opposite sign (Figure 1). Two targets differing in size, measured on the same dose scale, separate the families without any estimate of local concentration.

This also locates the relation to [40] precisely rather than by assertion. Their asymptotics are taken in the tail, where the response is linear in the counts; the expansion of Proposition 1 is taken at the median, where it is not. The two describe different regions of the same curve, and what separates the models is not the region but the location of the median. The smallest displacement detectable at the steepest responder curve the published literature supplies, with forty subjects per arm, is a factor of 2.32 (Supplementary Table S23): comfortably inside the multi-hit prediction, marginal against the fixed-count one, which, in any case, Section 3.2 excludes without measurement.

### 3.9. Proposition 2—Geometry Non-Sufficiency at Matched Mean Dose

For the diffusion field of Equation (10), there exist dispersion radii $s_{A}\neq s_{B}$ with identical spatial mean $\bar{C}$ such that

(16) $$P\;\left(Y=1|\bar{C},s_{A}\right)\neq P\;\left(Y=1|\bar{C},s_{B}\right),$$

so the spatial mean is not a sufficient statistic for system output. Equivalently, for each dose, there is a critical dispersion radius

(17) $$s^{*}\left(\bar{C}\right):=\inf\left\{\;s:P\left(Y=1|\bar{C},s\right)\geq 1/2\;\right\},$$

and the block is unattainable for $s<s^{*}$ however the dose is placed.

Non-sufficiency of the spatial mean is an instance of Jensen’s inequality and we claim it as an observation rather than as a result of this architecture; what the architecture supplies is $s^{*}$ and its interior optimum in placement, and what the proposition excludes is the mean-only class in routine pharmacodynamic use (Supplementary Materials S2.4).

### 3.10. Observation 3—Finiteness, Not Stochasticity, Carries the Weight

Having set aside two properties that do not discriminate, it is worth naming the ingredient that does, because two natural candidates do not. Neither the stochasticity of the output nor, at k=1, that of uptake carries the behaviour (Supplementary Materials S1.11).

Output stochasticity does not, because the benchmark of Supplementary Equation (S34) is itself stochastic. Uptake stochasticity does not either, at k=1: there, it is a robustness check on an approximation rather than the mechanism generating the behaviour. Section 4.4 shows that this ceases to hold once a fibre carries more than one endplate, where the same shift reaches 39.8%.

Discreteness does. The population is finite and countable, and *N* enters Equation (12) while appearing nowhere in any mapping of mean concentration. That is the only asymmetry surviving the benchmark of Supplementary Equation (S34), and it is what makes the law transportable between targets and therefore falsifiable. The operative contrast is not stochastic against deterministic; it is finite *N* against mean field.

### 3.11. What Is Prior, and What Is Not

The relation of Proposition 1 is not new. The mathematics is standard—the delta method on a binomial quantile, the *k*-out-of-*N* system of reliability theory, the *m*-out-of-*n* fusion rule of distributed detection and the $\sqrt{N}$ gain of an independent population [41,42,43,44,45]—and the architecture is prior in pharmacodynamics, in the critical subset model of antiviral action [40,46] and in the demonstration that the apparent Hill exponent carries no mechanistic interpretation [47,48,49]. We claim no novelty for any of it, and Supplementary Materials S2.4 attributes each layer in full.

The exponent is prior in biology as well. A dose–response curve of the form P(Poisson(λ)≥n) is the single-target multi-hit model of radiobiological target theory [50,51], and its relative transition width contracts as $n^{-1/2}$ by the same argument. The equivalent Hill exponent therefore already grows as the square root of a biological unit count in a published dose–response framework, whether or not that consequence was drawn there.

What is not prior. Three things. The asymptotic form at the operating point, with the prefactor $2z_{q}\sqrt{\Theta_{\mathrm{sys}}\left(1-\Theta_{\mathrm{sys}}\right)}/p^{\prime }\left({\bar{C}}_{\mathrm{crit}}\right)$ identifying the constant with threshold dispersion: the published asymptotics of [40] are tail asymptotics, linear in the counts, and describe a different region of the same curve (Section 3.8). The claim is about the form of the expansion and not about the value of the constant, which does not by itself distinguish the architectures, as shown in Section 3.8. The gain-invariant form *W* of Section 3.7, and with it transportability between targets rather than refitting at each. And the consequence that separates a fractional collective threshold from the fixed-count and extreme-threshold families: potency invariant to the unit count, absolute transition width contracting rather than widening. Whether ref. [47]’s heterogeneity term and the count term derived here are the two determinants of the apparent exponent, and not one, is the substantive claim; his analysis is taken in the infinite-population limit, where the second does not arise. The architecture is accordingly not specific to botulinum toxin: Proposition 1 uses a finite population of units failing independently, a per-unit threshold with dispersion, and a readout that is a threshold on the fraction silenced, and nothing else (Supplementary Materials S2.2). We instantiate it on upper-face BoNT-A because that is where the three quantities can be anchored to published measurement: the safety factor of neuromuscular transmission fixes the threshold, quantal content fixes the steepness, and motor-unit number estimation makes the unit count accessible.

### 3.12. Theoretical Predictions

**Figure 2:**
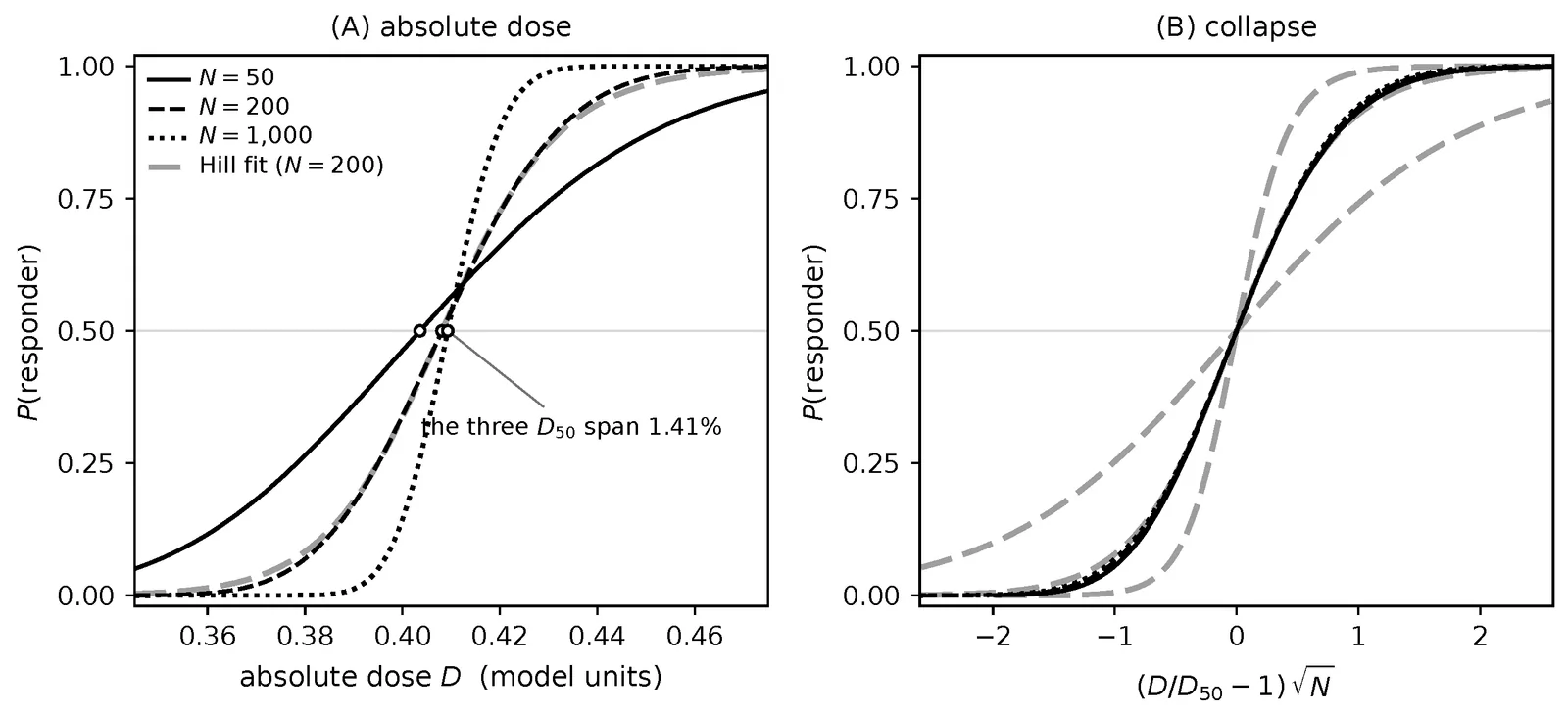
The sharpness law as a collapse. (**A**) Responder rate against absolute administered dose in model units, for three targets differing only in independent-unit number, at the human reference under the quantal response. The dose axis is not normalised by each curve’s own midpoint, so the near-coincidence of the three midpoints is a result and not an artefact of the axis: they span 1.41% over a twentyfold change in *N*, converging on the mean-field critical concentration from below, while the widths contract by more than a factor of four over the same range. This is the discriminating signature of Section 3.8 seen directly; under a fixed-count collective threshold, the midpoints separate with *N* instead (Table 3). (**B**) The same three curves with the dose axis centred and rescaled by $\sqrt{N}$; they collapse onto one, with no parameter adjusted to make them do so. The grey dashed curve in both panels is a single Hill function calibrated on the intermediate target and applied unchanged to the other two: it mispredicts the width in (**A**) and fails to collapse in (**B**), which is Corollary 2 of Supplementary Materials S2.5 seen directly. The collapse, and not the value of the constant onto which it collapses, is what the test of Section 3.12 interrogates.

1. Sharpness collapse. Responder-rate transitions across targets differing in independent-unit number, expressed as the scale-free product $\left(\Delta D_{q}/D_{50}\right)\sqrt{N}$ of Section 3.7—equivalently as $n_{\mathrm{eff}}/\sqrt{N}$—collapse onto a single constant. The product is invariant to the dose–concentration gain, so the test requires no estimate of local concentration, and a width independent of *N* falsifies Proposition 1 (Figure 2). The exponent is a weaker instrument than it appears, since the design of Section 4.8 separates −1/2 from 0 but not from neighbouring values; discrimination between architectures therefore rests on the location of the curve (Section 3.8). The test is a parametric restriction with J−1 degrees of freedom across *J* targets, and it isolates *N* only if the prefactor is common across them—which makes quantal content and the number of endplates per fibre the two contaminants that must be matched (Supplementary Materials S6.4).
2. Potency invariant to target size. This is the prediction that separates the architectures, and it is read off the location of the curve rather than its steepness. Because the required silenced fraction is $\Theta_{\mathrm{sys}}$ whatever the population size, $D_{50}$ is invariant to the independent-unit count (1.036× over N=20–1000), while the absolute interquartile width contracts (slope −0.4887); a fixed-count collective threshold requires the opposite in both columns (2.867× and +0.6149), and the multi-hit family likewise in the width (Table 3, Figure 1). The comparison is in administered dose and needs no concentration calibration. One half of it requires no measurement at all: Section 3.2 excludes every fixed count above a target size of a few hundred to a few thousand units from the existence of clinical practice alone.
3. Critical dispersion radius, and an optimal ring radius. At fixed mean dose, block fails below a critical spatial dispersion, and $s^{*}$ decreases only weakly with dose, so spatial spread and dose escalation are not interchangeable (Section 4.2). Under fractionated deposition $s^{*}$, it is non-monotone in the placement radius, with an interior optimum near two-thirds of the target radius: placement further towards the periphery raises the dispersion required for block rather than lowering it. The prediction is directional, requires no concentration calibration, and is tolerant of a placement error of five percent of the target radius. It is also the geometric prediction that survives both limits of Section 4.2; the ordering of fractionation against dose escalation does not, and is a prediction about volume rather than about site count.
4. Serotype asymmetry in the cleavage–effect relation. Under a collective threshold, the outcome transition occupies a band of measured cleavage of width $\rho \;h\left(\langle f\rangle \right)W/\sqrt{N}$, and the dominant-negative strength that distinguishes the two serotypes moves it: at the value the pool-fraction reading fixes, BoNT-A’s band is 2.41 times narrower than BoNT-E’s, so BoNT-A should show the weaker cleavage–effect correspondence, which is the reported pattern [1,23]. The ordering holds under an assay whose reproducibility is an absolute error and reverses under one whose reproducibility is a coefficient of variation; that is, a property of the assay, separately measurable, and the prediction carries the condition explicitly (Section 4.5). A mapping of mean concentration alone offers no account of why the same substrate, cleaved by two toxins, yields correlated and uncorrelated dose–effect relations.

Predictions 1 and 2 require no single-terminal resolution: responder status against dose, in targets of differing size, suffices. They do not follow from aggregate parallel-group data, however, and require the hierarchical design that Section 4.8 derives—a longitudinal titration across targets of differing independent-unit count, for which no protocol has, to our knowledge, been specified in human muscle (Supplementary Materials S6.3–S6.4).

## 4. Results

**Figure 3:**
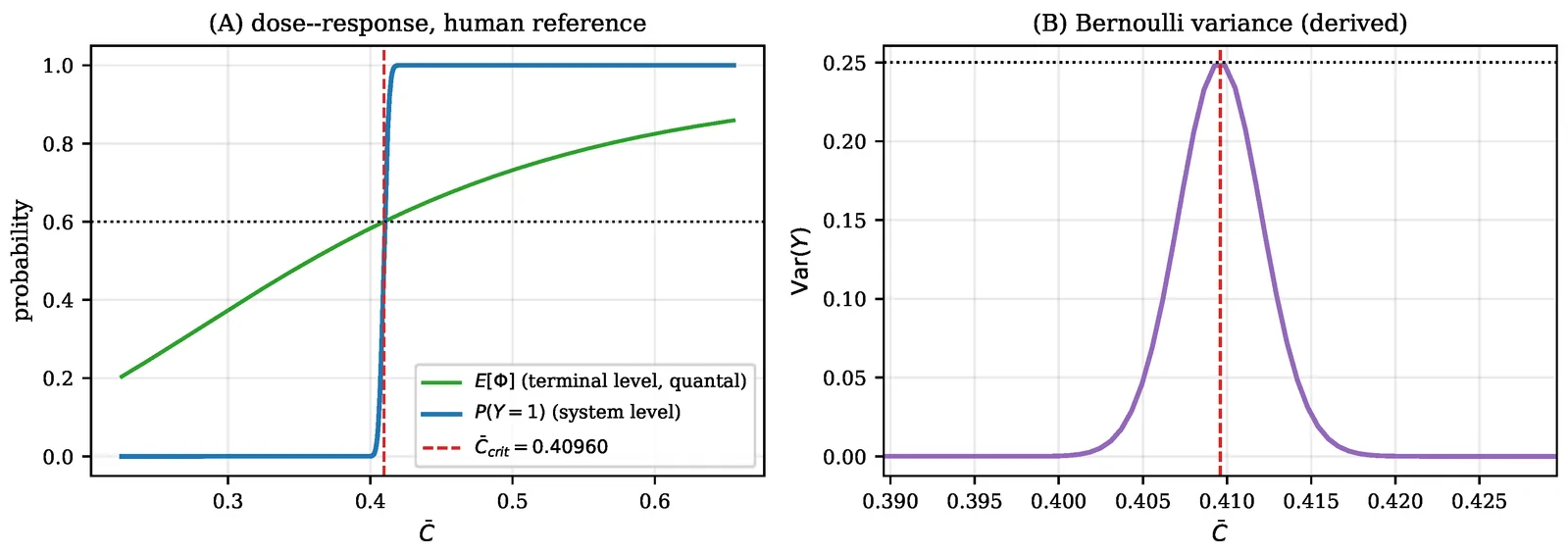
Dose–response and output variance. (**A**) Terminal-level E[Φ] and system-level P(Y=1) against $\bar{C}$; the dotted line marks $\Theta_{\mathrm{sys}}$ and the dashed line ${\bar{C}}_{\mathrm{crit}}=0.4096$. (**B**) Var(Y) near the critical point. The maximum at 1/4 is the Bernoulli identity and carries no structural information (Observation 1); the informative quantity is the width of the peak, not its height. Purple curve = Var(Y) under stochastic-uptake formulation; red dashed line = theoretical maximum of 1/4.

Unless stated otherwise, all results use the reference configuration κ=2.0, λ=35 (hence η=0.0571), $\mu_{\theta }=0.50$, $\sigma_{\theta }=0.075$, $\Theta_{\mathrm{sys}}=0.60$, N=10,000 (Supplementary Table S7). Under these values, the critical concentration is ${\bar{C}}_{\mathrm{crit}}=0.3862$ in the stochastic-uptake formulation and 0.3663 in its mean-field reduction, a difference of 5.4%. Those are the values in the binary limit; under the quantal response derived in Section 4.3, the critical concentration is 0.4096 (Figure 3). Both readings are used below and each result states which: the binary limit carries the geometry results and the uptake sweep, where the mean-field comparator is defined and where the terminal response is not at issue, and the quantal response carries the sharpness law and everything derived from it. Mixing the two silently is the commonest way to misread this model, and we have avoided it by labelling every table.

### 4.1. The Sharpness Law

The unit number varies by more than two orders of magnitude across injection targets, and the law is reported over N=200–50,000 because that is the range over which the closed form is verified. The count entering Equation (12) is the independent-unit count and not the anatomical terminal count; the operative range is $N\approx 10^{1}$–$10^{3}$, and motor-unit number estimation is the estimate we recommend (Supplementary Materials S1.4, and Section 6). The two differ in evidential status as well as in magnitude, and the ranges below should be read accordingly: terminal counts for human facial muscles are not measured but inferred from muscle mass and an assumed innervation ratio [32], whereas motor-unit numbers are measured, and have been measured directly in a facial target [52]. The constructive consequence is that the quantity the law requires is the one that is accessible, subject to the qualification of Section 6.

We computed the exact binomial transition width $\Delta {\bar{C}}_{0.75}={\bar{C}}_{0.75}-{\bar{C}}_{0.25}$ across this range and compared it with the closed form of Equation (12) (Supplementary Table S12, Supplementary Figure S1).

Under the quantal response, which is the reported case, the product is constant at $K_{q}=0.378$ to three significant figures above *N* = 1000, and the closed form remains accurate to 0.54% even at N=200, where the normal approximation is weakest. The binary limit gives $K_{\mathrm{bin}}=0.228$ and is retained in the table as a reference only; all values quoted in prose below are the quantal ones. This is the paper’s principal quantitative relation, though not its contribution (Section 3.11), and Section 3.8 states what it does and does not discriminate. The closed form is accurate to $O(N^{-1})$ rather than to the $O(N^{-1/2})$ that the Berry–Esseen term alone would suggest, and the same finite-size residual governs the error a mean-only mapping makes when carried from one target to another; both are verified term by term in Supplementary Materials S2.6. The same result read in administered dose units gives the scale-free width of Section 3.7, which converges to W=0.9226 and is within 1% of that value from N=200 upward (Supplementary Table S13); *W* is a value at the reference parameters and not a central estimate, and its dependence on threshold dispersion is set out in Supplementary Materials S2.6.

Two readings follow. Clinically, the 5–95% band of outcome uncertainty spans 48.7% of the critical dose at N=20 against 7.1% at *N* = 1000—both exact binomial inversions rather than evaluations of the closed form, since N=20 lies below the range over which that form is verified—a sevenfold difference, with the perioral figure comparable to realistic inter-session dose and placement variation. Outcome variability at nominally identical doses is therefore expected to be concentrated in small targets, which is where injection precision is hardest. Methodologically, the collapse of $\Delta {\bar{C}}_{q}\sqrt{N}$ onto a constant is the falsifiable signature: a mean-only mapping calibrated to one target predicts nothing about another, whereas Equation (12) predicts the second target’s width with no free parameters.

### 4.2. Geometry and Fractionated Deposition

Under the diffusion field of Equation (10) at fixed spatial mean, the silenced fraction depends strongly on the dispersion radius: at $\bar{C}=1.5\;{\bar{C}}_{\mathrm{crit}}$, P(Y=1) switches from 0 to 1 between s/R=0.40 and s/R=0.45. Mean concentration alone is therefore not sufficient to determine the outcome, establishing Equation (16). The dose dependence of the critical dispersion radius $s^{*}$ is weak and strongly sublinear—quadrupling the dose from 1.1 to 4.5 times the critical concentration reduces the required radius by only 53%—so within the model dose, escalation is an inefficient substitute for adequate spatial distribution, which is consistent with the clinical observation that dilution volume and placement influence outcome independently of total units [53]. This is a structural correspondence and not a calibrated prediction: $s^{*}$ is expressed in units of the muscle radius, and its mapping onto injection volume requires tissue-specific diffusion data the model does not contain [36]. The field, the tabulated radii and the derivation are in Supplementary Materials S6.1.

Clinical protocols distribute the dose over three to five sites, and generalising the field to a superposition of *n* kernels at matched spatial mean (Supplementary Materials S6.2) separates two levers that clinical usage conflates. Whether fractionation dominates escalation depends on whether the dispersion radius is set by tissue diffusion or by the size of the individual bolus, and the two limits bracket the physics and disagree in sign. The reading is therefore that the lever is volume rather than site count, and fractionation helps only insofar as it raises the total volume delivered. That converges from inside the model on what the transport literature reports from outside it: injection volume is the dominant determinant of spread [35,36]. We state the condition rather than select the limit that favours the architecture.

Placement carries comparable weight and is robust to both limits: $s^{*}/R$ is non-monotone in the ring radius, with an interior optimum between 0.60R and 0.72R across every dose and site number examined, because sites placed too near the rim deposit part of the dose where there are fewer units to silence (Supplementary Materials S6.2). The optimum is broad: a placement error of ±0.05R costs under one percent, so the ring radius is poorly determined and need not be. Pushing sites further towards the periphery makes matters worse, a prediction that is directional and requires no concentration calibration.

**Figure 4:**
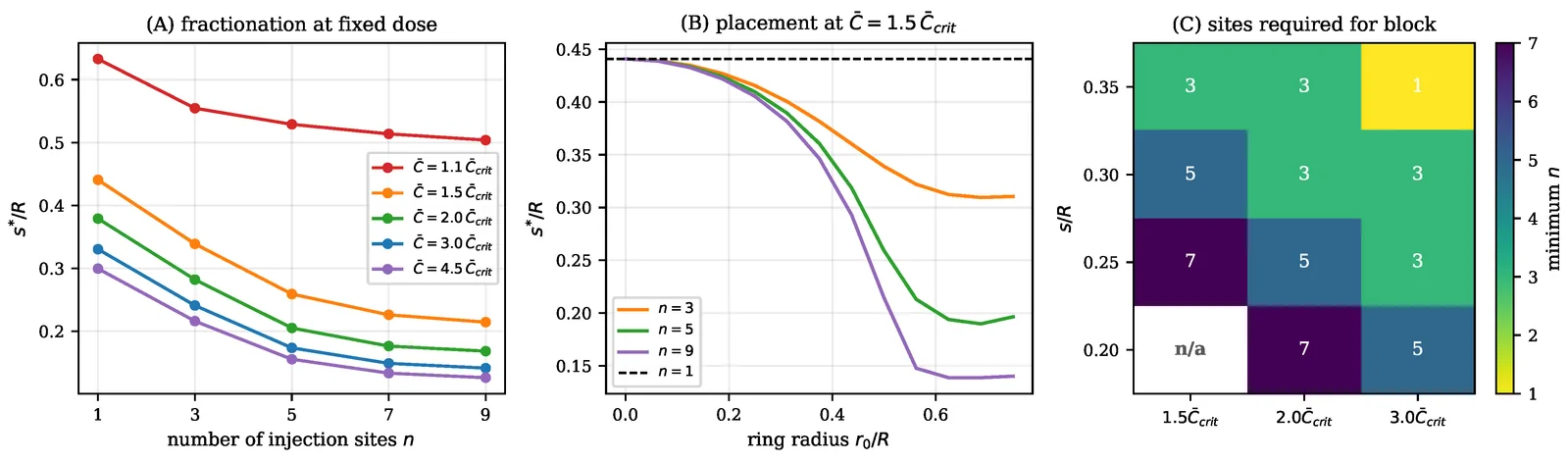
Fractionated deposition at fixed total dose. (**A**) Critical dispersion radius against number of injection sites, at five mean concentrations; the $1.5\;{\bar{C}}_{\mathrm{crit}}$ curve at n=5 falls below the $4.5\;{\bar{C}}_{\mathrm{crit}}$ curve at n=1. (**B**) Dependence on ring radius $r_{0}$ at $1.5\;{\bar{C}}_{\mathrm{crit}}$; the dashed line is the single-site value, onto which all curves collapse as $r_{0}\to 0$. (**C**) Minimum number of sites achieving block at fixed tissue dispersion; *n*/*a* marks the regime in which no n≤13 suffices.

But fractionation has a floor. At a tissue dispersion of s/R=0.20, no number of sites up to 13 achieves block at $1.5\;{\bar{C}}_{\mathrm{crit}}$, while 2.0× requires seven and 3.0× requires five (Figure 4C). The two levers are therefore not interchangeable in either direction, and the model does not license the general claim that more sites can substitute for dose. Units on a disc with sites on a ring are not the anatomy of any real target: what this establishes is the ordering of the two levers within this architecture, not a posology.

### 4.3. Graded Terminal Response

The binary indicator of Equation (4) is an idealisation. We replaced it with a graded response

(18) $$S_{i}^{\mathrm{gr}}={\left[1+{\left(\frac{\theta_{i}}{f_{i}}\right)}^{\;\gamma }\right]}^{-1},$$

which recovers the binary case as γ→∞ and a smooth proportional response for small γ, and re-derived the transition width across γ (Supplementary Table S19).

Equation (18) is a silencing probability, not a graded state: $S_{i}^{\mathrm{gr}}$ is the probability that transmission fails at unit *i*, and it is derived below by matching to the per-impulse failure probability of quantal theory.

Conditional on the field, the unit states remain Bernoulli, so Proposition 1 applies with $p_{\gamma }^{\prime }$ in place of $p^{\prime }$ and the exponent is untouched; what changes is the prefactor, and the inflation is large—66% at the reference γ, falling within 10% only for γ≳20 (Supplementary Materials S1.21, which also reports how the constant would change under the alternative reading of $S^{\mathrm{gr}}$). Rather than leave γ unspecified, we derive it from the same quantal argument used in Section 2.4.

Quantal content is itself a random variable: with $m∼\mathrm{Poisson}(\bar{m}\left(f\right))$ and failure when $m<m_{0}/\mathrm{SF}$, the per-impulse failure probability is a smooth function of the cleaved fraction whose steepness follows from the Poisson fluctuation. Expanding about f=θ gives

(19) $$\gamma_{q}\;≃\;4\;\theta \;\varphi \left(0\right)\;N_{S}\;\sqrt{{\bar{m}}_{0}\;\mathrm{SF}}\;{\mathrm{SF}}^{1/N_{S}-1},$$

with φ being the standard normal density. Two features of (19) matter. First, the derived transition midpoint returns f≈0.49–0.52 at SF=2 and 0.67–0.70 at SF=3.33, in each case reproducing $\mu_{\theta }$ rather than assuming it. Second, $\gamma_{q}$ scales as $\sqrt{{\bar{m}}_{0}}$: it is not a free parameter but a function of quantal content, which is measurable.

At the human reference (SF=2, ${\bar{m}}_{0}\approx 25$), (19) gives $\gamma_{q}=5.64$ against an exact Poisson value of 5.93 (Supplementary Table S6). That value is not determined to three figures, and we state why rather than carry the precision: because quantal content is integer-valued and ${\bar{m}}_{0}/\mathrm{SF}$ is not, the exact computation is a step function of ${\bar{m}}_{0}$ and γ oscillates by some 4% as ${\bar{m}}_{0}$ moves by one quantum (Supplementary Materials S1.20). Across the measured range ${\bar{m}}_{0}=20$–30, γ runs from 5.7 to 6.9 and *W* from 0.945 to 0.853, an excursion of 10.8% (Table 2). The reference values below are quoted at ${\bar{m}}_{0}=25$ and are conditional on it to about one part in ten. The binary idealisation is therefore not adequate at face value, and we report the consequence rather than the idealisation: the sharpness constant becomes

(20) $$K_{q}\;=\;0.378\;\mathrm{against}\;K_{\mathrm{bin}}=0.228,$$

an inflation of 65.8%. The idealisation is less adequate here than at the rodent parameterisation, where the same computation gives γ=11.2 and an inflation of 34.2%: the narrower threshold law makes the underlying step sharper, so smoothing it costs more. All transition widths reported in Section 4.1 should accordingly be read as lower bounds; Supplementary Table S12 reports both, and the clinical band at N=200 is 15.86% of the critical dose under the quantal response against 10.15% in the binary limit.

The scaling law itself is untouched: at γ=5.93, the product $\Delta {\bar{C}}_{0.75}\sqrt{N}$ is constant at 0.378 across N=200–50,000, with the ratio to the binary case fixed at 1.658 at every *N* (Supplementary Table S12). The $N^{-1/2}$ dependence originates in the binomial integration step and not in the terminal response, so γ rescales the prefactor and nothing else.

### 4.4. The Fibre Layer, and Why It Promotes Uptake Stochasticity

Supplementary Table S3 evaluates Equation (9) at the human reference. Redundancy is protective, and strongly so: from k=1 to k=5 the critical dose rises monotonically by 77.9%. A fibre carrying five endplates requires nearly twice the dose of a singly innervated one at otherwise identical parameters, because every endplate must fail independently.

What the table also shows is a cancellation. The prefactor K rises by 73.1% over the same range, almost matching the rise in ${\bar{C}}_{\mathrm{crit}}$, so their ratio moves by only 10.0% and does so without monotonicity: *W* falls from 0.9226 at k=1 to 0.8386 at k=2, is flat through k=3, and returns to 0.8975 at k=5. We state this as bounded non-monotone variation rather than as a trend, because it is not one. The exponent is untouched: at k=3, the product $\Delta {\bar{C}}_{0.75}\sqrt{N}$ is constant to 0.67% over N=200–50,000.

The dose shift obeys an approximate scaling law in CV(K), valid for k≲5 and reported as an approximation rather than a law (Supplementary Materials S1.10), and it carries a consequence for Section 3.10 that we report against our own earlier position. In the mean-field reduction, *q* takes only the values 0 and 1, so $q^{k}=q$ and the fibre layer vanishes: over a grid of four hundred concentrations, the difference between k=1 and k=5 is exactly zero, not merely small, because the identity is algebraic. Everything the layer does is therefore invisible to any mapping of mean concentration, by construction. And what it does is large. Compared at matched terminal response, the stochastic-uptake shift over the mean-field reduction grows from +5.4% at k=1 to +21.1% at k=2, +29.6% at k=3 and +39.8% at k=5—a factor of 7.3. Observation 3 of Section 3.10 is amended accordingly: finiteness carries the sharpness law, but in a multiply innervated muscle uptake, stochasticity carries the operating point, and λ ceases to be an inert declared parameter.

### 4.5. The Serotype Asymmetry in Cleavage–Effect Correlation

Section 1.3 noted that BoNT-A cleavage correlates poorly with paralysis while BoNT-E cleavage does not. Under the present architecture, this is expected rather than anomalous, and Table 4 makes the point quantitative.

The serotype contrast follows from the same construction, and the molecular difference in Section 1.3 is what sets the parameter. The retained, dominant-negative BoNT-A product enters the model at exactly one place, as the dominant-negative strength β of Supplementary Equation (S21); the BoNT-E product, which does not assemble, acts close to pure loss of function and corresponds to β≈0. The architecture does not dispense with the two molecular mechanisms; it carries them through one parameter, and the consequence is a quantitative and directional prediction rather than a qualitative appeal. Because β moves the unit threshold as well as the steepness, the prediction has to be stated in the quantity an assay actually resolves, and that is where the previous formulation of this argument went wrong (Supplementary Table S8 and Supplementary Materials S3.2).

The ordering between the serotypes cannot come from the assay, whose between-preparation dispersion is a property of the method and is therefore common to the two: it cannot by itself generate an ordering. It has to come from the width of the informative band, which is what the architecture supplies. In relative form, the pool fraction ρ cancels from that width identically:

(21) $$\frac{\Delta (\rho \langle f\rangle )}{\rho \langle f\rangle }\;=\;\frac{\ln\;\left[1/(1-\langle f\rangle )\right]\;\left(1-\langle f\rangle \right)}{\langle f\rangle }\;\frac{W}{\sqrt{N}},$$

The coefficient of Equation (21) is 0.603 at the reference configuration and spans 0.44 to 0.81 across the parameter grid with γ held out, γ being a function of quantal content rather than a free parameter (Supplementary Materials S3.4).

Equation (21) is a relative width—the excursion as a fraction of the measured cleavage. What a blot has to resolve when two serotypes are run on the same platform is the excursion itself, in points of measured cleavage, and the two differ because β moves the operating point. Multiplying through,

(22) $$\Delta \left(\rho \langle f\rangle \right)\;=\;\rho \;h\left(\langle f\rangle \right)\;\frac{W}{\sqrt{N}},\;h\left(x\right)\;=\;\left(1-x\right)\ln\;\left[1/(1-x)\right],$$

in which the pool fraction no longer cancels but is common to the two serotypes measured in one preparation, so the comparison between them is free of it. Carrying β through the model at the fixed threshold coefficient of variation and fixed expected count (Supplementary Table S4), h(〈f〉)W falls from 0.3312 at β=0 to 0.1372 at β=6—the value the alternative reading of the pool fraction returns at SF=2 (Supplementary Materials S1.14 and S1.16)—a ratio of 2.41. At equal *N* and equal assay, BoNT-E’s outcome transition therefore occupies a band of measured cleavage between two and three times as wide as BoNT-A’s for β anywhere between about 4 and 8. The prediction is directional, it follows from the one parameter that represents the molecular difference, and it is evaluated at a value fixed elsewhere in the paper rather than chosen here.

The relative width moves the other way, and we state it because it determines what the prediction is a prediction about. Over the same range, Equation (21) rises from 0.603 to 0.936: as a fraction of the measured cleavage, the informative band is wider for BoNT-A, because its transition sits lower on the cleavage axis. The two readings correspond to two error models for the assay. If between-preparation reproducibility is an absolute error in points of total SNAP-25—which is the case when the two serotypes are compared on one platform and the limiting noise is background and transfer rather than loading—the ordering above holds. If it is a coefficient of variation on the measured value, the ordering reverses. The architecture fixes the magnitudes; which of the two governs a given comparison is a property of the assay and is measurable independently of anything here. We report the absolute reading as primary because it is the one the published comparisons instantiate, and Supplementary Materials S3.2 gives both columns.

This supersedes the derivation our earlier formulation gave, which obtained the same ordering by varying γ from 2–3 to 12 at a fixed operating point. That derivation is withdrawn and recorded in Supplementary Table S8: γ is not free but a function of quantal content, γ=12 is the superseded rodent value and is not attainable at the human reference under any β, and the ordering does not need it.

The consequence for a cleavage–effect comparison is a statement about resolution, not about correlation. At $N=10^{4}$ the band is 0.60% of the measured cleavage for β=0 and 0.94% for β=6; in absolute terms, at the frog–culture pool fraction ρ=0.2277, it is 0.075 and 0.031 points of total SNAP-25 respectively. Ranking preparations by outcome requires an assay reproducible to better than that between preparations, on whichever of the two scales its error is expressed. Densitometric immunoblotting of whole-tissue lysates, in which the pool fraction ρ itself varies between preparations, is not. What is observed is accordingly governed by the between-preparation dispersion of the assay rather than by the model’s mean curve. The architecture does not, however, predict a small cleavage–effect correlation coefficient, nor one falling with *N*: that is the third of the withdrawn claims of Supplementary Table S8, and Supplementary Materials S3.4 sets out why no measurement reinstates it. What the architecture predicts is the width of the informative band and, under the absolute reading of Equation (22), the ordering of that width between the two serotypes; Supplementary Materials S3.3 states what the account does and does not establish.

### 4.6. Sensitivity to the Uptake Count Scale

Supplementary Table S18 confirms the argument of Section 2.2: the relative shift in ${\bar{C}}_{\mathrm{crit}}$ falls monotonically from +150.2% at λ=1 to +5.4% at the reference λ=35 and +0.5% at λ=500, while the probability that a single productive entry suffices falls from unity to below $10^{-40}$. At λ≈1, the large apparent mean-field error is a symptom of the degeneracy, not evidence that uptake stochasticity matters. The condition is properly stated on the expected count at the operating point rather than on λ alone; the mean-field expressions are accurate under E[K]≳10 and degenerate below E[K]≲2 (Supplementary Materials S1.23).

Critically, Proposition 1 is unaffected: the sharpness law depends on $p^{\prime }\left({\bar{C}}_{\mathrm{crit}}\right)$, and both formulations supply a smooth monotone *p*. The constant K shifts with λ; the $N^{-1/2}$ scaling does not.

### 4.7. Recovery of Model Quantities from Aggregate Data

Because the framework is not fitted to empirical data, we assessed how well quantities of interest can be recovered from aggregate outputs. Pseudo-data are binomial responder counts—625 replicates per dose point—simulated from known parameters and fitted by Powell optimisation from eight independent starts on each of twelve pseudo-datasets. Design, objective and classification cutoffs are pre-specified in Supplementary Table S17. Results are in Supplementary Table S17.

The design comparison. PR5 and PR5b differ in exactly one respect. Both use the same generating model, the same binomial noise, the same optimiser, the same protocol and the same random seed; PR5 supplies dose–response curves at five unit counts (N=200 to 10,000), PR5b a single curve. Under the binary readout, the median error on $\sigma_{\theta }$ is 1.87% for PR5 against 4.25% for PR5b, and the interquartile range 5.43% against 10.18%: a twofold separation attributable to the design, since nothing else differs. It does not survive the operating point the rest of this paper uses, and the next paragraph reports that rather than leaving it to be inferred.

That comparison is deliberately narrower than “$\sigma_{\theta }$ becomes identifiable”: it requires no structural result, and it shows that the *N*-dependence predicted by Proposition 1 carries information about threshold dispersion that a mapping containing no *N* cannot supply. It is also, as stated, a property of the binary readout, and we withdraw it in that form. Those figures are obtained under the binary terminal response, which Section 4.3 shows to be inadequate at the human reference. Repeating the identical comparison under the quantal response inflates both errors: PR5 rises to 9.43% and PR5b to 19.53%, moving $\sigma_{\theta }$ from well recovered to poorly recovered at the operating point the rest of this paper uses. This occurs with γ known exactly, so it is a property of the graded response and not of uncertainty in γ. The design advantage narrows with it: 2.27× under the binary reading, 2.07× graded with γ known, and 0.96× on the median once γ carries the uncertainty its quantal derivation permits. What survives is the direction—the *N*-ladder carries information a single curve does not—and not the factor of two.

Two results reported against our own interest. First, ${\bar{C}}_{\mathrm{crit}}$ is recovered to 0.02% even when the fitted model is misspecified (PR3: stochastic-uptake truth fitted with the mean-field reduction). This is expected—${\bar{C}}_{\mathrm{crit}}$ is the median effective concentration and is read almost directly off the observed curve—and it is emphatically not evidence that the architecture is identified. Second, the derived composites of PR2, PR4, PR6 and PR7 are recovered well, but they are functionals of the response rather than parameters, and recovering a functional constrains the underlying parameters only weakly.

### 4.8. Cohort Curves and Between-Subject Dispersion

Unit thresholds are drawn once per muscle per subject, so $P(Y\;=\;1|\bar{C})$ is not a within-subject response probability but the between-subject distribution of the individual critical dose (Supplementary Materials S6.5). The finite-size term is therefore itself a component of between-subject variance, and the question is whether it can be separated from the others.

Proposition 1 concerns one subject. A clinical dose–response curve is a cohort average, and subjects differ in the gain that maps administered dose onto local concentration—muscle mass, injection technique, lot potency, baseline severity. If the individual dose requirement is log-normal with standard deviation $\sigma_{\log}$, the two contributions to the observed relative width add in quadrature,

(23) $$w_{\mathrm{obs}}^{2}\;=\;\underset{\mathrm{finite}\;\mathrm{size}}{\underset{︸}{\frac{W^{2}}{N}}}\;+\;\underset{\mathrm{between}\;\mathrm{subjects}}{\underset{︸}{{\left(2z_{q}\sigma_{\log}\right)}^{2}}},$$

where $w_{\mathrm{obs}}=\Delta D_{q}/D_{50}$.

Equation (23) agrees with the exact convolution of the individual curve against the log-normal gain to within 2% for $\sigma_{\log}\leq 0.5$, the residual being the difference between a relative and a logarithmic width.

Order of magnitude from published dose–ranging data. Three glabellar dose–ranging studies report responder rates at peak effect across at least two dose levels, and Table 5 sets out why none of them can constrain the transition width. Refitting the reported rates by binomial likelihood returns an effective Hill exponent of 1.49 (95% CI [0.49,2.53]) for abobotulinumtoxinA and a lower bound of 2.7 with no upper limit for the male study; the upper end is not identified, because in all three studies, the lowest arm already lies above the median effective dose, so the transition itself was never sampled. Higher exponents can be extracted from the same arms only by adopting a tie-breaking convention for the arms reporting complete response, and are logarithms of that convention rather than measurements (Supplementary Materials S6.6). We stress what this is: two-point fits to aggregate percentages, on wrinkle-severity scales dichotomised into a responder endpoint rather than on the functional block the model describes. It supports an order-of-magnitude statement and nothing finer, and we use it for no more than that.

**Figure 5:**
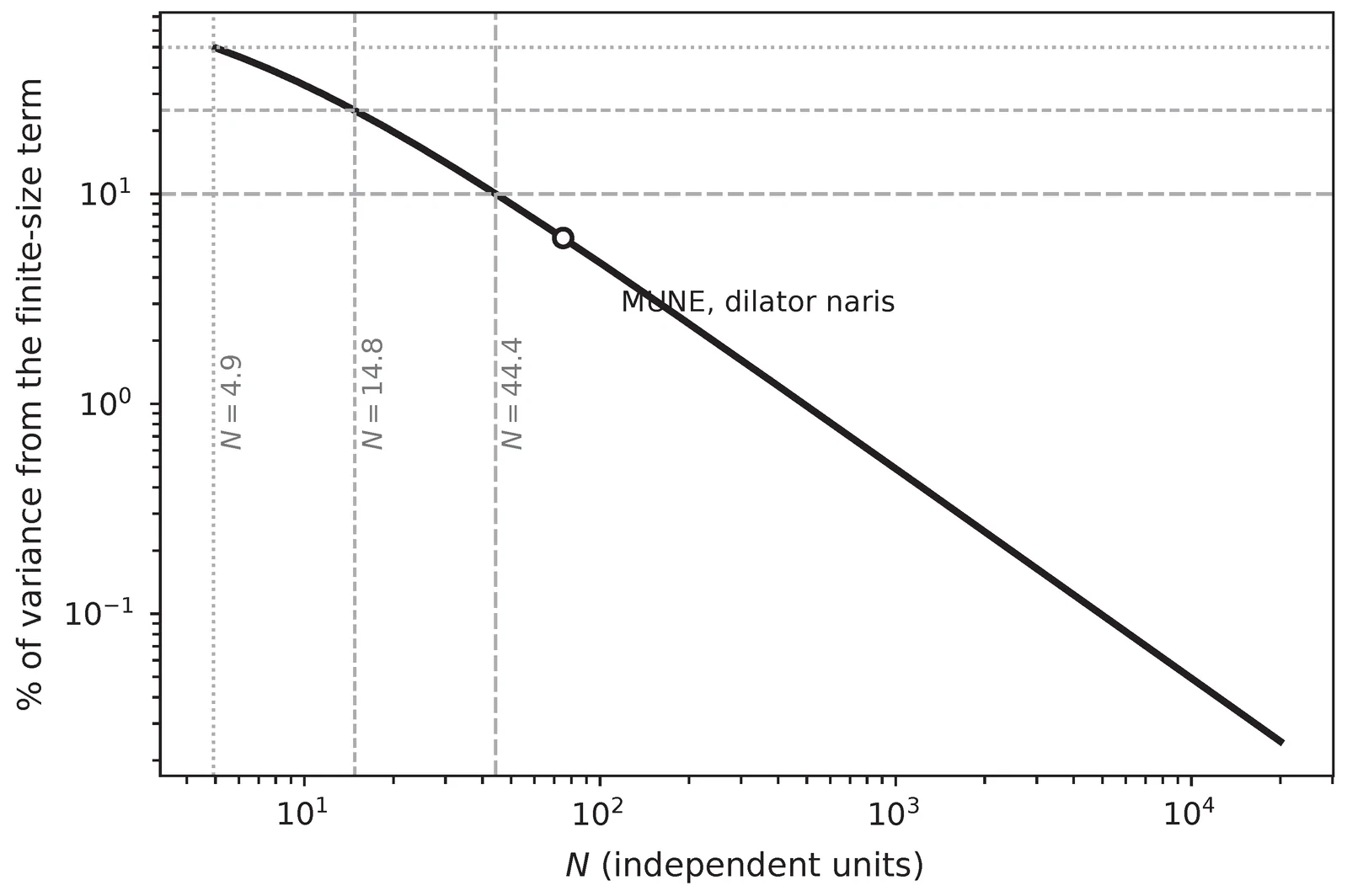
The finite-size term as a correction to classical quantal theory: the share of the observed variance it carries, against unit number, at the externally anchored $\sigma_{\log}=0.308$. The term exceeds a tenth of the variance only below N≈44 and a quarter only below N≈15; at the motor-unit count of a facial target it carries about six percent, and at $N=10^{3}$ about half a percent. The correction is therefore real and measurable in small targets and negligible in large ones, which is why the classical tolerance account is described here as incomplete rather than wrong. Plotted against the observed dose–response curve the two accounts are visually indistinguishable at every target size in this range, which is the same statement read the other way.

The architecture predicts effective Hill exponents of 11.2 at N=20 and 24.1 at N=100 (Supplementary Table S13, exact interquartile ratios), against cohort exponents of order unity. Read naively, the discrepancy looks like a refutation, and Equation (23) shows that it is not. The between-subject term requires a value for $\sigma_{\log}$, and we take it from outside the studies of Table 5 rather than by inverting their exponents: those studies do not sample their own transitions, so any $\sigma_{\log}$ recovered from them inherits that defect. An independent anchor is available. Prescribing information for onabotulinumtoxinA in cervical dystonia reports a mean dose of 236 U with a 25th-to-75th percentile range of 198–300 U in a trial whose patients had extended treatment histories and doses already adjusted individually [54], and a dose interquartile range under individual titration is a direct measure of the dispersion of individual requirement—which is what $\sigma_{\log}$ denotes. Taking that dispersion as log-normal gives $\sigma_{\log}=\ln\left(300/198\right)/\left(2z_{0.75}\right)=0.308$, an anchor whose two qualifications—transport across indication, and a population selected for prior response—pull in opposite directions and are set out in Supplementary Materials S6.6. At that value and W=0.92, the finite-size term carries 19.8% of the observed variance at N=20, 6.2% at $N_{\mathrm{eff}}=75$—the motor-unit count measured in a small facial muscle [52]—and 0.49% at *N* = 1000 (Figure 5). In a parallel-group cohort, the quantity this paper predicts is therefore buried under between-subject dispersion, and no sample size recovers it, because the between-subject term does not shrink with *N*. The consequence is a property of the design and not of the three studies that happen to illustrate it: since the two terms of Equation (23) add in quadrature and only the first carries *N*, a parallel-group dose-ranging trial is uninformative about the transition width whatever its size, and increasing the number of subjects per arm sharpens the estimate of the cohort curve while leaving the finite-size component exactly where it was. What such a design can establish is the median effective dose; what it cannot establish is the steepness, and the distinction matters because the two are conventionally reported from the same experiment.

Why cohort dose–response curves are flat. Two conclusions follow, and the first is the one we did not anticipate.

The model accounts for the flatness of clinical dose–response curves. Cohort exponents of order unity—the refitted 1.49 and the lower bound of 2.7 recovered above from the arms of Table 5—are ordinarily taken to indicate a graded, gently saturating mechanism. Under Equation (23), they are what a near-step individual response looks like once convolved with the anchored between-subject dispersion, $\sigma_{\log}=0.308$, which contributes an interquartile width of $2z_{0.75}\sigma_{\log}=42\%$ of the median effective dose on its own. Shallow cohort curves are therefore not evidence against a threshold architecture; they are what a threshold architecture predicts one should measure in a cohort. This is one of the two results in this paper that require no new measurement—the other is the ceiling of Section 3.2—and it is the one that reinterprets data already collected, inverting the reading those data ordinarily receive. The flatness is not a property of the tissue but of the aggregation. This is a genuine account rather than an accommodation, because the same equation forbids the alternative: a mechanism that were genuinely graded at the level of the individual would give $n_{\mathrm{eff}}$ independent of *N*, whereas Supplementary Equation (S54) requires the residual to fall as 1/N once $\sigma_{\log}$ is controlled. The same decomposition also caps the steepness a cohort curve can display, at $n_{\mathrm{eff}}\leq \ln3/\left(z_{q}\sigma_{\log}\right)$, which is 5.29 at the anchored dispersion: a cohort exponent reported materially above 5 at any target size would contradict Equation (23) directly (Supplementary Materials S6.3).

The design that separates the two components is a longitudinal titration study and not a dose-ranging trial: a hierarchical mixed-effects probit [55,56] over repeated sessions, with subject as a random intercept and a target-specific residual $\sigma_{t}=A_{\sigma }N_{t}^{-b}$, in which the architecture predicts b=1/2 and a mean-only mapping b=0. A parallel-group study cannot carry the test at any realistic sample size, and nothing in the published literature has the shape that can.

The same design carries the discriminating comparison of Section 3.8, and this is worth making explicit because the two enter the model at different places. The width test is a statement about the target-specific residual; the potency test is a statement about the mean, and it is obtained by admitting a fixed effect in $\log N_{t}$ there. Under a fractional collective threshold that coefficient is null, since the required silenced fraction does not depend on the population size; under a fixed count, it is positive and substantial in the proportion that Table 3 reports. No second study is required, and a shift in the mean is the better determined of the two quantities at any given number of subjects, so the comparison that separates the two architectures is not the one the design strains to deliver.

What the potency reading requires in addition is that the dose–concentration gain carry no systematic dependence on target size of its own, and the design addresses that in two steps rather than assuming it away. The gain is a property of the subject and is shared across the targets treated in one session, so its subject-level component is absorbed by the random intercept and cannot enter the fixed effect. Its target-level component—muscle mass, injection volume, depth of deposit—is not absorbed, and is what the first of the four conditions stated below controls, by fixing geometry per target class. A residual trend in the gain across classes after that fixing would be aliased with the coefficient, and we state that as an assumption of the potency reading rather than as a property the protocol secures.

One asymmetry of the comparison belongs here rather than to the theory. The fixed-count family is defined only over a bounded range of target size: below the reserve it is met at zero dose, and above the size fixed by the silenceability ceiling of Section 3.2 it cannot be met at any dose. That range is narrower than the ladder this design calls for, so at the upper end of the ladder the alternative is not merely disfavoured by the data but already excluded by Section 3.2 without them.

Supplementary Materials S6.3 specifies the ladder, the sample size, the power and the three departures that would invalidate it. Four conditions govern its validity and are stated here because they constrain the experiment rather than the analysis: injection geometry fixed across the target classes compared; the unit count measured per subject and per target rather than assigned by class; the endpoint matched across targets, since $\Theta_{\mathrm{sys}}$ is endpoint-dependent; and the ratio between the measured and the effective unit count common across the comparison (Section 6). The estimator also carries a finite-sample bias of the same order as its standard error, so the design must be calibrated by simulation against its own realisation before a point estimate is reported.

**Table 5:** Published glabellar dose-ranging studies, and why they cannot constrain the transition width. The responder rate at the lowest dose tested already exceeds one half in every study, so in each case, the lowest arm lies above the median effective dose and the transition itself was never sampled. Rates are trained-observer responder rates at peak effect (weeks 2–4) for the two onabotulinumtoxinA studies and the composite ≥2-grade rate at week 4 for abobotulinumtoxinA. Arm sizes are n=20 for both onabotulinumtoxinA studies; for abobotulinumtoxinA, the total is 399 across five arms and the per-arm denominators are assumed balanced.

Study	Doses (U)	Rate at Lowest Dose	Arms at 100%	The Authors’ Own Conclusion
OnabotulinumtoxinA, women [57]	10,20,30,40	85%	≥1 of 4	No dose dependence demonstrated between 20, 30 and 40 U
OnabotulinumtoxinA, men [58]	20,40,60,80	65%	3 of 4	Superiority of ≥40 U emphasised rather than an overall dose–response relationship
AbobotulinumtoxinA [59]	50,75,100,125	80%	0 of 4	Dose escalation reported as increasing response

### 4.9. Dynamical Extensions

Two secondary dynamical extensions of the base model capture adaptive phenomena observed clinically. Both are phenomenological, and the complete analysis—the drift law, its fixed points, the compensatory mechanisms that might supply a substrate for it and why none of them settles the question—is in Supplementary Materials S4.

Threshold drift and reduced effective uptake were evaluated as secondary illustrations (Supplementary Figures S3 and S4). Under constant drift $\delta_{0}=0.02$, the minimum effective dose increases monotonically across sessions and reaches the safe-dose ceiling in finite time; under response-dependent drift, the trajectory is steeper. The onset of attenuation, the session $t^{*}$ beyond which no safe dose achieves block, has the closed form $t^{*}=\left(f_{\max}-\theta_{0}\right)/\delta_{0}$ with $f_{\max}=1-e^{-\kappa C_{\max}}$. Among the candidate substrates for such drift, alongside sprouting and SNAP-23 recruitment, is increased quantal content at surviving active terminals, a form of compensatory facilitation; like the other two, it is a candidate and not an established route.

Reduced uptake enters as $\lambda_{\mathrm{eff}}=\lambda \left(1-A\right)$ with A∈[0,1) the fractional reduction in internalisation—an effective phenomenological representation and not a molecular description of antibody neutralisation, since the model does not claim that clinical immunoresistance is literally governed by a single linear reduction of λ. Because the response depends on λ and $\bar{C}$ only through their product, block requires $\bar{C}\left(1-A\right)\geq {\bar{C}}_{\mathrm{crit}}$, giving the closed form

(24) $$A^{*}\left(\bar{C}\right)=1-\frac{{\bar{C}}_{\mathrm{crit}}}{\bar{C}},$$

so $A^{*}=0.167$, 0.333 and 0.600 at $\bar{C}=1.2$, 1.5 and 2.5 times ${\bar{C}}_{\mathrm{crit}}$, which is the identity $A^{*}=1-1/\mathrm{multiple}$ exactly. Equation (24) places $A^{*}$ where the expected silenced fraction meets $\Theta_{\mathrm{sys}}$; defining it instead where the system probability crosses one half moves the reference concentration by $3.8\times 10^{-5}$, that is by 0.005%, and leaves all three values unchanged at the precision reported. Supplementary Figure S4 uses the latter convention.

We note explicitly what this does and does not show. The existence of a ceiling on achievable cleavage is not a property of the discrete architecture: any model with saturating cleavage and a capped dose yields the same bound, and a mean-field model with the same $C_{\max}$ yields the same $A^{*}$. What the discrete architecture adds is the abruptness of the crossing—because output is binary in Φ, P(Y=1) falls from one to zero over a vanishing interval in *A*, whereas the mean-only benchmark crosses smoothly. Both extensions are phenomenological: neither $\delta_{0}$ nor the linear form of $\lambda_{\mathrm{eff}}$ has been quantified experimentally, and they are offered as structural illustrations rather than mechanistic claims [60,61].

### 4.10. Duration Under a Minimal Recovery Law

The published dose-ranging studies separate their arms far more clearly on duration than at peak effect, and Section 2.10 states that the model has nothing to say about it. We therefore asked what the architecture implies once the cleaved fraction is allowed to recover, and we report the answer including the part that does not favour the framework. The extension, the tabulated values and the sensitivity analysis behind everything below are in Supplementary Materials S7.

Let $f_{i}\left(t\right)=f_{\max}\;e^{-t/\tau }$ with τ common across units, so that a unit remains silenced while $f_{i}\left(t\right)\geq \theta_{i}$, and define the duration as $T=\inf\{t:\Phi \left(t\right)<\Theta_{\mathrm{sys}}\}$. The condition is equivalent to $f_{i}\left(0\right)\geq \theta_{i}\;e^{t/\tau }$: time enters only as a rescaling of the threshold, so NΦ(t) remains binomial and the whole apparatus of Proposition 1 applies with *t* in place of $\bar{C}$. Three things follow, and a fourth is withheld.

The architecture is inherited. The product $\left(\Delta T_{q}/T_{50}\right)\sqrt{N}$ converges to 1.4401 with relative error $O(N^{-1})$—residual times *N* constant to 2% across N=20–$10^{4}$, against a 229% excursion for residual times $\sqrt{N}$ (Supplementary Table S25)—which is the same order the closed form achieves on the dose axis (Supplementary Table S10). The recovery timescale τ cancels identically, to 31 units in the last place; but this is a dimensional identity rather than a result, since it holds for any recovery law with a single timescale, and we report it as such.

The dose–duration relation is a consequence, not a separate mechanism. Median duration rises monotonically with dose—by a factor of 4.9 between 1.2 and 4.5 times ${\bar{C}}_{\mathrm{crit}}$—with a local elasticity $d\ln T_{50}/d\ln D=1.08$ at the operating point (Supplementary Table S26). Nothing is postulated: starting further above the threshold takes longer to fall back below it. Two details belong with it. The absolute interquartile width of the duration is nearly flat across that range, so the contraction of the relative width is the median growing rather than the dispersion shrinking. And at ${\bar{C}}_{\mathrm{crit}}$ exactly, the lower quartile of duration does not exist, because half the population is already below $\Theta_{\mathrm{sys}}$ at peak—which is what a median effective dose means, read on the time axis.

The decomposition of Equation (23) transports, in two terms and not in three. Writing the observed relative width of duration as finite-size plus between-subject reproduces exact computation to within 3.20%, vanishing in *N*. Resolving the between-subject term further into the dispersion of the recovery timescale and the propagated dispersion of the dose gain does not work, missing by −6.9% to +5.5%: the gain does not propagate log-normally onto logT, its local elasticity running from 1.73 to 0.71 across the quartiles of the gain itself. We report the form that holds rather than adjusting the coefficients of the one that does not.

What is withheld, and why. The value of the constant is not a property of the architecture. Replacing exponential recovery with a linear or a sigmoidal law at matched first moment moves it from 1.440 to 1.178 to 0.807, an excursion of 44%; the share of observed variance carried by the finite-size term moves by 51%, because at small shares it scales as the square. The contrast with the dose axis is the point: there, changing the threshold law across three families at matched moments moves *W* by 0.25% (Supplementary Materials S1.15), because Poisson uptake convolves the law before the collective integration, and no such convolution acts on the time axis. The recovery law in human facial muscle is not measured, so we report the structure and not the constant.

A negative finding. We had expected the duration endpoint to be the more accessible test, since time to relapse is recorded in every registration trial while the dose–response transition is not (Section 4.8). It is not. At the between-subject dispersion recoverable from the published series, the finite-size share on the duration axis stands between 2.7% and 9.7% at $N_{\mathrm{eff}}=75$ against 6.2% on the dose axis (Supplementary Table S27), and the ratio between the two axes spans 0.22 to 1.92 across recovery forms, attributions and target size. The dominant uncertainty is not the model but a separation the data do not permit: the dispersion of the recovery timescale and the propagated dispersion of the dose gain are not identifiable from aggregate relapse figures. What can be said is bounded and is worth saying: the finite-size term does not vanish on this axis either, remaining above 5% of the observed variance up to N≈39 under the most conservative attribution and up to N≈157 under the most favourable, so duration is measurable in a cohort in small targets and not in large ones—the same conclusion the dose axis reaches, by the same mechanism, and to no better resolution.

### 4.11. The Human Reference, and What Fixes It

Setting SF=2 with $N_{S}=1$ and β=0 in Supplementary Equation (S21) gives θ=0.50: this is the value the present work adopts, and the rodent figure θ=0.70 is retained only for comparison with the earlier parameterisation. Holding CV(θ)=0.15 fixed gives $\sigma_{\theta }=0.075$, which we adopt; the alternative transport of CV(SF) is excluded by the model itself and the argument yields a prediction in its own right, that the model requires CV(SF)≲0.15 in human muscle (Supplementary Materials S1.23). What the reparameterisation moves is every constant of scale and no exponent.

Equation (19) at ${\bar{m}}_{0}=25$ then gives $\gamma_{q}=5.64$ against an exact Poisson value of 5.93, and the count scale is rescaled with the operating point as in Supplementary Materials S1.16: λ=35 restores E[K]=14.3, matching the earlier configuration and keeping the model outside the degenerate corner of Section 4.6.

## 5. Discussion

### 5.1. What the Model Establishes

What the paper advances on its own account is the distinction in Section 3.8: the collective threshold is a fraction of the unit population and not a fixed count, so the median effective dose is invariant to target size while the absolute transition width contracts, where the fixed-count and extreme-threshold families require the opposite in both quantities. One consequence of it needs no measurement whatever, and Section 3.2 establishes it from the silenceability ceiling together with the range of target sizes over which the toxin is used clinically.

Read without qualification, the invariance of potency to target size appears to be contradicted by every dosing table in use: a large muscle is treated with more units than a small one, and always has been. The two statements are not in conflict, and the distinction between them is the one the discriminating experiment has to respect. What the architecture holds fixed is the fraction of units that must be silenced, not the amount of product required to silence them. The administered dose reaches the target as a local concentration through a target-specific gain, and that gain falls with muscle mass, with the volume over which the same dose is distributed, and with the distance the toxin must travel from the depot; larger targets therefore take larger doses for reasons that lie entirely in the gain and not in the required fraction. The claim is the narrower one that once the gain is held fixed, target size leaves the location of the curve and enters only its width. Read in the other direction, the same decomposition separates two quantities that dosing practice necessarily reports together: the fraction of units a given endpoint requires, which this architecture holds constant across targets, and the target-specific gain, which carries the whole of the observed variation in administered units and which the model neither predicts nor constrains. That is also why the comparison must be made within subject and with injection geometry held constant across the targets compared (Section 4.8): a design that allows the gain to vary systematically with target size will reproduce the fixed-count prediction from a fractional mechanism, and will do so whatever the mechanism actually is.

The instrument that carries the distinction is Equation (12), verified to better than 0.5% against exact binomial computation over more than two decades in *N* (Supplementary Table S12). The relation itself is prior (Section 3.11); what it supplies here is that it is a constraint and not a capability: it forbids rather than permits, fixing the transition on a target of different unit number once a mapping is calibrated on one (Supplementary Materials S6.7).

The geometry result is secondary but clinically suggestive. Because block requires the dispersion radius to exceed $s^{*}$, and because $s^{*}$ falls only sublinearly with dose, spatial distribution and total units are not interchangeable within the model. The architecture identifies the lever as volume rather than site count, and separates the two channels through which dilution acts (Supplementary Materials S6.1–S6.5).

### 5.2. Clinical Reading

The clinical content of the sharpness law is one statement, and it can be read without any of the machinery above: the smaller the target, the less repeatable the outcome of the same dose, and the difference is not marginal. A target containing a few tens of independent units carries several times the outcome dispersion, at a nominally identical dose, of one containing a thousand, for the arithmetic reason that an average taken over fewer elements fluctuates more. That dispersion is between patients and between targets and not within one patient across sessions, since thresholds are drawn once per muscle per subject (Section 4.8). The counts below are independent-unit counts in the sense of Section 2, one to three orders of magnitude smaller than any anatomical count, so small mimetic muscles sit at the bottom of the range rather than outside it.

On the operative scale of Section 4.1, the width of the outcome-uncertainty band spans roughly 49% of the critical dose at N=20, 22% at N=100 and 7.1% at *N* = 1000 (Supplementary Table S13). If the effective independent-unit count in small perioral muscles is at the low end of this range, then outcome variability at nominally identical doses should concentrate in exactly those targets where placement is most difficult—a pattern consistent with clinical experience, and recorded in Table 1 as a consistency account rather than as evidence.

The ordering, however, is not a consistency account but a consequence of the exponent, and it holds whether or not the test of Section 3.12 is ever performed: outcome is intrinsically less predictable in small targets than in large ones, by the sevenfold factor above, because they contain fewer independent units and for no other reason. No refinement of placement, dilution or product removes it. What it argues for is smaller dose increments and a lower threshold for staged titration in small muscles, and against transferring a dosing convention from a large target to a small one.

We do not claim that the framework explains secondary non-response. The drift extension shows that a monotone upward threshold shift produces finite-time escape from the therapeutic window, but the drift rate is unmeasured and the compensatory mechanisms invoked—sprouting and reinnervation [62], SNAP-23 recruitment [63,64], compensatory facilitation—are not established as cumulative across sessions (Supplementary Materials S6.7).

### 5.3. An Account of a Standing Observation

The architecture reframes an observation usually presented as a paradox. That a small measured fraction of cleaved SNAP-25 abolishes transmission, and correlates poorly with paralysis, is normally attributed entirely to dominant-negative potency. The present analysis separates two contributions that attribution conflates: a measurement effect (Equation (6)) and an architectural one (Equation (21)). Neither replaces the dominant-negative mechanism, for which there is direct molecular evidence [23]; both reduce how much work it has to do.

The serotype asymmetry is already in the literature, and the account of it given here is a consistency account in the sense of Table 1: it was not collected to test this model, and we have not fitted it. It is nonetheless directional rather than merely compatible, since the ordering follows from the one parameter that represents the molecular difference, evaluated at a value fixed elsewhere in the paper, and it carries a stated condition on the assay error model under which it would reverse (Section 4.5). What discriminates the architectures is the comparison of potency across target sizes in Section 3.8, and not this observation.

### 5.4. Empirical Tests

The falsifiable content of this paper sits at three levels of experimental cost, and the two cheapest require no new data at all.

Level 1—Already done. Two consequences of the architecture are already established above and require nothing further of an investigator. The silenceability ceiling of Section 3.2 excludes the fixed-count family, by the argument given there. And Section 4.8 shows that the shallow cohort curves the literature reports are what Equation (23) predicts rather than what it contradicts. What distinguishes these two from an accommodation is that each forbids something: the first, a family of architectures; the second, the genuinely graded alternative, which would give an effective exponent independent of target size.

Level 2—One bench measurement. The terminal pool fraction ρ of Section 2.5 is in principle measurable by subcellular fractionation of neuronal SNAP-25, and a measured value near unity would refute the present parameterisation outright (Equation (7)). The measurement is standard, requires no new animal model, and the two anchors in that section—frog neuromuscular junction and rat gastrocnemius in vivo—already bound ρ to a factor of two from published lysate data. A direct measurement settles which anchor is appropriate and removes the largest single conditional from Table 2.

Level 3—Longitudinal human titration. The complete discriminating design is a longitudinal titration across facial targets spanning fiftyfold in independent-unit number, with motor-unit number estimation per subject and per target and eight sessions of titration (Supplementary Materials S6.3). It requires 160 subjects to keep the standard error on *b* below 0.10 and is feasible within normal upper-face practice, since patients return at three- to four-month intervals over years; the single measurement it adds to ordinary practice is motor-unit number estimation, an established electrophysiological technique with normative data that has been applied to a facial target directly [52]. No calibration of local concentration, no lot-potency assay and no cleavage measurement enters at any point. Two limits of it should be stated plainly. It establishes that unit number sets the transition width, with power above 0.75 against the mean-only hypothesis in every estimable cell; it does not measure the exponent, its power to separate b=1/2 from b=1/3 never exceeding 0.35 and falling to 0.03 at the fiftyfold ladder the design itself recommends. A programme funded to measure the exponent would be funded for the wrong endpoint. Levels 1 and 2 can be completed first and will substantially narrow what Level 3 still needs to establish.

### 5.5. What Would Falsify the Exponent, and Not Merely the Constants

Table 2 lists what a future measurement would update by substitution. It is equally important to say what it would not. The exponent −1/2 is not a metaphysical commitment: it follows from the central limit theorem applied to units that are exchangeable with short-range correlation and read out through a threshold fixed as a fraction. Four structural discoveries would break it rather than reparameterise it, and we list them so that the perimeter is declared rather than implied.

(i) Ordered recruitment, under which the weights of Supplementary Materials S1 would not be independent of the unit states. We know neither the sign nor the size of this effect.
(ii) A collective threshold that depends on *N*, or on which units fail rather than how many. $\Theta_{\mathrm{sys}}$ is a fraction by construction here, and no measurement constrains that choice. A merely graded readout does not break the exponent (Section 2.7); a readout whose gradedness scales with *N* would.
(iii) Long-range correlation whose correlation length scales with target size, which would make $N_{\mathrm{eff}}$ a function of *N* and move the exponent rather than the prefactor. The territory bound of Supplementary Materials S1, Supplementary Materials S1.8 excludes this only for territories of fixed extent.
(iv) Compensation acting within the measurement window, which would make Φ non-stationary during readout and invalidate the binomial step itself.

None of these is excluded by anything in this paper. Each is a structural claim rather than a parameter value, and each would require a different model rather than different numbers.

## 6. Limitations

No empirical calibration. The framework has not been fitted to data. All numerical values are internal to the model and should be read as structural indicators, not calibrated predictions. Vesicle pool dynamics, synaptic facilitation, tissue diffusion kinetics and the regulatory pathways governing NMJ maintenance are not represented.

The independent unit is the motor unit, not the terminal. This is the most consequential limitation, and it bears directly on the main result. Equation (12) assumes independent silencing, whereas terminals belonging to one motor unit share the neuronal determinants of their threshold and are therefore strongly correlated. The entity modelled throughout is the terminal, which is what has a position in the field, a local concentration and a threshold; the motor unit enters as a correlation structure over the thresholds of sibling terminals, giving $N_{\mathrm{eff}}=N_{\mathrm{fib}}/\left[1+\left(m_{f}-1\right)r_{S}\right]$ with $r_{S}$ attenuated below the threshold correlation, so that dividing by the innervation ratio is the perfectly correlated corner rather than the general case (Supplementary Materials S1.8). Exposure sharing within a territory acts through the same channel and is bounded there. Two consequences follow. All absolute widths reported here are underestimates, by a factor of three to ten, which strengthens rather than weakens the clinical reading. And second, the law becomes more testable rather than less: motor-unit number estimation is an established technique with normative data [65,66] and has been applied to facial muscles directly [52], whereas terminal counts in human facial muscles are not measurable at all. The technique measures $N_{\mathrm{MU}}$ and the law requires $N_{\mathrm{eff}}$, whose ratio $m_{f}/\left[1+\left(m_{f}-1\right)r_{S}\right]$ is unity only at perfect within-unit correlation and is 2.5 at $r_{S}=0.33$, $m_{f}=10$. A common ratio across the targets compared is absorbed into the constant and leaves the collapse intact; a differing one does not, which is one of the conditions the design of Section 4.8 states. And those normative sets show the several-fold between-muscle variation that Prediction 1 exploits. The three qualifications compound rather than stand side by side, and the compound is this: the quantity in which the law is written has been estimated in one facial muscle so far, with a published test–retest coefficient of variation of 29.6% that bounds how finely *N* can be graded across targets, and only up to a multiplicative ratio that is not itself measured. None of the three is fatal to the design of Section 4.8, and each is neutralised in a different way—the ratio cancels when it is common across the compared targets, measurement error in $N_{t}$ attenuates the estimate towards the mean-only hypothesis and therefore cannot manufacture a positive finding, and *N* is specified to be measured per subject and target rather than assigned by class. That reassurance covers measurement error and does not cover the ratio, and we separate the two rather than let the first stand for both. A ratio varying randomly across the classes compared behaves as further measurement error and attenuates in the same direction. A ratio varying systematically with target size does not: because the ratio rises with the number of fibres per motor unit, and larger muscles have more of them, regressing the target-specific residual on the measured motor-unit count rather than on the effective count returns an exponent inflated by the elasticity of the ratio—that is, biased towards the architecture rather than away from it. Two things bound the damage. The ratio is confined between unity and the reciprocal of the within-unit correlation, so its logarithm can move only by a fixed amount however wide the ladder, and the elasticity therefore falls as the ladder is widened rather than rising with it; over the ranges of Supplementary Materials S1.8 the inflation is smaller than the finite-sample bias of Supplementary Materials S6.3 and acts against it. And the contaminating factor multiplies the exponent, so under the mean-only hypothesis, where the residual does not depend on the effective count at all, no trend in the ratio can generate a dependence on the measured one. The departure inflates a true positive and cannot fabricate one. It is a further reason—independent of the finite-sample bias—why the design is specified to establish that unit number governs the width and not to measure the exponent. But a design requiring *N* to be known in absolute terms could not be run today, and none is proposed here. We report results in terms of *N* throughout and state explicitly that *N* is to be instantiated as an independent-unit count.

The independence assumption is contested in the exact system on which the model is instantiated, and we state the objection rather than leaving it to a reader. Direct simulation of motor-unit populations indicates that force variability is not principally attributable to motor-unit noise: recruitment order, common synaptic drive, and nonlinear muscle mechanics dominate, so a muscle is not well described as a population of independent binary units contributing additively [67]. The same conclusion is reached from the opposite direction in sensory populations, where information scales sublinearly with population size once correlated noise is present, and the $\sqrt{N}$ gain of an independent population is recovered only under conditional independence [68].

Neither result invalidates the exponent, and neither is dismissed. The quantity entering Proposition 1 is already an effective independent-unit count and not an anatomical one, and Supplementary Materials S1 derives the reduction that within-unit threshold correlation, unequal weighting and shared exposure jointly produce. What refs. [67,68] establish is that the reduction is large rather than marginal, and that its size is not known for human facial muscle. They also identify the one departure that would break the exponent rather than rescale the count: ordered recruitment makes the unit weights depend on the unit states, which the effective-count argument assumes they do not (Section 5.5). We list it there among the structural claims that would require a different model rather than different constants.

Units are weighted equally, and they are not; independent weighting reduces *N* by a bounded factor without altering the exponent (Supplementary Materials S1.7).

The uptake count scale is assumed, not measured. The reference λ=35 is a declared assumption, fixed by the requirement that E[K] sit at the same operating point as in the earlier parameterisation. Section 4.6 reports the full dependence: conclusions are stable for an expected count $E\left[K\right]=\lambda {\bar{C}}_{\mathrm{crit}}≳10$ at the operating point, and the model degenerates into a Poisson entry gate for E[K]≲2. We do not know the true value; we know what changes with it.

Parameter degeneracy persists. In the mean-field reduction, the response depends on κ=λη and $\bar{C}$ only through their product, so λ and η are not separately identifiable from dose–response data; retaining uptake stochasticity breaks the exact symmetry but leaves a strong practical ridge, and threshold dispersion is poorly recovered under the graded readout the human reference requires. Section 4.7 reports both with their figures. What we stress here is only what those figures are: recovery performance under one design and one noise model, and not a statement about identifiability in general (Supplementary Table S17).

The binary terminal response is not adequate, and the correction is reported rather than the idealisation: all binary-limit widths are lower bounds, and Section 4.3 carries the constants. The steepness that replaces it is derived but not sharp, because quantal content is measured only to a range and the derivation is a step function of it (Supplementary Materials S1.20). The scale-free width is therefore 0.92 to two significant figures and not four. What the sharpness law asserts is that $n_{\mathrm{eff}}/\sqrt{N}$ is constant across targets, not that it takes a particular value, and the constancy is what the test of Section 3.12 interrogates. The $N^{-1/2}$ scaling is unaffected throughout, verified numerically rather than assumed.

The mapping to cleavage assays rests on an unmeasured quantity, and none of its anchors is human. The terminal pool fraction ρ of Equation (6) has not, to our knowledge, been measured; it is bounded to [0.23,0.47] by anchors from frog, rodent culture, mouse hemidiaphragm and rat muscle in vivo (Supplementary Materials S1.16), a factor of two set by which preparation supplies the anchor. Every absolute concentration here is conditional on that value, and if ρ is near unity, all of them rescale. Because the sharpness law constrains an exponent and a ratio rather than an absolute dose, its content survives either resolution—but the numbers do not, and we would rather say so than present conditional values as settled. Safety factors are lower in humans than in rodents [27,69], which Section 4.11 carries through; that absolute figures transfer to human facial muscle remains an assumption we make explicitly.

The model is static, and the serotype comparison is where that bites. The observations underlying Section 4.5 are cross-sectional in time, while the two serotypes differ by orders of magnitude in intracellular persistence, so part of the reported decorrelation may be a sampling-time artefact rather than an architectural effect. Section 2.10 states what survives this and what does not.

The dynamical extensions are phenomenological. Neither the drift rule nor the linear uptake-impairment form is derived from measured kinetics.

## 7. Conclusions

A discrete threshold model of BoNT-A action yields four statements that stand on their own.

Published cleavage assays measure a different quantity than the model’s. Densitometry of whole-tissue lysates has as its denominator all neuronal SNAP-25, terminal and non-terminal alike, so the measured figure is ρ times the releasable-pool fraction the mechanism depends on. The model bounds ρ to [0.23,0.47] from published anchors, and a measured value near unity would refute that parameterisation while leaving the structural results intact. Part of the standing puzzle that a small measured cleavage fraction abolishes transmission is therefore a denominator effect, and is separable from the dominant-negative mechanism by subcellular fractionation.

The collective threshold is a fraction of the unit population and not a fixed count. Fixed-count and extreme-threshold architectures require potency to move with target size and the absolute transition width to widen with it; a fractional threshold requires potency to stay fixed and the width to contract. The two are distinguishable from responder rates against administered dose. And one consequence requires no experiment: the silenceability ceiling makes every fixed collective count fail above a target size it determines, and the toxin works across target sizes spanning orders of magnitude.

Outcome is intrinsically less predictable in small targets. The finite-size band spans some 49% of the critical dose at N=20 against 7% at *N* = 1000. The ordering follows from the exponent alone and no refinement of placement, dilution or product removes it. It argues for smaller dose increments and staged titration in small muscles, and against transferring a dosing convention from a large target to a small one.

Shallow clinical dose–response curves are what a threshold architecture predicts. A near-step individual response convolved with the between-subject dispersion of individual dose requirement returns a cohort curve of effective Hill exponent of order unity, which is what the published glabellar studies report. Flatness is therefore not evidence against a threshold mechanism; the same decomposition caps the exponent a cohort curve can display and excludes the genuinely graded alternative, which would give an exponent independent of target size. This statement reinterprets data already collected and requires no new measurement.

Four further findings are negative and we report them as such. A duration endpoint is not more accessible than a dose endpoint: a minimal recovery law shows that duration inherits the same architecture and the same exponent, but the constant moves by 44% across recovery laws that no measurement distinguishes, and the share of cohort variance the finite-size term carries is not determined within a factor of two. Published glabellar dose-ranging studies cannot constrain the transition width under any architecture: in all three, the responder rate at the lowest dose already exceeds one half, and the higher exponents obtainable from them are logarithms of a tie-breaking convention for the saturated arms rather than measurements. Under the graded terminal response the model requires, threshold dispersion is poorly recovered from aggregate data and the design advantage of a multi-target ladder is not demonstrated. And four statements advanced in earlier formulations do not survive: three claims, judged against a stochastic mean-only benchmark, and one derivation, whose conclusion is retained but whose route is not.

The architecture is not specific to botulinum toxin. It requires a finite population of units failing independently, a per-unit threshold with dispersion, and a collective readout on the silenced fraction; the pharmacology enters in fixing the constants and in making them measurable. What the instantiation adds is that the constants can be anchored to the safety factor and quantal content of neuromuscular transmission, and that the unit count is accessible by motor-unit number estimation.

## 8. Computational Methods

Reported quantities are computed analytically; Monte Carlo simulation is used to verify them and never as the source of a reported analytical value. Two blocks are simulation studies by construction and are the exceptions: the synthetic recovery of Section 4.7 and the design simulation of Supplementary Materials S6.3, whose figures are Monte Carlo estimates and are reported and qualified as such. These verification runs instantiate the same generative assumptions as the analytical derivations and therefore do not constitute external empirical validation [70]. The complete annotated source code, environment specification, production implementation details, and analytical verification of all the tabulated results are provided in Supplementary Materials S5.

### 8.1. Computational Environment

Simulations were implemented in Python 3.9.6 using NumPy 2.0.2 for array operations, with pseudo-random numbers from the PCG64 generator [71]; SciPy 1.13.1 was used for analytical verification of the tabulated results and is not a dependency of the simulation engine itself. Seeds are fixed per experiment rather than globally, and all results are exactly reproducible given identical library versions. The reference condition used κ=2.0, λ=35, $\mu_{\theta }=0.50$, $\Theta_{\mathrm{sys}}=0.60$ and $\sigma_{\theta }/\mu_{\theta }=0.15$ (Supplementary Table S7), the last carried over from the earlier parameterisation as a declared assumption rather than a measurement (Section 4.11). The stochastic-uptake formulation is the primary model throughout and the mean-field reduction is retained as the analytically tractable reference; the two differ by 5.4% in ${\bar{C}}_{\mathrm{crit}}$ at the reference count scale, and standalone mean-field values should not be read as calibrated predictions. The numerical architecture, the two continuous benchmarks, the synthetic-recovery design and the convergence checks are set out in Supplementary Materials S5.1–S5.3.

### 8.2. Use of Generative AI

A generative AI assistant (Claude Opus 4.6, Anthropic) was used for algebraic derivation, checking of the analytical results in Section 3 and Supplementary Materials S2 and for independent numerical verification. The model was specified by the authors, and the analysis code released with this work was written, corrected and executed by them; every reported number was regenerated from that code, and every analytical result was independently checked. The assistant is not an author, and responsibility for the correctness of all content rests entirely with the authors.

### 8.3. Synthetic Recovery: Design

Recovery experiments simulate aggregate observables from known parameters and attempt to recover a target quantity from them. Recovery performance is not identifiability, and the two are separated in Supplementary Materials S5.8, which also settles the structural question for κ: at achievable noise, every value between 0.80 and 4.54—a factor of 5.7—remains compatible with the data once the threshold parameters are re-optimised, which is why the median error of PR1 is large. Four design choices determine what the results mean and are stated in full in Supplementary Materials S5.6: observables are binomial responder counts at 625, which replicate per dose point rather than clipped Gaussian perturbations; the dose–response curve, the multi-*N* ladder, the geometry grid and the uptake-modulation boundary are separate blocks, with no output-variance block, since Var(Y) carries no information the mean curve does not; candidates are evaluated at the physical concentrations used to generate the data; and Powell’s method is used, because the objective is piecewise constant in $\Theta_{\mathrm{sys}}$. Failed starts are recorded as failures and never replaced by fallback values.

### 8.4. Reproducibility and Convergence

Because reported values are analytic, convergence is a property of the verification runs rather than of the results. Monte Carlo agreement was checked at $\bar{C}={\bar{C}}_{\mathrm{crit}}$, where the estimator variance is maximal and the check is therefore hardest: standard errors follow the expected $n^{-1/2}$ decay and every 95% interval covers the analytic value (Supplementary Table S16). Away from the critical point, the system response saturates to 0 or 1 within a few percent of ${\bar{C}}_{\mathrm{crit}}$ (Supplementary Table S15), so verification there is uninformative and is reported only for completeness.

## Figures and Tables

**Table 1:** The status of each result. Analytical results are proved in Supplementary Materials S2. Computed results follow from the analytical ones by exact evaluation, with the numerical method stated. Consistency accounts reproduce a published qualitative pattern without being fitted to it and are not evidence; where such an account also excludes the standard alternative reading of the same data it is marked as such, and it then constrains rather than merely accommodates. Phenomenological constructions are chosen for parsimony and are not derived from measured kinetics. Two entries carry a status that the other results do not. A withheld quantity is one the model computes but whose value depends on a modelling choice no measurement constrains, so that reporting it would attribute to the architecture what belongs to the choice; unlike a conditional quantity of Table 2, no substitution of a measured parameter recovers it. A negative entry records a question we asked and answered in the direction that does not favour the framework.

Result	Status	Where
Silenceability ceiling $\phi_{\max}<1$, excluding fixed-count collective thresholds above a target size	analytical	Section 3.2, Equation (11)
Sharpness law $\Delta {\bar{C}}_{q}\sqrt{N}=K$	analytical	Proposition 1
Error order $O(N^{-1})$ and Berry–Esseen bound	analytical	Supplementary Equation (S45)
Scale-free form *W*, invariance to dose gain	analytical	Section 3.7
Effective Hill exponent $n_{\mathrm{eff}}\propto \sqrt{N}$; coefficient conditional and not discriminating	analytical	Equation (15), Section 3.8
Non-transportability of a calibrated mapping	analytical	Supplementary Materials S2
Geometry non-sufficiency at matched mean	analytical	Proposition 2
Between-subject decomposition of the width	analytical	Equation (23)
Duration under a minimal recovery law: same architecture, same exponent; the cancellation of the recovery timescale is a dimensional identity and not a result	analytical	Section 4.10
Two-term separation of Equation (23) transporting to the duration axis	computed (exact inversion)	Section 4.10
Constant of the duration relation	withheld: conditional on the functional form of recovery, which moves it by 44%	Section 4.10
Relative accessibility of a duration endpoint against a dose endpoint	negative: not determined within a factor of two	Section 4.10
Critical dispersion radius $s^{*}$	computed (Edgeworth)	Section 4.2
Fractionation versus dose escalation	computed (Edgeworth)	Supplementary Table S24
Response steepness $\gamma_{q}$ from quantal fluctuation	computed	Section 4.3
Pool fraction ρ∈[0.23,0.47]	computed, falsifiable	Equation (7)
Recovery of $\sigma_{\theta }$ from an *N*-ladder	negative under the graded readout: the twofold design advantage is a property of the binary limit and is withdrawn in that form	Supplementary Table S17
Invariance of $D_{50}$ to unit number	computed (exact inversion), discriminating	Section 3.8
Serotype asymmetry in cleavage–effect correlation	consistency account, conditional on the assay error model	Section 4.5, Equations (21) and (22)
Flatness of published cohort dose–response curves	consistency account, graded alternative excluded	Section 4.8
Longitudinal threshold drift	phenomenological	Section 4.9
Effective uptake modulation	phenomenological	Section 4.9

**Table 2:** Conditional quantities: what each unmeasured parameter feeds, and by how much the measurable scale-free width *W* moves across the range, are assessed. Every row is a map from a parameter to an observable, evaluated here at a declared value and re-evaluated at a measured one, so that a future measurement updates this paper by substitution rather than by rederivation. Excursions are one-factor-at-a-time from the human reading of Section 4.11; the last column is the first-order Sobol index from sampling the same ranges jointly, and it reorders the table, since $\sigma_{\theta }$ alone carries half the variance of *W* while λ, second by excursion, is sixth by variance contribution. The exponent −1/2 appears in no row: it is the only claim not conditional on this table, and Section 5.5 states separately what would falsify it. How each range was chosen, why three of them are one-sided or narrower than the sweep behind Supplementary Figure S1, why the composite enters as η=κ/λ, and which entries were not sampled jointly, are set out in Supplementary Materials S5.9.

Parameter	Enters Through	Range Assessed	Excursion of *W*	$S_{1}$
$\sigma_{\theta }$ threshold dispersion	$f_{\theta }\left(f^{*}\right)$	0.075–0.175	35.3%	0.502
λ uptake count scale	CV(K)	10–100	34.9%	0.016
η=κ/λ per-event yield	lattice step vs. $\sigma_{\theta }$	0.029–0.114	17.8%	0.021
β dominant-negative strength	$\mu_{\theta }$ and γ jointly	0–15	13.2%	—
γ response steepness	$p_{\gamma }^{\prime }\left({\bar{C}}_{\mathrm{crit}}\right)$	5.7–6.9 (${\bar{m}}_{0}=20$–30)	10.8%	0.092
*k* endplates per fibre	CV(K), Equation (9)	1–5	10.0%	0.012
$\Theta_{\mathrm{sys}}$ collective threshold	K and ${\bar{C}}_{\mathrm{crit}}$, cancelling	0.50–0.70	7.39%	0.090
$\mu_{\theta }$ mean threshold	$f^{*}$	0.40–0.60	6.9%	0.100
κ uptake–cleavage composite	cancels in *W*	1.0–4.0	0%	0.001
ρ terminal pool fraction	absolute concentrations only	0.23–0.47	0%	—

**Table 3:** The three architectures compared on the same per-unit response, at the human reference under the quantal terminal response of Section 4.3. The fixed reserve is set at c=21 so that the first two coincide at N=50. Quantiles are exact binomial and Poisson inversions, not simulated; values by target size are in Supplementary Table S22. The fixed-count family is defined only over the range shown: below N=21 the requirement is met at zero dose, and above a target size of order a thousand units it exceeds the attainable silenced fraction of Section 3.2 and cannot be met at any dose; that threshold is itself parameter-dependent and is bounded rather than located in Section 3.2. The multi-hit column is not a statement about target size. Its parameter *n* is the number of hits required at a single target and is a property of the molecular target rather than of the muscle, so a multi-hit account instantiated on a muscle would hold *n* fixed and would predict potency invariant to target size as this architecture does. The comparison against multi-hit is therefore carried by the absolute width alone—not by the relative width, which contracts at the same rate under both, and not by the coefficient of Equation (15), which does not discriminate (Supplementary Materials S6.6)—and the comparison on potency is against the fixed-count family alone.

Architecture	Fraction Required for Block	$D_{50}$ Across the Range	Slope of Absolute Width
Fractional threshold (this model)	$\Theta_{\mathrm{sys}}$, independent of *N*	1.036× (N=20–1000)	−0.4887
Fixed count, critical subset [40]	(N−c+1)/N, rising with *N*	2.867× (N=50–500)	+0.6149
Multi-hit target theory [50,51]	P(Poisson(λ)≥n)	50.8× over *n*; see note	+0.5020

**Table 4:** What an experimenter measures across the transition, under the assay mapping of Equation (6) at the human reference (${\bar{C}}_{\mathrm{crit}}=0.4096$, 〈f〉=0.5490, hence ρ=0.125/〈f〉=0.2277 at the frog–culture anchor), $N=10^{4}$. Across the tabulated range, the cleavage assay varies by three percentage points, while the outcome moves from certain failure to certain block; across the two rows between which the outcome actually moves, it varies by less than half a point. The consequence is not that the two are uncorrelated—they are not, since both are monotone in dose—but that the outcome transition occupies a band of measured cleavage narrower than any realistic assay can resolve; Equations (21) and (22) quantify that band, in relative and in absolute units, respectively.

$\bar{C}/{\bar{C}}_{\mathrm{crit}}$	$\bar{C}$	Measured Cleavage ρ〈f〉	E[Φ]	P(Y=1)
0.85	0.3482	0.112	0.481	0.000
0.92	0.3768	0.118	0.539	0.000
0.96	0.3932	0.122	0.570	0.000
1.00	0.4096	0.125	0.600	0.504
1.04	0.4260	0.128	0.628	1.000
1.10	0.4506	0.133	0.666	1.000
1.25	0.5120	0.144	0.746	1.000

## Data Availability

The data presented in this study are openly available in Zenodo at https://doi.org/10.5281/zenodo.22280032. No new empirical data were created or analysed in this study; all results derive from analytic computation, with Monte Carlo used for verification. The repository contains the complete simulation code, the derived outputs and the generated figures, released under the MIT License. All stochastic components use the PCG64 generator with fixed, explicitly recorded seeds—12345 for the recovery experiments, 20260826 for the verification Monte Carlo, 20260828 for the subject-level checks, 20260901 for the cohort duration check, and a design-dependent stream for the power simulation—allowing deterministic reproduction of every stochastic quantity reported in this article. All other reported values are analytic and do not depend on a seed.

## Supplementary Materials

The following supporting information can be downloaded at https://www.mdpi.com/article/10.3390/toxins18090403/s1, **Supplementary Materials S1—Full Mathematical Derivations and Modelling Assumptions.** Notation and the five modelling assumptions; the separation of the uptake count scale λ from the per-event cleavage yield η and the degeneracy it avoids; the terminal silencing probability and the direction of the mean-field error; what *N* counts, with the nested model of terminals within motor units and the bound on exposure sharing within a territory; the exact evaluation of the fibre layer for multiple endplates; the Beta threshold law and its quantal grounding; the dependence on the dominant-negative strength β; whether the shape of the threshold law matters; the mapping between the model’s cleaved fraction and what cleavage assays measure, with the terminal pool fraction ρ; the Gaussian diffusion field; the graded generalisation of the terminal response and the derivation of its steepness from quantal fluctuation; and the reference units. **Supplementary Materials S2—Proofs.** The stochastic mean-only benchmark and the claims withdrawn against it; the sharpness law with Berry–Esseen and lattice error control; the order of the approximation and a rigorous bound; the non-transportability corollary; geometry non-sufficiency and the critical dispersion radius. **Supplementary Materials S3—The Serotype Asymmetry in Cleavage–Effect Correlation.** Why a collective threshold decorrelates the assay from the outcome, compressing the outcome transition below the resolution the assay can sample; why the two serotypes differ through the dominant-negative strength, in which unit the resulting band must be read, and what reverses if it is read in the other; and what the account does and does not establish, including the claim about the correlation coefficient that is withdrawn and the superseded derivation of the ordering. **Supplementary Materials S4—Extended Dynamical Regimes.** Longitudinal threshold drift and effective uptake modulation, both phenomenological, with the candidate compensatory mechanisms and why they do not settle the question. **Supplementary Materials S5—Computational Verification and Reproducibility.** Environment and provenance; the reference implementation; numerical accuracy bounds including the exact validation of the Edgeworth expansion; verification of the critical regime; the synthetic recovery design and what it settles; sensitivity tables; the discriminator computation, with the exact quantile inversions for the three architectures and the detectability calculation behind them; the supporting figures; and the scope of the results. **Supplementary Materials S6—Spatial Geometry and Experimental Design.** The diffusion field and the critical dispersion radius; fractionated deposition, the optimal ring radius and the ordering of volume against site count; the design of the discriminating experiment, with the sample size, the power and the three departures that would invalidate it; what the collapse test requires of the targets compared; the three empirical tests; endpoint dependence, the coefficient that does not discriminate, and the published exponents; and what the sharpness law forbids. **Supplementary Materials S7—Duration Under a Minimal Recovery Law.** The extension that supplies a time axis and the reduction that keeps it exact; the duration invariant, its order of convergence and the sense in which the cancellation of the recovery timescale is dimensional rather than substantive; the closed form and the ingredient that fails in it; the sensitivity to the functional form of recovery, which is why the constant is withheld; the transport of the between-subject decomposition to the time axis in two terms and its failure in three; the calibration of the recovery dispersion from published relapse data and what that calibration cannot separate; and the negative finding that a duration endpoint is not more accessible than a dose endpoint. **Supplementary tables.** Supplementary Table S1, the nested model verified against direct simulation across innervation ratios and threshold correlations. Supplementary Table S2, the $N^{-1/2}$ exponent under the fibre layer. Supplementary Table S3, the fibre layer at the human reference, with the critical dose, the prefactor and the scale-free width against the number of endplates per fibre. Supplementary Table S4, the model carried through the dominant-negative strength β at fixed expected count. Supplementary Table S5, departure from mean-field sufficiency under the diffusion field at fixed spatial mean. Supplementary Table S6, quantal grounding of the unit threshold and the response steepness, in three panels: the trade-off surface in safety factor, the exact against the closed-form steepness, and the effect on the sharpness law. Supplementary Table S7, the reference parameter set with the status of each parameter and the ranges over which sensitivity was assessed. Supplementary Table S8, the four statements that a discrete threshold architecture might appear to support and does not. Supplementary Table S9, orders of convergence and the rigorous error bound. Supplementary Table S10, the closed form against exact binomial widths across more than two decades in *N*. Supplementary Table S11, verification of the sharpness law against exact binomial inversion and Monte Carlo. Supplementary Table S12, the transition width against unit number by exact binomial inversion, under the quantal response and in the binary limit. Supplementary Table S13, the scale-free transition width and the effective Hill exponent across target size. Supplementary Table S14, monotonicity of the silenced fraction in the dispersion radius. Supplementary Table S15, analytical and Monte Carlo agreement across the sub-critical, critical and supra-critical regimes. Supplementary Table S16, Monte Carlo convergence at the point of maximal estimator variance. Supplementary Table S17, recovery performance PR1–PR7 with the matched control PR5b. Supplementary Table S18, sensitivity to the uptake count scale and the degeneracy at low counts. Supplementary Table S19, the graded-response grid against the binary limit. Supplementary Table S20, the critical dispersion radius under fractionated deposition with the ring radius optimised. Supplementary Table S21, the one-factor-at-a-time parameter grid. Supplementary Table S22, the three architectures across target size, with the exact quantile inversions behind Table 3. Supplementary Table S23, the smallest displacement of the median effective dose detectable at the responder curves the published literature supplies. Supplementary Table S24, the critical dispersion radius under fractionated deposition at fixed total dose and fixed ring radius. Supplementary Table S25, the duration invariant against unit number, with the order of convergence and the cancellation of the recovery timescale. Supplementary Table S26, median duration and relative width against dose under exponential recovery. Supplementary Table S27, the share of observed variance carried by the finite-size term on the duration axis against the dose axis. **Supplementary figures.** Supplementary Figure S1, the sharpness law: transition width against independent-unit number, the collapse of its product with $\sqrt{N}$, and the dependence of the prefactor on threshold dispersion. Supplementary Figure S2, the non-transportability of a mean-only mapping matched at one target and applied to another. Supplementary Figure S3, longitudinal threshold drift and the corresponding minimum effective dose across sessions. Supplementary Figure S4, the block probability against fractional uptake impairment at three doses. Supplementary Figure S5, the distribution of the scale-free width *W* under joint sampling of the declared parameter ranges, with the first-order variance contributions. Supplementary Figure S6, geometry at matched mean dose: concentration profiles, the silenced fraction against dispersion radius, and the critical radius against dose. Supplementary Figure S7, duration under exponential recovery: median duration against dose at five recovery timescales, and the collapse of the scale-free product. Supplementary Figure S8, the finite-size share of observed variance on the duration axis against the dose axis, and the ratio between them. **Code and analysis outputs.** The analysis scripts and the machine-readable outputs underlying every reported figure and table, with a dependency specification, a test suite and a SHA-256 manifest. Environment, random seeds and the optimisation protocol are stated in Supplementary Materials S5.
