# Supplementary material for: Why the Same Dose of Botulinum Toxin A Is Less Predictable in Small Muscles: A Discrete Threshold Model
Source: Toxins (Basel). 2026 Sep 21;18(9):403. doi: 10.3390/toxins18090403 (PMC13611282; doi:10.3390/toxins18090403)
Supplement: Supplementary file 1 [file toxins-18-00403-s001.zip › Supplementary.pdf]

toxins

MDPI

Article

Supplementary Materials: Why the Same Dose of Botulinum Toxin A Is Less Predictable in Small Muscles: A Discrete Threshold Model

Andrea Felice Armenti and Francesco Armenti

Note on scope. This supplement documents the model as analysed in the main text and is self-contained. Three structural choices differ from earlier formulations of this model class, and each was adopted to repair a specific defect rather than as a matter of taste, so each is motivated in full below: *(i)* the uptake count scale λ and the per-event cleavage yield η are carried as separate parameters rather than collapsed into a single constant (Supplementary Materials S1.3), since the collapse silently forces approximately one productive entry per unit and renders the terminal threshold numerically inert; *(ii)* terminal thresholds follow a Beta law on $[0,1]$ rather than an unbounded log-normal law (Supplementary Materials S1.12), which otherwise assigns positive probability to units requiring more than complete cleavage; and *(iii)* spatial heterogeneity is represented by a Gaussian diffusion kernel rather than by a point-mass field (Supplementary Materials S1.18), since the latter makes the geometry result a restatement of mass conservation. In addition, the response steepness γ—previously a free parameter—is now derived from quantal fluctuation (Supplementary Materials S1.20), and the terminal threshold θ is *constrained* by the measured safety factor of the neuromuscular junction without being determined by it (Supplementary Materials S1.12). Closing that gap requires relating the model's f_i to what cleavage assays actually measure, which introduces one auxiliary quantity, the terminal pool fraction ρ, treated explicitly in Supplementary Materials S1.16. We report the value of ρ the model predicts rather than choosing the reading that suits it, and Supplementary Materials S1.16 states the alternative reading and its consequences in full. Supplementary Materials S2 proves the two propositions of the main text and states explicitly, as Lemma 1, the result that obliges us to *withdraw* two claims that a discrete threshold model of this kind might otherwise appear to support.

Supplementary Materials S1. Full Mathematical Derivations and Modelling Assumptions

S1.1 Notation and assumptions

The target muscle is a population of N discrete units indexed $i = 1, \ldots, N$. Throughout, C_i denotes the local extracellular toxin concentration at unit i, expressed in the reference units defined in Supplementary Materials S1.23, and $\bar{C} = N^{-1} \sum_i C_i$ is the spatial mean.

(A1) *Discreteness.* Toxin entry into a unit occurs as a countable number of productive translocation events.

(A2) *Sub-saturation.* Receptor occupancy is far from saturation over the clinical dose range, so the expected event count is proportional to local concentration.

(A3) *Threshold silencing.* A unit is silenced once the cleaved SNAP-25 fraction reaches a unit-specific threshold.

Check for updates

Received: 1 August 2026
Revised: 3 September 2026
Accepted: 5 September 2026
Published: 21 September 2026

Copyright: © 2026 by the author. Licensee MDPI, Basel, Switzerland. This article is an open access article distributed under the terms and conditions of the Creative Commons Attribution (CC BY) license.

(A4) *Conditional independence.* Given the field $\{C_i\}$, unit states are independent.

(A5) *Collective threshold.* Clinical block occurs once the silenced fraction reaches Θ_{sys}.

Assumption (A4) is the most consequential and is discussed critically in Supplementary Materials S1.7: it is what makes N an *independent-unit* count rather than an anatomical terminal count.

S1.2 Parameter status, result status, and the temporal scope

Parameters are of three kinds, distinguished in Supplementary Table S7: *derived* from independently measured quantities (μ_θ and γ, from the safety factor and quantal content); *declared* assumptions carried with their sensitivity (λ, Θ_{sys}, κ, N); and *constrained* by inverting a published measurement, which yields a falsifiable interval rather than a value (ρ, (7)). None is estimated by fitting the model to data. Results in this paper are of four kinds, and Table 1 identifies which is which. We separate them because a model of this class can generate a great deal of behaviour, and the value of any particular result depends on whether it is derived, computed, merely consistent with an observation, or assumed.

Two features of this pattern are worth separating. That a small measured fraction suffices is usually attributed to the dominant-negative action of the cleavage product SNAP-25_{197}, which is retained in the SNARE complex and inhibits release beyond the simple loss of intact protein [23]; how a small fragment population outcompetes a large intact pool remains an active question. But the absence of correlation is a separate matter, and it is a signature of a threshold: if outcome is binary in a collective variable, then an assay that varies smoothly across the transition will appear uncorrelated with an outcome that steps. Supplementary Materials S1.16 makes this quantitative and Section 3.12 turns it into a test.

Two consequences follow, and they differ. The absolute quantities of this paper are conditional on the readout time, exactly as they are conditional on the assay reading of Supplementary Materials S1.16; the falsifiable content is not, since a persistence acting as a common gain on effective exposure leaves the scale-free product W invariant (Corollary 3.7). What would move W is heterogeneous persistence, which enters $p'(\bar{C}_{\text{crit}})$ as threshold dispersion does—hence with an excursion on W of the order of the σ_θ row of Table 2—and is separable from it only by time-resolved measurement.

S1.3 Uptake: why λ and η must be separated

Under (A1)–(A2) the number of productive translocation events at unit i is

$$K_i \sim \text{Poisson}(\lambda C_i), \tag{S1}$$

with λ the expected number of such events per unit per unit concentration. Each event contributes multiplicatively to substrate depletion: if a single event removes a fraction η' of the remaining intact SNAP-25 pool, then after K_i independent events the cleaved fraction is

$$f_i = 1 - (1-\eta')^{K_i} \;=\; 1 - e^{-\eta K_i}, \qquad \eta := -\ln(1-\eta'), \tag{S2}$$

which is exact for multiplicative depletion and is the form used throughout.

Taking expectations and applying the Poisson probability generating function,

$$E[f_i \mid C_i] \;=\; 1 - E\Big[e^{-\eta K_i}\Big] \;=\; 1 - \exp\big[-\lambda C_i\big(1-e^{-\eta}\big)\big]. \tag{S3}$$

For small η, $1 - e^{-\eta} = \eta + O(\eta^2)$, so $E[f_i] \to 1 - e^{-\kappa C_i}$ with

$$\kappa := \lambda\eta. \tag{S4}$$

https://doi.org/10.3390/toxins18090403

Supplementary Equation (S4) defines the mean-field reduction used in the main text and shows that the *mean* response depends on λ and η only through their product.

The degeneracy that separation avoids. Because only κ enters the mean, it is tempting to carry a single constant through both the count and the catalytic step, writing $K_i \sim \text{Poisson}(\kappa C_i)$ with per-event yield κ. This is not a harmless reparameterisation. It forces $\lambda = \kappa$, and at reference values ($\kappa = 2$, $\bar{C} \approx 0.4$) the expected count becomes $\lambda\bar{C} \approx 0.8$, that is, below one productive event per unit. In that regime a single event already produces $f_i = 1 - e^{-\kappa} = 0.865$, which exceeds the entire support of the threshold distribution at $\mu_\theta = 0.50$. The unit is then silenced if and only if $K_i \geq 1$, the threshold θ_i performs no work, and the model collapses to

$$p(C) \;\approx\; F_\theta\!\left(1 - e^{-\kappa}\right)\left(1 - e^{-\lambda C}\right), \tag{S5}$$

a one-parameter Poisson entry gate. Supplementary Table S18 quantifies this: at $\lambda = 1$ a single entry suffices with probability one, and the probability falls below 10^{-6} for $\lambda \geq 10$, while the apparent discrepancy between the stochastic and mean-field formulations is a monotone function of λ alone, decaying from +150.2% to +0.5%. That discrepancy must therefore not be read as evidence that uptake stochasticity matters; in the degenerate corner it is a symptom of the collapse. The corner is more severe at the human reference than at the rodent one, because the halved threshold is cleared by a single event.

We adopt $\lambda = 35$ as the reference, giving $\eta = 0.0571$ and $E[K] = 13.5$ productive events per unit at the binary $\bar{C}_{\text{crit}}$ (14.3 at the graded one), with a Poisson coefficient of variation of 0.27. This is a declared assumption, not a measurement; Supplementary Materials S1.23 states what it does and does not fix, and Supplementary Table S18 reports the full dependence over $\lambda \in [1, 500]$.

S1.4 Separating λ from η, and what N counts

Why λ and η must be separated. The composite $\kappa := \lambda\eta$ governs the mean response, since $E[\eta K_i] = \kappa C_i$, so it is tempting to carry a single constant through both the count and the catalytic step. That collapse forces $\lambda = \kappa$, which at the reference parameters places the expected count below one event per unit: a single entry then produces $f_i = 0.865$, the threshold θ_i becomes numerically inert, and the model degenerates into a Poisson entry gate (Supplementary Materials S1.3). Terminal counts are inferred from muscle mass and typical innervation ratios rather than measured, and place the count at $N \approx$ 200–500 for small perioral muscles and 10,000–50,000 for large limb muscles; motor-unit number estimates, by contrast, are measured, and run to tens or low hundreds in human limb muscles [65,66]. For facial muscle a single direct estimate exists, 75 ± 15.6 motor units in dilator naris, with a test–retest coefficient of variation of 29.6% [52]; that reproducibility is itself a design constraint for Prediction 1. The operative range is accordingly $N \approx 10^1$–10^3, which is why Supplementary Table S13 extends down to $N = 20$ and why the shaded bands of Supplementary Figure S1 carry independent-unit counts. Motor-unit number estimation is the estimate we recommend, and Section 6 sets out why.

S1.5 Terminal silencing probability

Combining (S1), (S2) and (A3), the probability that a unit at local concentration C is silenced is

$$p^{\text{SU}}(C) \;=\; \sum_{k=0}^{\infty} \frac{e^{-\lambda C}(\lambda C)^k}{k!}\, F_\theta\!\left(1 - e^{-\eta k}\right), \tag{S6}$$

where F_θ is the threshold CDF. The mean-field reduction replaces the count by its expectation before applying the threshold,

$$p^{\mathrm{MF}}(C) \;=\; F_\theta\Big(1 - e^{-\kappa C}\Big). \tag{S7}$$

Both are smooth, strictly increasing in C on the relevant range, and satisfy $p(0) = 0$. These are the only properties used in Supplementary Materials S2.

Note that the weights $F_\theta(1 - e^{-\eta k})$ in (S6) do not depend on C. Evaluation on a grid of concentrations is therefore a single matrix–vector product, which the reference implementation exploits (Supplementary Materials S5.2).

S1.6 Direction of the mean-field error

Write $\phi(k) := F_\theta(1 - e^{-\eta k})$. Then $p^{\mathrm{SU}}(C) = E[\phi(K)]$ with $K \sim \mathrm{Poisson}(\lambda C)$, while $p^{\mathrm{MF}}(C) \approx \phi(E[K])$. By Jensen's inequality the sign of $p^{\mathrm{SU}} - p^{\mathrm{MF}}$ follows the convexity of ϕ over the effective support of K. Since ϕ is sigmoidal it is convex below and concave above its inflection, so no single inequality holds globally. Two practical consequences follow: the mean-field error must be evaluated numerically rather than bounded by convexity alone, which is what Supplementary Table S18 does, and the error shrinks as $\mathrm{CV}(K) = (\lambda C)^{-1/2}$ shrinks, since ϕ is then evaluated over an increasingly narrow range in which its curvature is nearly constant.

S1.7 Independence, and what N actually counts

Assumption (A4) is not innocuous, and we state its consequences plainly rather than defending it. Terminals belonging to a single motor unit share the neuronal determinants of their threshold—SNAP-25 expression, quantal content, safety factor, terminal morphology, all of which covary with motor-unit type—so their states are strongly, plausibly almost perfectly, correlated. In the perfectly correlated corner, terminals partitioned into M motor units of size m give

$$\mathrm{Var}(\Phi) \;=\; \frac{p(1-p)}{M}, \qquad N_{\mathrm{eff}} = M = N/m, \tag{S8}$$

so every result applies with N replaced by the motor-unit count, one to two orders of magnitude below the anatomical terminal count. That corner is an upper bound on the reduction, not the general case: Supplementary Materials S1.8 derives the latter.

Two consequences follow. First, all absolute transition widths reported here are *underestimates*: reducing N from 10^4 to 10^2–10^3 widens the transition by a factor of three to ten, which strengthens rather than weakens the clinical reading. Second, the substitution improves testability, because motor-unit counts are measurable by established electrophysiological methods whereas terminal counts are not. We therefore report results in terms of N throughout and state that N is to be instantiated as an independent-unit count.

Three departures from strict independence arise, and none changes the exponent: within-unit correlation of thresholds, entering through $N_{\mathrm{eff}} = N_{\mathrm{fib}}/[1 + (m_f - 1)r_S]$ with r_S the exchangeable intraclass correlation of the unit *states*; unequal weighting, through the effective sample size of a weighted mean (S9); and sharing of exposure within a motor-unit territory, through the bound (S13). Each reduces the effective count by a bounded factor and leaves the $N^{-1/2}$ scaling of Proposition 1 unaffected in form. The symbol r_S is used throughout for this intraclass correlation, r_θ for the intraclass correlation of the thresholds themselves, and ρ for the terminal pool fraction of Supplementary Materials S1.16; the three are unrelated. Because terminals of one motor unit are distributed across the target rather than co-located, a faithful model must nest terminals within motor units; Supplementary Materials S1.8 sets out that construction and verifies it against direct simulation.

https://doi.org/10.3390/toxins18090403

A second departure from (A4) acts through the same channel. The system integration of the main text weights every unit equally, whereas motor units differ in size and in their contribution to force by one to two orders of magnitude, so the clinically relevant aggregate is $\sum_i w_i S_i / \sum_i w_i$ rather than the unweighted mean. For weights independent of the unit states with coefficient of variation c_w, the variance of that aggregate is $p(1-p)\sum w_i^2/(\sum w_i)^2$, so

$$N_{\mathrm{eff}} = \frac{(\sum_i w_i)^2}{\sum_i w_i^2} \simeq \frac{N}{1+c_w^2}, \tag{S9}$$

the effective sample size of a weighted mean. We verified this by simulating 30,000 subjects at $N = 10{,}000$ with log-normal weights of unit median and $\sigma_w = \sqrt{\ln(1+c_w^2)}$, so that $c_w = 0.87$ and $c_w = 1.66$ correspond to forces spanning factors of twenty and of one hundred across four standard deviations: the interquartile range of the individual critical dose matches the unweighted computation at N_{eff} to within the Monte Carlo error of the check (0.67% at this number of subjects) in both cases, and the realised $(\sum w)^2/\sum w^2$ reproduces $N/(1+c_w^2)$ to 0.04% and 1.2% respectively, the second being sampling error on $\sum w^2$ under a heavy-tailed weight law. Unequal weighting therefore rescales the effective count by a bounded factor of roughly two to four and leaves the exponent of Proposition 1 unchanged, exactly as correlation does. Both departures are absorbed into the same statement: N is to be read as an effective independent-unit count, and the falsifiable content is the scaling rather than the count itself.

S1.8 The nested model: terminals within motor units

Supplementary Materials S1.7 states the consequence of within-unit correlation but assumes it rather than deriving it, and a reader is entitled to object that substituting the motor-unit count for the terminal count does not resolve the underlying tension. A motor unit is not a point in the concentration field: its terminals are distributed across the target, so they do not share a single C_i, and if the unit were the modelled entity then C_i, f_i and the position on the disc would have no referent. We therefore set out the nested construction and show what it reduces to.

The construction. The clinically relevant aggregate is generated fibre by fibre: each muscle fibre is driven by one neuromuscular junction, so the loss of force is a sum over *terminals*, not over motor units. Let there be M motor units of m_t terminals each, $N = Mm_t$, indexed (j,k). Where a fibre carries k endplates (Supplementary Materials S1.9) the fibre count per motor unit is $m_f = m_t/k$, and m_f is the index that enters the effective count below, since the fibre is the readout unit; at $k = 1$ the two coincide and the simulation of Supplementary Table S1 is computed there. Terminal (j,k) occupies position $\mathbf{x}_{jk}$ and sees the local concentration $C(\mathbf{x}_{jk})$ of Supplementary Equation (S27); the terminal count, the geometry and the cleavage relation are therefore unchanged from Supplementary Materials S1.3 to Supplementary Materials S1.5 and act at the level at which they physically act. What the motor-unit structure supplies is a correlation between the thresholds of sibling terminals, since SNAP-25 expression, quantal content, safety factor and terminal morphology are neuronal properties shared along one axon. We write this in latent form,

$$\theta_{jk} = F_\theta^{-1}\big(\Psi(z_{jk})\big), \qquad z_{jk} = \sqrt{r_\theta}\, u_j + \sqrt{1-r_\theta}\, \varepsilon_{jk}, \tag{S10}$$

with u_j and ε_{jk} independent standard normals, so that r_θ is the intraclass correlation of the thresholds and $r_\theta = 0$ recovers the model of Supplementary Materials S1.12 exactly.

What survives dichotomisation. The variance of Φ depends not on the correlation of the thresholds but on the correlation of the binary *states*, and thresholding attenuates it. At the operating point $p = \Theta_{\mathrm{sys}}$, so the latent threshold is $z^* = \Psi^{-1}(\Theta_{\mathrm{sys}})$ and

$$r_S = \frac{\Psi_2(z^*, z^*; r_\theta) - \Theta_{\mathrm{sys}}^2}{\Theta_{\mathrm{sys}}\,(1 - \Theta_{\mathrm{sys}})}, \tag{S11}$$

with Ψ_2 the bivariate standard normal distribution function. The attenuation is substantial over the plausible range: $r_\theta = 0.2$, 0.5 and 0.9 give $r_S = 0.126$, 0.330 and 0.711, that is factors of 0.63, 0.66 and 0.79. Substituting into Supplementary Equation (S8),

$$N_{\mathrm{eff}} = \frac{N_{\mathrm{fib}}}{1 + (m_f - 1)\, r_S}, \tag{S12}$$

and Proposition 1 holds with N replaced by N_{eff}. Supplementary Table S1 verifies this by simulating the nested model directly: across innervation ratios from 5 to 25 and threshold correlations from 0 to 0.9, the effective count recovered from the simulated transition width agrees with Supplementary Equation (S12) to within 2.2%.

The quantitative point. The reduction is *not* the innervation ratio. Dividing N by m is the $r_\theta = 1$ corner, in which sibling fibres are perfectly interchangeable; at $r_\theta = 0.5$ and $m_f = 10$ the surviving fraction is 0.25 rather than 0.10, and at $m_f = 50$ it is 0.058 rather than 0.020. The Limitations section of the main text quotes the innervation ratio as the scale of the reduction; that is the worst case, and Supplementary Equation (S12) is the general statement.

Territory, and why it does not change the exponent. The construction above takes terminal positions to be independent, which is the fully dispersed limit. Real motor units occupy territories, and terminals within a compact territory see similar concentrations, adding a second contribution to r_S. That contribution is bounded above by its point-territory limit,

$$\rho_{\mathrm{exp}} \le \frac{\mathrm{Var}_C[p(C)]}{\bar{p}\,(1 - \bar{p})}, \tag{S13}$$

the variance of the unit silencing probability across the field divided by its Bernoulli variance. At the operating point this evaluates to 0.62 at $s/R = 0.45$, 0.38 at $s/R = 0.60$, 0.077 at $s/R = 1.0$ and zero in the uniform limit; territories of finite extent fall below it, and the fully dispersed limit is zero. Exposure sharing therefore enters through the same channel as threshold sharing, is bounded by a computable quantity, and—like it—rescales N_{eff} without touching the exponent of Proposition 1.

What this settles and what it does not. It settles the objection that the model must choose between a terminal that has a position and a motor unit that fails independently: the modelled entity is the terminal throughout, and the motor unit enters only as a correlation structure. It settles the worry that the $N^{-1/2}$ scaling is an artefact of assuming independence: independence is not assumed, and the exponent survives correlation, unequal weighting and exposure sharing alike, each of which acts by rescaling the effective count. What it does not settle is the value of r_θ, of m, or of the territory extent in human facial muscle, for which we know of no measurements; N_{eff} therefore remains an assumed rather than a derived quantity, and the falsifiable content of this paper is the scaling and not the count.

https://doi.org/10.3390/toxins18090403

Supplementary Table S1. The nested model of Supplementary Equation (S10), verified by simulation. Each row draws 6,000 subjects with M motor units of m terminals; the effective count is recovered from the simulated interquartile range of the individual critical dose by inverting Proposition 1, and compared with Supplementary Equation (S12). The row with $r_\theta = 0$ is the independent case and returns N, as it must.

M	m_f	N	r_θ	r_S	N_{eff} predicted	N_{eff} recovered
1,000	10	10,000	0.00	0.000	10,000	10,025
1,000	10	10,000	0.20	0.126	4,687	4,669
1,000	10	10,000	0.50	0.330	2,521	2,515
1,000	10	10,000	0.90	0.711	1,352	1,348
400	25	10,000	0.50	0.330	1,122	1,145
2,000	5	10,000	0.50	0.330	4,313	4,333

S1.9 Multiple endplates per fibre: exact evaluation

Under the fibre relation of the main text, $q(\theta, C) = P(K \geq j(\theta))$ with $j(\theta) = \lceil -\ln(1-\theta)/\eta \rceil$ is a step function of θ with jumps at $\theta_j = 1 - e^{-\eta j}$. Hence

$$p_{\text{fib}}(C) = \sum_{j \geq 0} w_j [P(K \geq j)]^k, \qquad w_j = F_\theta(\theta_j) - F_\theta(\theta_{j-1}), \tag{S14}$$

exact and requiring no quadrature in θ. For $k = 1$, interchanging the two sums returns $\sum_i \text{Pois}(i)\, F_\theta(1 - e^{-\eta i}) = p^{\text{SU}}$ by Fubini; the implementation reproduces (S6) to 10^{-12} over the reference grid and over two off-reference parameterisations, which is the intended regression test.

Two limits are worth recording. In the mean-field reduction $q \in \{0, 1\}$, so $q^k = q$ and (S14) collapses to p^{MF} for every k; over a grid of four hundred concentrations the difference is identically zero in double precision, as an algebraic identity rather than a numerical coincidence. The layer is therefore invisible to any mean-concentration mapping, which is why the main text treats it as promoting rather than qualifying the role of λ. And as $\lambda \to \infty$ at fixed κ, $\text{CV}(K) \to 0$ and the fibre scaling relation of the main text returns the k-independent limit, as it must.

Supplementary Table S2. The $N^{-1/2}$ exponent under the fibre layer, at $k = 3$ and the human reference under the quantal response. The prefactor is rescaled by k; the exponent is not.

N	$\Delta\bar{C}_{0.75}$	$\Delta\bar{C}_{0.75}\sqrt{N}$
200	0.035899	0.50768
1,000	0.016141	0.51043
10,000	0.005111	0.51105
50,000	0.002286	0.51111

The comparator for the mean-field shift is defined only in the binary limit, since p^{MF} is itself binary and no graded mean-field reduction exists. Comparing the graded $\bar{C}_{\text{crit}}$ against it sums two effects—discrete uptake and graded response—and gives +11.8% at $k = 1$ rather than the +5.4% that the uptake approximation alone contributes. The figures quoted in the main text are therefore the binary ones throughout.

What we do not claim is a value of k for any named muscle. k joins r_θ, m and the territory extent in the list of quantities the architecture requires and the literature does not yet supply.

Supplementary Table S3. The fibre layer of Equation (9) at the human reference ($\kappa = 2.0$, $\lambda = 35$, $\mu_\theta = 0.50$, $\sigma_\theta = 0.075$, $\gamma = 5.93$, $\Theta_{sys} = 0.60$, $N = 10{,}000$), where k is the number of endplates per fibre. The critical dose and the prefactor rise together and nearly cancel in their ratio: $\bar{C}_{crit}$ by 77.9% and $\mathcal{K}$ by 73.1% across the range, against 10.0% for W, which is moreover not monotone. The last column reports the stochastic-uptake shift over the mean-field reduction in the binary limit, which is where the mean-field comparator is defined. W is the asymptotic value $\mathcal{K}/\bar{C}_{crit}$ of Equation (13) and n_{eff} is evaluated at $N = 10^4$; it coincides at $k = 2$ and $k = 3$ to the precision shown because W turns over between them.

k	$\bar{C}_{crit}$	shift vs. $k = 1$	$\mathcal{K}$	W	n_{eff}	SU vs. MF (binary)
1	0.4096	—	0.3779	0.9226	238.2	+5.4%
2	0.5296	+29.3%	0.4441	0.8386	262.0	+21.1%
3	0.6094	+48.8%	0.5111	0.8387	262.0	+29.6%
5	0.7288	+77.9%	0.6541	0.8975	244.8	+39.8%

S1.10 The fibre layer: tabulated values and the scaling law

The dose shift itself obeys an approximate scaling law. Writing $\mathrm{CV}(K) = (\lambda\bar{C}_{crit})^{-1/2}$,

$$\frac{\bar{C}_{crit}(k) - \bar{C}_{crit}(1)}{\bar{C}_{crit}(1)} \simeq c_k\,\mathrm{CV}(K), \tag{S15}$$

with $c_2 = 0.551$, $c_3 = 0.849$, $c_4 = 1.051$, $c_5 = 1.203$ and $c_8 = 1.511$. The form is good for small k and degrades for large: c_k varies over $\lambda \in [10, 500]$ by 2.3% at $k = 2$ and by 12.1% at $k = 8$, and $c_k/\ln k$ falls monotonically from 0.795 to 0.726, so the growth is near-logarithmic without being logarithmic. We report Supplementary Equation (S15) as an approximation valid for $k \lesssim 5$ and not as a law.

Supplementary Table S2 verifies the exponent under the layer directly: at $k = 3$ the product $\Delta\bar{C}_{0.75}\sqrt{N}$ takes the values 0.50768, 0.51043, 0.51105 and 0.51111 at $N = 200$, 10^3, 10^4 and 5×10^4, an excursion of 0.67%.

The layer acts in opposite directions on the two quantities, and the second direction favours the falsifiable content. Because every endplate must fail, $\bar{C}_{crit}$ rises with k and with it the expected count $E[K] = \lambda\bar{C}_{crit}$; at high $E[K]$ the Poisson lattice ceases to be resolved against the threshold dispersion, and λ stops mattering to the width. The excursion of W over $\lambda \in [10, 100]$ is 34.9% at $k = 1$, 13.9% at $k = 2$ and 4.5% at $k = 3$, where it is moreover non-monotone with an interior minimum near $\lambda \approx 30$. In a multiply innervated muscle λ therefore governs where the transition sits more strongly, and how wide it is more weakly, than at $k = 1$; the λ row of Table 2 is the singly innervated case and is the conservative one for the measurable quantity.

S1.11 The fibre layer: construction, the unit hierarchy, and what stochasticity carries

Three consequences follow, and together they specify the layer exactly. Sibling endplates on one fibre share the neuronal determinants of their threshold, so θ is common to them. Their separation is two orders of magnitude below any plausible dispersion radius, so they see one local concentration C. But uptake is a counting process at each endplate separately, so the counts K are independent. A fibre generates its action potential if any one endplate transmits; hence, it fails only if all k fail:

Because q is piecewise constant in θ, with jumps at $\theta_j = 1 - e^{-\eta j}$, the expectation is a finite weighted sum and is evaluated exactly rather than by quadrature (Supplementary Materials S1.9). At $k = 1$ (9) reduces identically to the unit silencing probability of Supplementary Materials S1.5.

The readout unit is therefore the *fibre*: force is a sum over fibres, and the count entering Proposition 1 is $N_{fib} = N_{term}/k$, reduced further by motor-unit correlation as Section 6

sets out. The full hierarchy is $N_{\text{term}} = k\,N_{\text{fib}}$, $N_{\text{fib}} = m_f\,N_{\text{MU}}$, $N_{\text{eff}} = N_{\text{fib}}/[1 + (m_f - 1)r_S]$, with m_f the number of fibres per motor unit. No value of k is claimed for any named muscle—the distribution of k in human facial muscle is not reported [32]—so results are given as functions of k throughout, and k enters the measurable width through $\mathrm{CV}(K)$ with an excursion of 10.0% over $k = 1$–3, and non-monotonically (Table 2).

Stochasticity of the output does not. Y is binary, so it is stochastic by construction rather than by modelling choice—the clinical readout is a yes/no judgement—and by the benchmark lemma of Supplementary Materials S2 its variance is fixed once its mean is.

Stochasticity of uptake does not, for a singly innervated fibre. Retaining Poisson counts rather than their expectation shifts $\bar{C}_{\text{crit}}$ by 5.44% at the reference count scale, and by less as the scale grows (Section 4.6). Two further withdrawals are local to the supplement and are recorded there: a sharpness constant for the alternative assay reading, computed inside the degeneracy of Supplementary Materials S1.3 and therefore invalid (Supplementary Materials S1, Remark 2), and a claim that κ is structurally identifiable, which a numerical search cannot establish (Supplementary Materials S5.7).

S1.12 Threshold distribution: mechanistic grounding, and why Beta and not log-normal

Because $f_i \in [0, 1]$ is a fraction, a threshold above unity describes a unit requiring more than complete cleavage, hence one that cannot be silenced at any dose. The threshold law must therefore be supported on $[0, 1]$. We take

$$\theta_i \sim \mathrm{Beta}(a, b), \qquad a = \mu_\theta \nu, \quad b = (1 - \mu_\theta)\nu, \quad \nu = \frac{\mu_\theta(1 - \mu_\theta)}{\sigma_\theta^2} - 1, \tag{S16}$$

which realises any admissible mean–variance pair on the unit interval and requires $\sigma_\theta^2 < \mu_\theta(1 - \mu_\theta)$. At the human reference values $\mu_\theta = 0.50$ and $\sigma_\theta = 0.075$ this gives $\mathrm{Beta}(21.722, 21.722)$ and $f^*_{\text{sys}} := F_\theta^{-1}(\Theta_{\text{sys}}) = 0.5193$; the rodent parameterisation $\mu_\theta = 0.70$, $\sigma_\theta = 0.105$ gives $\mathrm{Beta}(12.633, 5.414)$ and $f^*_{\text{sys}} = 0.7345$.

An unbounded surrogate with the same mean and coefficient of variation—a log-normal law, as used in earlier formulations—assigns $P(\theta > 1) = 0.68\%$ under the rodent parameters. Those units are never silenced, the attainable silenced fraction is capped at $E[\Phi]_{\max} = 0.9932$, and the model acquires a ceiling that is an artefact of the distributional choice rather than a property of the mechanism. Since the main text draws conclusions about attainability (Supplementary Materials S4.2), the artefact is not cosmetic and the bounded law is required.

What constrains θ. The location of the threshold is not free, though it is not fully determined either. Endplate potential amplitude is proportional to the quantal content m, the number of vesicles released per nerve impulse, and transmission is secured by a *safety factor* $\mathrm{SF} = \mathrm{EPP}/\mathrm{AP}$ threshold, reported across mammalian muscles in the range 2–10 [27], and at about 2 in human against about 4 in rodent, human terminals releasing only 20–30 quanta per impulse [69]. Transmission fails when $m < m_0/\mathrm{SF}$.

Let N_S be the number of intact SNARE complexes required per fusion event and $\beta \geq 0$ the dominant-negative strength of the cleavage product, so that the functionally available fraction of intact SNAP-25 is $a(f) = (1 - f)/(1 + \beta f)$ and

$$\frac{m(f)}{m_0} = \big[a(f)\big]^{N_S}. \tag{S17}$$

Setting $m(\theta)/m_0 = 1/\mathrm{SF}$ and writing $A := \mathrm{SF}^{-1/N_S}$ gives $1-\theta = A(1+\beta\theta)$; hence

$$\theta = \frac{1-A}{1+\beta A}, \qquad \mathrm{SF} = \left[\frac{1+\beta\theta}{1-\theta}\right]^{N_S}. \tag{S18}$$

Supplementary Equation (S18) links three quantities of which only SF is measured, so it delivers a trade-off surface rather than a value (Supplementary Table S6(a)). Two features of that surface matter. First, $N_S \geq 2$ demands safety factors of 11 or more at any $\beta \geq 0$, far outside the measured range; the framework is therefore committed to release scaling approximately linearly with functionally intact SNAP-25, with the dominant-negative term β carrying any additional steepness. Second, along the admissible $N_S = 1$ line the threshold ranges from $\theta = 0.80$ at $(\mathrm{SF}, \beta) = (5, 0)$ down to arbitrarily small values as β grows. The safety factor alone does not pick a point on that line.

What we do not claim. Inverting Supplementary Equation (S18) at $\beta = 0$ returns $\mathrm{SF} = 3.33$ for $\mu_\theta = 0.70$, which falls inside the measured range and is tempting to present as a consistency check the reference value has passed. That inference would be circular in a specific way: it sets $\beta = 0$ while simultaneously invoking the dominant-negative action of SNAP-25_{197} as the mechanistic justification for a sharp threshold. The two cannot both hold, and we therefore do not offer the agreement as support. What the safety factor supports is the *form* of Supplementary Equation (S18) and the exclusion of $N_S \geq 2$; it does not by itself validate $\mu_\theta = 0.70$.

Dispersion. Whatever the location, the dispersion follows from the same relation. Differentiating (S18) at $\beta = 0$, $N_S = 1$ gives $d\theta/d\mathrm{SF} = \mathrm{SF}^{-2}$; hence

$$\mathrm{CV}(\theta) = \frac{\mathrm{CV}(\mathrm{SF})}{\mathrm{SF}\,\theta}, \tag{S19}$$

so under the rodent parameterisation $\sigma_\theta = 0.105$ corresponds to $\mathrm{CV}(\mathrm{SF}) = 0.35$, that is $\mathrm{SF} = 3.33 \pm 1.17$. The relation runs the other way at the human reference and constrains it. Carrying $\mathrm{CV}(\mathrm{SF}) = 0.35$ across to $\mathrm{SF} = 2$ would give $\sigma_\theta = 0.175$, under which the implied distribution of $\mathrm{SF}_i = 1/(1-\theta_i)$ places its fifth percentile at 1.27 and a 30% quantal run-down would fail 14% of junctions, against 0.31% at $\sigma_\theta = 0.075$. The former is incompatible with the absence of transmission failure at maximal voluntary exertion [27], and we therefore hold $\mathrm{CV}(\theta) = 0.15$ fixed instead, which is the choice adopted in the main text. It carries a prediction: the model requires $\mathrm{CV}(\mathrm{SF}) \lesssim 0.15$ in human muscle.

S1.13 The threshold law, the two ceilings, and the collective threshold

The Beta law (S16) realises any admissible mean and variance on the unit interval. At the human reference values $\mu_\theta = 0.50$ and $\sigma_\theta = 0.075$ (Section 4.11) this gives $\mathrm{Beta}(21.72, 21.72)$; the rodent parameterisation $\mu_\theta = 0.70$, $\sigma_\theta = 0.105$ gives $\mathrm{Beta}(12.63, 5.41)$ and is retained for comparison. An unbounded surrogate such as a log-normal law with the same mean and coefficient of variation assigns positive mass above unity—0.68% of terminals under the rodent parameters—capping the attainable silenced fraction at 0.9932 and introducing a spurious ceiling absent from the model as specified.

A ceiling on the attainable silenced fraction also arises from the quantal terminal response of Supplementary Materials S1.21, where it is larger: $\phi_{\max} = 0.9787$ against 0.9932 for the log-normal surrogate. The two are not the same object and are not treated alike. The log-normal ceiling is structural: a unit with $\theta > 1$ requires more than complete cleavage and cannot be silenced by any mechanism at any dose, so the ceiling is an artefact of a distributional choice with no referent in the biology. The quantal ceiling is a per-impulse failure probability below one at complete cleavage, which is what quantal

transmission theory states rather than an artefact: a junction with a low threshold still transmits occasionally. The first is removed by bounding the threshold law, which is why (S16) is adopted; the second is a property of the mechanism and is retained, and Section 3.2 derives a consequence from it.

Let N_S denote the number of intact SNARE complexes required per fusion event and $\beta \geq 0$ the dominant-negative strength of the cleavage product, so that the functionally available fraction of intact SNAP-25 is $a(f) = (1-f)/(1+\beta f)$ and

$$\frac{m(f)}{m_0} = \big[a(f)\big]^{N_S}. \tag{S20}$$

Setting $m(\theta)/m_0 = 1/\mathrm{SF}$ and writing $A := \mathrm{SF}^{-1/N_S}$ gives the threshold in closed form,

$$\theta = \frac{1-A}{1+\beta A}, \qquad \text{equivalently} \qquad \mathrm{SF} = \left[\frac{1+\beta\theta}{1-\theta}\right]^{N_S}. \tag{S21}$$

Supplementary Equation (S21) links three quantities of which only SF is measured, so it delivers a trade-off surface rather than a value (Supplementary Materials S1.12). Two features of that surface matter: $N_S \geq 2$ would require safety factors of 11 or more, far outside anything reported, so the framework is committed to release scaling approximately linearly with functionally intact SNAP-25, with the dominant-negative term carrying any additional steepness, and along the admissible $N_S = 1$ line the safety factor alone does not fix θ, which depends on a separate question taken up next.

The influence of the collective threshold Θ_{sys} is not what the form of (S35) suggests: over $\Theta_{\mathrm{sys}} \in [0.5, 0.7]$ both $\mathcal{K}$ and $\bar{C}_{\mathrm{crit}}$ move substantially, because $p'(\bar{C}_{\mathrm{crit}})$ moves with the operating point, but the two displacements very nearly cancel and the measurable width W varies by 7.39% over $\Theta_{\mathrm{sys}} \in [0.5, 0.7]$ (Table 2). That interval is not arbitrary and the figure is not typical: W has an interior minimum near $\Theta_{\mathrm{sys}} = 0.50$ and $[0.5, 0.7]$ is centred on it, so 7.39% is the best value obtainable rather than a representative one. Over $[0.30, 0.80]$ the excursion is 23.92%. The cancellation nonetheless operates, and by a wide margin: across that range $\bar{C}_{\mathrm{crit}}$ moves by 112% and $\mathcal{K}$ by 140%, against 24% for their ratio. The insensitivity of the falsifiable form is a cancellation and not a small partial derivative.

S1.14 Dependence on the dominant-negative strength β

Both quantities Supplementary Materials S1.12 and Supplementary Materials S1.20 derived from the safety factor—the unit threshold θ and the response steepness γ—are obtained at $\beta = 0$, while Supplementary Materials S3.2 attributes the behaviour of BoNT/A to a large β. The two are consistent only if the consequences of β are stated, which is what this section does. Nothing here is a free choice: β enters the model at exactly one place, Supplementary Equation (S18), and everything else follows.

The inverse relation. For $N_S = 1$, inverting (S18) gives

$$\beta = \frac{\mathrm{SF}\,(1-\theta) - 1}{\theta}, \tag{S22}$$

exact in double precision against the forward map. The threshold of reading (ii), $\theta = 0.125$, therefore requires $\beta = 6.000$ at the human SF $= 2$ and $\beta = 15.31$ at the rodent SF $= 3.33$. The larger figure is the rodent one and does not transfer.

Response steepness at $\beta > 0$. Differentiating (S29) at $f = \theta$ with the full $a(f) = (1-f)/(1+\beta f)$ rather than at $\beta = 0$, and matching to the logistic slope $\gamma/(4\theta)$ as in Supplementary Materials S1.20, gives

$$\gamma_q(\beta) \simeq 4\,\theta\,\varphi(0)\,N_S\,\sqrt{\bar{m}_0\,\mathrm{SF}}\;\mathrm{SF}^{1/N_S-1}\,\frac{1+\beta}{(1+\beta\theta)^2}, \tag{S23}$$

which reduces to (S31) at $\beta = 0$. The derivation was carried out symbolically for general N_S and agrees with (S23) identically; a finite-difference check on P_{fail} agrees to 2×10^{-10} relative at every β in Supplementary Table S4. Since θ itself falls with β, the net effect is a monotone decrease in steepness: raising β lowers the threshold and flattens the response together.

What moves, and what nearly cancels. Supplementary Table S4 carries β through the model at $\mathrm{CV}(\theta) = 0.15$ held fixed, so $\sigma_\theta = 0.15\,\theta(\beta)$, with γ taken from the exact Poisson computation and λ renormalised at each β so that the expected count at the operating point is unchanged. Over $\beta \in [0, 15]$ the critical concentration falls by a factor of eleven and the prefactor by a factor of ten, while their ratio W moves by 13.2%; over $\beta \in [0, 6]$, which spans both readings of Supplementary Materials S1.16, by 10.0%. The scale-free width is thus far less sensitive to β than the absolute quantities, but it is not invariant to it, and we report it as a conditional quantity in Table 2 rather than as an invariance. The last two columns carry the consequence for the serotype account: the band coefficient of (21) rises with β in relative units and falls in absolute ones, and Supplementary Materials S3.2 sets out which of the two the prediction is stated in.

The renormalisation is not cosmetic. Holding $\lambda = 35$ fixed instead, the excursion of W rises to 78.3%. That figure should not be read as a dependence on β: at fixed λ the expected count falls from 14.3 at $\beta = 0$ to 1.7 at $\beta = 15$, and every row with $\beta \geq 1$ sits in the degenerate corner of Supplementary Materials S1.3, where $\eta(1-\theta)/\sigma_\theta$ reaches 6.1 and the Poisson lattice is coarser than the threshold dispersion. The widening there is numerical, not structural. The renormalisation is the same one Supplementary Materials S1.16 already applies to reading (ii), and Supplementary Materials S5.9 shows that holding $E[K]$ fixed is exactly equivalent to holding $\eta = \kappa/\lambda$ fixed.

S1.15 Does the shape of the threshold law matter?

The Beta law (S16) is adopted because it is supported on $[0, 1]$, not because it is established. Since the prefactor depends on the density at the critical quantile and not on the variance alone, the choice appears consequential. We bound it rather than declare it.

If $\theta = 1 - 1/\mathrm{SF}$ and SF carries a distribution, the induced threshold law is determined, $f_\theta(\theta) = f_{\mathrm{SF}}\big[(1-\theta)^{-1}\big](1-\theta)^{-2}$, and is not Beta. Taking SF log-normal and gamma and matching the first two moments of θ to the reference $(0.50, 0.075)$, the density at the critical quantile varies by 10.4% across the three families and the mean-field prefactor by 9.1%. In the primary stochastic-uptake formulation, however, $\mathcal{K}$ varies by 0.14% and W by 0.25%. Poisson uptake convolves the threshold law before the collective integration, and the stochastic slope is governed by that convolution rather than by $f_\theta(f^*)$.

There are two consequences. The constants reported in this work are not properties of the Beta family, so the concern is answered rather than acknowledged. And the sensitivity of the mean-field reduction to a choice the primary model is insensitive to is a further reason to report the two formulations in pairs, as Supplementary Materials S1.3 already requires on other grounds.

Supplementary Table S4. The model carried through β at SF = 2, $N_S = 1$, $\bar{m}_0 = 25$, CV$(\theta) = 0.15$, $\kappa = 2.0$, $\Theta_{\text{sys}} = 0.60$, $N = 10^4$. γ is the exact Poisson value; λ is renormalised at each row so that $E[K] = \lambda\bar{C}_{\text{crit}} = 14.34$, the reference value. $\bar{C}_{\text{crit}}$ and $\mathcal{K}$ are the graded readings, and W is the finite-size scale-free width $(\Delta\bar{C}_{0.75}/\bar{C}_{\text{crit}})\sqrt{N}$ evaluated at the tabulated $N = 10^4$ by exact binomial inversion—not the asymptotic ratio $\mathcal{K}/\bar{C}_{\text{crit}}$ reported in Supplementary Table S3, which is larger by about 1×10^{-4} in every row. The first row is the human reference, computed at the exact Poisson $\gamma = 5.9319$ rather than at the rounded 5.93 carried through the main text; that difference displaces the last digit of each entry and accounts for 0.9223 here against 0.9225 at $N = 10^4$ in Supplementary Table S13. $\bar{C}_{\text{crit}}$ moves by a factor of eleven across the table and $\mathcal{K}$ by a factor of ten; W moves by 13.2%. The last three columns carry the consequence for Supplementary Materials S3.2: $\langle f \rangle$ is the mean cleaved fraction at the operating point, $B(\langle f \rangle)W$ the band coefficient of (21) in relative units, which rises by 66.9% across the table, and $h(\langle f \rangle)W$ the same band in points of measured cleavage, $h(x) = (1-x)\ln[1/(1-x)]$, which falls by a factor of 4.7. The two move in opposite directions because the operating point falls faster than the relative band widens.

β	θ	γ	λ	$\bar{C}_{\text{crit}}$	$\mathcal{K}$	W	$\langle f \rangle$	$B(\langle f \rangle)W$	$h(\langle f \rangle)W$
0	0.5000	5.932	35.0	0.40959	0.37780	0.9223	0.5490	0.6033	0.3312
1	0.3333	4.534	59.8	0.23987	0.22730	0.9475	0.3761	0.7415	0.2789
2	0.2500	4.025	84.0	0.17065	0.16551	0.9697	0.2863	0.8154	0.2334
4	0.1667	3.592	132.0	0.10858	0.10837	0.9980	0.1939	0.8943	0.1734
6	0.1250	3.397	179.9	0.07971	0.08087	1.0144	0.1466	0.9362	0.1372
10	0.0833	3.215	275.3	0.05208	0.05379	1.0326	0.0986	0.9800	0.0966
15	0.0588	3.112	394.4	0.03635	0.03796	1.0442	0.0699	1.0068	0.0704

S1.16 Relating the model to cleavage assays: the pool fraction ρ

The quantity f_i in (S1)–(S2) is the cleaved fraction of the SNAP-25 pool that participates in release *at unit i*. Published cleavage figures are not that quantity, and the difference is methodological rather than interpretive. Those figures are densitometric estimates from immunoblots of whole-tissue or whole-culture lysates: the denominator is all neuronal SNAP-25 in the preparation—terminal, preterminal, axonal, somatic—and the numerator is summed over every unit, intoxicated or not. Writing ρ for the fraction of neuronal SNAP-25 residing in the releasable terminal pool,

$$\text{measured cleavage} \;=\; \rho\,\langle f \rangle, \tag{S24}$$

with $\langle f \rangle$ the mean of f_i over units. Under the uniform field this has a closed form,

$$\langle f \rangle(\bar{C}) \;=\; 1 - \exp\!\big[-\lambda\bar{C}\big(1 - e^{-\eta}\big)\big], \tag{S25}$$

by the Poisson probability generating function, giving $\langle f \rangle = 0.5490$ at $\bar{C}_{\text{crit}}$.

What the data then fix. Cleavage of 10–15% of total SNAP-25 abolishes transmission at the frog neuromuscular junction [20] and in rodent spinal cord cultures [21], with 2–3% sufficient to silence miniature postsynaptic currents [22]. A mammalian *in vivo* anchor is also available: in rat gastrocnemius paralysis was probable once the ratio of cleaved to intact SNAP-25 exceeded 0.35 [28], a cleaved fraction of 0.259. And in mouse hemidiaphragm the loss of SNAP-25 immunoreactivity was under 10% one hour after paralysis and above 75% four hours later [29], which is an independent confirmation of the separation (S24) makes, obtained without reference to any model. Inverting (S24) at the two extremes,

$$\rho \;=\; 0.125/0.5490 \;\simeq\; 0.23 \qquad \text{and} \qquad \rho \;=\; 0.259/0.5490 \;\simeq\; 0.47. \tag{S26}$$

This is a prediction, not an added degree of freedom. The model plus the measurements jointly bound ρ to a factor of two, set by which preparation the anchor is taken from; the

quantity is in principle accessible by subcellular fractionation or quantitative imaging; and a measured value near unity refutes the present parameterisation outright.

The alternative reading, and why we adopt the first. Supplementary Equation (S24) admits a second internally consistent resolution: $\rho \approx 1$, so that the measured cleavage *is* the terminal cleavage, the unit threshold is $\theta \approx 0.125$, and reconciling this with measured safety factors requires a dominant-negative strength $\beta \approx 6$ at the human SF $= 2$, or $\beta \approx 15$ at the rodent one (Supplementary Materials S1.14). We adopt reading (i)—$\rho \in [0.23, 0.47]$ with $\theta = 0.50$ at the human reference—for the numerical work, and record what changes under the alternative.

Remark 1 (Reading (ii) requires the count scale to be rescaled with it)**.** *Under* $\theta \approx 0.125$ *the critical concentration falls by roughly an order of magnitude, and at the reference* $\lambda = 35$ *the expected count at the operating point becomes* $E[K] = 3.5$ *with* $\mathrm{CV}(K) = 0.71$ *and a single-event step in cleaved fraction* $\eta(1-\theta) = 0.088$ *against a threshold dispersion of* 0.019*. That is precisely the degenerate corner of Supplementary Materials S1.3: the unit is silenced if and only if* $K \geq 2$ *and the threshold law performs no work. Reading (ii) must therefore be evaluated at a count scale that preserves the operating point,* $\lambda \approx 150$*, and not at* $\lambda = 20$*. Doing so gives* $\bar{C}^{\mathrm{SU}}_{\mathrm{crit}} = 0.0739$ *against* $\bar{C}^{\mathrm{MF}}_{\mathrm{crit}} = 0.0691$ *(a shift of* $+7.0\%$*, comparable to the* $+5.4\%$ *of reading (i)),* $E[K] = 11.1$*,* $\mathrm{CV}(K) = 0.30$*,* $\eta(1-\theta)/\sigma_\theta = 0.62$*, and* $\mathcal{K} = 0.0430$*. The product* $\Delta\bar{C}_{0.75}\sqrt{N}$ *is then constant at* 0.0430 *from* $N = 200$ *to* $N = 20{,}000$*, so the* $N^{-1/2}$ *exponent, geometry non-sufficiency and the recovery ordering are unaffected; only the constants move. We note that the constant quoted for reading (ii) in earlier versions of this work was computed at* $\lambda = 20$ *and therefore inside the degenerate corner, and we withdraw it.*

The two readings make opposite predictions for ρ, so the question is empirical rather than a matter of preference, and Supplementary Materials S1.16 reports Supplementary Equation (S26) as the falsifiable consequence of the reading we adopt. Absolute concentrations are conditional on that choice throughout; ratios, exponents and the scale-free product are not.

S1.17 The two readings of the pool fraction, and the comparison class

Two readings are therefore open, and Supplementary Materials S1.16 sets both out in full. Under *(i) dilution*, $\rho \in [0.23, 0.47]$ and the unit threshold is set by the measured safety factor, so the small measured fractions are small chiefly because most neuronal SNAP-25 is not in the releasable pool. Under *(ii) strong dominant-negative*, $\rho \approx 1$, the unit threshold falls to $\theta \approx 0.125$, and reconciling this with the measured safety factor requires $\beta \approx 6$ at the human reference (SF $= 2$), or $\beta \approx 15$ at the rodent one.

Both readings fix the threshold from a safety factor of 3–5, which is the rodent figure; in human the safety factor is about 2, against about 4 in rodent [27,69], and Section 4.11 carries this through as a third, species-specific reading that changes every constant in this paper and no exponent.

The identification demands a comparison class hard enough to be informative. We adopt throughout $Y \sim \mathrm{Bernoulli}(g(\bar{C}))$, a stochastic mean-only model, rather than the deterministic mapping $R = g(\bar{C})$ that such comparisons usually take as the benchmark (Supplementary Materials S2.1). Two properties of the discrete model therefore carry no structural information and are recorded as observations rather than results (Section 3).

https://doi.org/10.3390/toxins18090403

S1.18 Spatial field: why a diffusion kernel and not a point mass

Geometry enters through the field $\{C_i\}$ at fixed spatial mean. We take units uniformly distributed on a disc of radius R, injection at the centre, and a Gaussian profile with dispersion radius s,

$$C(r) = \frac{\bar{C}\, e^{-r^2/2s^2}}{\left\langle e^{-r^2/2s^2} \right\rangle_R}, \qquad \langle g \rangle_R := \frac{2}{R^2} \int_0^R g(r)\, r\, dr, \tag{S27}$$

normalised so that the mean over units equals $\bar{C}$ exactly for every s. The limit $s/R \to \infty$ recovers the uniform field. Substituting $u = r^2/R^2$ turns (S27) into an integral over $u \in [0, 1]$ with uniform weight, so Gauss–Legendre quadrature in u converges rapidly; 80 nodes give agreement to machine precision (Supplementary Materials S5.4).

Earlier formulations of this model class established geometry dependence by comparing a uniform field against a "focal" point mass, in which the entire dose is assigned to one unit and the concentration is exactly zero elsewhere. That comparison does produce a large effect, but trivially: with all mass on a single unit, $\Phi \leq 1/N$ by counting alone, so $P(Y{=}1) = 0$ whenever $1/N < \Theta_{\text{sys}}$ follows from mass conservation and not from any property of the response. No physical diffusion field has exact zeros. Supplementary Equation (S27) is a decaying kernel with support everywhere on the domain, so the geometry effect it produces is not automatic, and its natural summary, the critical dispersion radius s^* of Proposition 2, maps onto injection volume and dilution.

Supplementary Table S5 reports the departure $\langle p(C_i) \rangle - p(\bar{C})$ across doses and dispersion radii. It is large and negative for focal fields and vanishes as $s/R \to \infty$, as it must.

Supplementary Table S5. Departure from mean-field sufficiency, $\langle p(C_i) \rangle - p(\bar{C})$, under the diffusion field (S27) at fixed spatial mean. A mean-only benchmark predicts $p(\bar{C})$ irrespective of s; the discrete model does not. The departure vanishes in the uniform limit, confirming that the effect is produced by spatial spread and not by a normalisation artefact. Computed at the rodent parameterisation, retained here because the table demonstrates a structural property rather than a parametric result; at the human reference the entries are some four percent larger in magnitude and the pattern is unchanged.

	dispersion radius s/R		
mean dose $\bar{C}$	0.4	0.8	2.0
$0.8\,\bar{C}_{\text{crit}}$	−0.0349	+0.0007	+0.0000
$1.0\,\bar{C}_{\text{crit}}$	−0.1776	−0.0279	−0.0008
$1.5\,\bar{C}_{\text{crit}}$	−0.3629	−0.0520	−0.0014
$2.5\,\bar{C}_{\text{crit}}$	−0.2855	−0.0069	−0.0001

S1.19 Why a decaying kernel and not a point mass

This one-parameter family replaces the point-mass ("focal") construction used in earlier formulations of this class of model, in which the entire dose is assigned to a single terminal and the concentration is exactly zero elsewhere. That construction does produce a large geometry effect, but trivially: with all mass on one terminal, $\Phi \leq 1/N$ by counting alone, so the result is a restatement of mass conservation rather than a property of the response, and no physical diffusion field has exact zeros. Equation (10) is a decaying kernel with support everywhere on the domain, the geometry effect it produces is therefore not automatic, and its natural summary—the critical dispersion radius s^* below which block is unattainable at a given dose (Supplementary Materials S6.1)—maps onto injection volume and dilution, which are quantities the clinician actually controls.

https://doi.org/10.3390/toxins18090403

S1.20 Binary terminal state and its graded generalisation

The binary indicator $S_i = \mathbb{I}(f_i \geq \theta_i)$ is an idealisation of a steep but continuous cleavage-to-release map. Its mechanistic justification is the dominant-negative behaviour of the cleavage product: SNAP-25_{197} is retained in the SNARE complex and inhibits exocytosis beyond the simple loss of intact SNAP-25, so effective release probability falls faster than the cleaved fraction rises. We replace the indicator by

$$S_i^{\mathrm{gr}} = [1 + (\theta_i / f_i)^{\gamma}]^{-1}, \tag{S28}$$

recovering the binary case as $\gamma \to \infty$. Supplementary Table S19 reports the transition width across γ.

The requirement is demanding: agreement within 10% of the binary limit requires $\gamma \gtrsim 20$ at the human reference. Rather than leave γ as a free parameter, we derive it from the same quantal argument that fixed θ in Supplementary Materials S1.12.

Derivation of γ from quantal fluctuation. Quantal content is a random variable. Taking $m \sim \mathrm{Poisson}(\bar{m}(f))$ with $\bar{m}(f) = \bar{m}_0\,[a(f)]^{N_S}$ from (S17), and transmission failure when $m < m_{\mathrm{crit}} := \bar{m}_0/\mathrm{SF}$, the per-impulse failure probability is

$$P_{\mathrm{fail}}(f) = P\big(\mathrm{Poisson}(\bar{m}(f)) < m_{\mathrm{crit}}\big) \simeq \Psi\!\left(\frac{m_{\mathrm{crit}} - \bar{m}(f)}{\sqrt{\bar{m}(f)}}\right). \tag{S29}$$

At $f = \theta$ we have $\bar{m} = m_{\mathrm{crit}}$, so the argument vanishes and $P_{\mathrm{fail}} = 1/2$: the quantal construction *reproduces* the threshold rather than assuming it. Differentiating (S29) at that point, with $d\bar{m}/df = -\bar{m}_0 N_S \mathrm{SF}^{-(N_S-1)/N_S}$ at $\beta = 0$,

$$\left.\frac{dP_{\mathrm{fail}}}{df}\right|_{f=\theta} = \varphi(0)\, N_S\, \sqrt{\bar{m}_0\,\mathrm{SF}}\; \mathrm{SF}^{1/N_S - 1}. \tag{S30}$$

Matching this slope to that of (S28) at $f = \theta$, where $dS^{\mathrm{gr}}/df = \gamma/(4\theta)$, gives

$$\gamma_{\mathrm{q}} \simeq 4\,\theta\,\varphi(0)\, N_S\, \sqrt{\bar{m}_0\,\mathrm{SF}}\; \mathrm{SF}^{1/N_S - 1}. \tag{S31}$$

Supplementary Equation (S31) is the $\beta = 0$ case; (S23) of Supplementary Materials S1.14 gives the general form, and the reference value $\gamma_{\mathrm{q}} = 5.64$ below is to be read as conditional on that choice.

Supplementary Table S6(b) compares (S31) against exact Poisson computation of (S29). Agreement is within 6.5% at $\mathrm{SF} = 3.33$ and degrades to 11% at $\mathrm{SF} = 2$, $\bar{m}_0 = 20$, where the Gaussian step used in (S29) is least accurate; wherever a number is reported we use the exact value and not the closed form. The transition midpoint returns $f_{50} = 0.67$–0.70 at $\mathrm{SF} = 3.33$ and 0.49–0.52 at $\mathrm{SF} = 2$, in each case recovering μ_θ rather than assuming it: the property survives the human reparameterisation and was not built in.

The exact computation is a step function of $\bar{m}_0$. Transmission fails when $m < m_{\mathrm{crit}} = \bar{m}_0/\mathrm{SF}$ with m integer, so the operative threshold is $\lfloor m_{\mathrm{crit}} \rfloor$ and the fractional part of m_{crit} is discarded. The exact γ is therefore neither smooth nor monotone in $\bar{m}_0$: the ratio of the exact value to the closed form alternates between 1.127 where m_{crit} is integral and 1.051 where it is half-integral, and over $\bar{m}_0 = 19$–31 at $\mathrm{SF} = 2$ the exact γ reverses direction five times. The reference $\bar{m}_0 = 25$ gives $m_{\mathrm{crit}} = 12.5$ and falls on the lower branch; the smooth trend through the alternation would give $\gamma = 6.31$ and $W = 0.891$ rather than 5.93 and 0.922.

The alternation is a consequence of holding quantal amplitude fixed. Quantal size at the neuromuscular junction is itself variable, and any such variability convolves the lattice

away, so the smooth branch is the better estimate and the adopted value is conservative in γ and therefore anticonservative in W by about three percent. We report the reference at $\bar{m}_0 = 25$ for continuity with Supplementary Table S6, and record the excursion over the measured range of quantal content in Table 2 rather than claiming three-figure precision. Modelling quantal amplitude as a random variable would remove the alternation and is the natural next refinement of (S29); it is not attempted here.

Consequence: the binary idealisation is not adequate, and we report the correction. At the human reference (SF $= 2$, $\bar{m}_0 \approx 25$), (S31) gives $\gamma_q = 5.64$ against an exact Poisson value of 5.93, far below the $\gamma \gtrsim 20$ required for the binary approximation to hold within 10%. Recomputing the transition width at $\gamma = 5.93$ therefore inflates the sharpness constant from $\mathcal{K}_{\rm bin} = 0.2279$ to $\mathcal{K}_q = 0.3779$, an increase of 65.8% (Supplementary Table S19). The binary idealisation is thus less adequate at the human reference than at the rodent one, where the same computation gives $\gamma_q = 11.2$ and an inflation of 34.2% (Supplementary Table S6(c)): the narrower threshold law makes the step sharper, so smoothing it costs more. We report the corrected value rather than the idealised one, and all binary-limit widths in the main text are to be read as lower bounds. Under reading (ii) of Supplementary Materials S1.16 the same correction applies to the rescaled constant, since (S31) depends on θ and $\bar{m}_0$ but not on ρ.

But the scaling law is untouched, and this is verified rather than asserted. Formally, replacing S_i by $S_i^{\rm gr}$ replaces $p(C)$ by $p_\gamma(C) = E[S^{\rm gr} \mid C] \in [0,1]$, which is again smooth and strictly increasing, so the proof of Proposition 1 applies verbatim with p'_γ in place of p': only the prefactor changes. Supplementary Table S6(c) confirms this numerically at the rodent parameterisation, where the product $\Delta\bar{C}_{0.75}\sqrt{N}$ is constant at 0.628 under the graded response and the ratio to the binary case converges to 1.342, varying by under 0.2% across the range (the corresponding values at the human reference are 0.378 and 1.658). The $N^{-1/2}$ dependence originates in the binomial integration step and not in the unit response, so the falsifiable content of the sharpness law survives the removal of the binary idealisation intact.

A prediction that follows. Supplementary Equation (S31) gives $\gamma_q \propto \sqrt{\bar{m}_0}$, so junctions with higher quantal content produce sharper dose–response transitions at equal N. Population size and per-junction reliability are therefore separable sources of sharpness, and no mapping of mean concentration alone distinguishes them.

S1.21 The graded response: Bernoulli structure, width inflation, and the reading of $S^{\rm gr}$

Conditional on the field the unit states therefore remain Bernoulli, $N\Phi$ remains binomial with success probability $p_\gamma(\bar{C}) = E[S^{\rm gr} \mid \bar{C}]$, and the per-unit variance at the critical point is $\Theta_{\rm sys}(1 - \Theta_{\rm sys})$ as in the binary case.

Proposition 1 therefore applies with p'_γ in place of p' and with the prefactor unchanged in form. We state this explicitly because the alternative reading changes the answer: see Remark 2 below.

The requirement is demanding: relative to the binary limit ($\Delta\bar{C}_{0.75} = 0.002279$ at $N = 10^4$), the graded width is inflated by 200% at $\gamma = 3$, 66% at the reference $\gamma = 5.93$, 27% at $\gamma = 10$, 20% at $\gamma = 12$, and falls within 10% only for $\gamma \gtrsim 20$. Rather than leave γ unspecified, we derive it from the same quantal argument used in Supplementary Materials S1.12.

Remark 2 (The prefactor depends on how $S^{\rm gr}$ is read; the exponent does not)**.** *If $S_i^{\rm gr}$ were instead read as a graded activity level—each unit contributing a fractional loss of release rather than failing outright—then Φ would be a mean of bounded non-Bernoulli variables and the per-unit variance at $\bar{C}_{\rm crit}$ would be* $\mathrm{Var}(S^{\rm gr}) = 0.065$ *rather than* $\Theta_{\rm sys}(1 - \Theta_{\rm sys}) = 0.240$*. The sharpness*

Supplementary Table S6. Quantal grounding of θ and γ. **(a)** The trade-off surface of (S18), evaluated at $\theta = 0.70$: entries are the safety factor a given (N_S, β) would require. Bold entries fall inside the measured range 3–5. The table is read as an exclusion, not a determination—it rules out $N_S \geq 2$ at any β, but leaves the location of θ along the $N_S = 1$ line open, which is why Supplementary Materials S1.16 is needed. **(b)** Response steepness from quantal fluctuation: exact Poisson computation of (S29) against the closed form (S31), with the recovered transition midpoint f_{50} shown to confirm that μ_θ is reproduced and not assumed. **(c)** Effect on the sharpness law: the prefactor inflates by 34.2%, while $\Delta\bar{C}_{0.75}\sqrt{N}$ remains constant in N and the binary-to-graded ratio varies by under 0.2% across the range, confirming that γ rescales $\mathcal{K}$ and nothing else. Widths in (c) are exact binomial inversions at the rodent parameterisation and are therefore not directly comparable with Supplementary Table S12, which is computed at the human reference.

(a) Required safety factor at $\mu_\theta = 0.70$

	$\beta = 0$	$\beta = 0.25$	$\beta = 0.50$	$\beta = 1.0$
$N_S = 1$	**3.33**	**3.92**	**4.50**	5.67
$N_S = 2$	11.11	15.34	20.25	32.11
$N_S = 3$	37.04	—	—	—

(b) Response steepness from quantal fluctuation

Convention: $P_{\text{fail}}(f) = P\big(\text{Poisson}(\bar{m}_0(1-f)^{N_S}) < m_{\text{crit}}\big)$ evaluated exactly; f_{10}, f_{50}, f_{90} by bisection; $\gamma_{\text{exact}} = 4\ln(3)\, f_{50}/(f_{90} - f_{10})$, the slope of the logistic (S28) matched at f_{50}. The rodent block uses SF = 3.33 as written and not 10/3: because m_{crit} enters through $\lceil\cdot\rceil$, the two differ where $\bar{m}_0$/SF crosses an integer, by up to 16% in γ at $\bar{m}_0 = 20$. This is the same lattice effect as Supplementary Materials S1.20 and is the reason γ is not quoted to three figures.

SF	$\bar{m}_0$	m_{crit}	f_{50}	10–90% width in f	γ exact	γ from (S31)
3.33	20	6.01	0.6665	0.3319	8.83	9.11
**3.33**	**30**	**9.01**	**0.6777**	**0.2662**	**11.19**	**11.16**
3.33	50	15.02	0.6866	0.2031	14.85	14.41
3.33	100	30.03	0.6933	0.1420	21.45	20.38
3.33	300	90.09	0.6978	0.0814	37.68	35.29
2.00	20	10.00	0.5166	0.3992	5.69	5.05
2.00	25	12.50	0.4933	0.3654	5.93	5.64
2.00	30	15.00	0.5111	0.3276	6.86	6.18
2.00	100	50.00	0.5033	0.1807	12.24	11.28

(c) Effect on the sharpness law ($\gamma = 11.2$, i.e. $\bar{m}_0 \approx 30$)

N	$\Delta\bar{C}_{0.75}$ binary	$\Delta\bar{C}_{0.75}$ graded	$\sqrt{N}\times$ binary	$\sqrt{N}\times$ graded	ratio
200	0.032987	0.044171	0.46651	0.62467	1.3391
1,000	0.014794	0.019838	0.46781	0.62734	1.3410
5,000	0.006620	0.008879	0.46808	0.62787	1.3414
10,000	0.004681	0.006279	0.46811	0.62794	1.3415
50,000	0.002094	0.002808	0.46813	0.62799	1.3415

constant would then be $\mathcal{K} = 0.197$ rather than 0.378: the smoothed response would narrow the transition relative to the binary limit rather than widen it, and the binary widths of Supplementary Table S12 would be upper rather than lower bounds. The $N^{-1/2}$ exponent is identical under both readings, and the product $\Delta\bar{C}_q\sqrt{N}$ is constant in N in both. This is a further instance of the paper's general position: the exponent is the transportable content, and the prefactor is conditional on modelling choices that Table 2 states as maps. The reading of S^{gr} is not among them, because it is a choice of what the model represents rather than a parameter to be measured; we adopt the silencing-probability reading and report the alternative constant here.

S1.22 Gradedness of the collective readout

Since Φ is asymptotically normal with standard deviation $\sqrt{\Theta_{\mathrm{sys}}(1-\Theta_{\mathrm{sys}})/N}$ at the operating point, $P(Y{=}1 \mid \bar{C}) = E[G(\Phi)]$ is a convolution and the widths add in quadrature:

$$\Delta\bar{C}_q^2 = \frac{\mathcal{K}^2}{N} + \frac{w_G^2}{p'(\bar{C}_{\mathrm{crit}})^2}, \tag{S32}$$

with w_G the interquantile width of G. The $N^{-1/2}$ term survives intact; what the smooth readout adds is an N-independent term, of the same form and the same consequence as the between-subject dispersion of Equation (23).

The idealisation is therefore adequate when $w_G/p' \ll \mathcal{K}/\sqrt{N}$, and that condition is met precisely where the paper needs it. At $N = 20$–100 the finite-size band spans 20–49% of the critical dose (Supplementary Table S13) and any plausible endpoint satisfies it; at $N = 10^4$ the band is 0.92% and no clinical readout does. The regime in which the collective threshold may be treated as sharp is the small-N regime Section 6 identifies as operative, and the regime in which it may not is the one Section 4.8 already excludes from testing on other grounds. A readout of unknown gradedness is thus not a separate limitation: it is the third contaminant of Section 4.8, entering through the same channel and bounded by the same requirement that endpoints be matched across the targets compared.

S1.23 Units, and what the reference values do and do not fix

The dispersion does not follow from the same step, and the choice is worth stating because it is not forced. Holding $\mathrm{CV}(\theta) = 0.15$ fixed gives $\sigma_\theta = 0.075$; transporting $\mathrm{CV}(\mathrm{SF}) = 0.35$ through Supplementary Materials S1.12 would instead give 0.175. We adopt the former on two grounds. The evidence constrains the safety factor and says nothing about its dispersion, so the minimal change is to leave the dispersion at its declared value. And the latter is excluded by the model itself: under $\sigma_\theta = 0.175$ the implied distribution of $\mathrm{SF}_i = 1/(1-\theta_i)$ places its fifth percentile at 1.27, so one junction in twenty would operate at a margin under 30% and a 30% quantal run-down would fail 14% of junctions, against 0.31% at $\sigma_\theta = 0.075$. That is incompatible with the absence of transmission failure at maximal voluntary exertion [27]. The argument uses the SF $\leftrightarrow \theta$ map at $\beta = 0$ and would change at $\beta > 0$, and it yields a prediction in its own right: the model requires $\mathrm{CV}(\mathrm{SF}) \lesssim 0.15$ in human muscle.

Two properties survive the reparameterisation and were not built in. The quantal construction still *reproduces* the threshold rather than assuming it, returning $f_{50} = 0.49$–0.52 against $\mu_\theta = 0.50$. And the $N^{-1/2}$ exponent is unchanged. What moves is every constant of scale: $\bar{C}_{\mathrm{crit}}$ from 0.7161 to 0.4096 and $\mathcal{K}$ from 0.6280 to 0.3779, while the dimensionless quantities barely move—W from 0.8769 to 0.9226 and the Hill coefficient from 2.506 to 2.382. That contrast is the clearest demonstration this paper can offer of its own position: a species-specific substitution displaces what depends on scale and leaves the falsifiable content where it was.

https://doi.org/10.3390/toxins18090403

The condition is properly stated in terms of the expected count at the operating point rather than in terms of λ alone. What must be large is $E[K] = \lambda\bar{C}_{crit}$, and what must be small is the change in cleaved fraction produced by a single event near the threshold, $\eta(1-\theta)$, relative to the threshold dispersion σ_θ. At the reference configuration $E[K] = 14.3$, $\mathrm{CV}(K) = 0.26$ and $\eta(1-\theta)/\sigma_\theta = 0.38$: the threshold law is finely sampled and the mean-field reduction is accurate to 5.4%. The degenerate corner is $E[K] \lesssim 2$, where a single entry nearly suffices and θ_i ceases to do any work. We report the mean-field expressions as accurate under $E[K] \gtrsim 10$ and state that condition explicitly, because it is a condition on the operating point and not on λ: a parameterisation with $\lambda = 35$ but a tenfold smaller $\bar{C}_{crit}$ sits in the degenerate corner despite the same λ.

Concentrations are expressed in reference units in which $\kappa = 2.0$; that is, $\bar{C} = 1$ denotes the concentration at which the mean-field cleaved fraction reaches $1 - e^{-2} = 0.865$. We are explicit that this is an internal normalisation and *not* a clinical unit: converting $\bar{C}_{crit} = 0.4096$ into units of onabotulinumtoxinA per injection site would require a tissue-specific diffusion and potency calibration that this model does not contain, and no such conversion is made anywhere in this work. This is also why doses in the geometry and uptake results are expressed as multiples of $\bar{C}_{crit}$ rather than in absolute reference units: so expressed they are nearly invariant between the two parameterisations, whereas in absolute units they are not. Numerical values are reported to four decimals because the computation is deterministic given the parameters, not because that precision is claimed to be physically meaningful.

Correspondingly, $\lambda = 35$ means thirty-five productive translocation events per unit *per reference concentration unit*. What the reference value fixes is the ratio $\mathrm{CV}(K) = (\lambda\bar{C})^{-1/2}$ that controls departure from mean-field behaviour; what it does not fix is any absolute molecular count. The quantity needed to close that gap—the number of light-chain molecules reaching the cytosol in catalytically competent form per terminal at a clinical dose—has, to our knowledge, not been measured.

Supplementary Table S7. Reference parameter set and the widest range over which each parameter was assessed. These are the ranges the joint sampling of Supplementary Figure S5 draws from; the one-factor-at-a-time excursions of Table 2 of the main text and of Supplementary Table S21 are computed over narrower ranges for σ_θ and γ, stated and justified there. The human configuration is primary; the rodent configuration is retained for comparison with the earlier parameterisation. The status of each parameter is stated explicitly: two are now derived rather than assumed.

Symbol	Meaning	Human	Rodent	Range assessed	Status
κ	uptake–cleavage composite	2.0	2.0	1.0–4.0	assumed
λ	productive events per unit	35	20	1–500	assumed (Supplementary Materials S1.3)
η	per-event cleavage yield	0.0571	0.1000	κ/λ	derived
μ_θ	mean unit threshold	0.50	0.70	0.40–0.60	**derived** from SF at β=0 (Supplementary Materials S1.14)
σ_θ	threshold dispersion	0.075	0.105	0.05–0.175	from $\mathrm{CV}(\theta) = 0.15$
Θ_{sys}	collective threshold	0.60	0.60	0.50–0.70	assumed
γ	response steepness	5.93	11.2	2–100	**derived** from SF, $\bar{m}_0$ at β=0
N	independent units	10,000	10,000	20–50,000	assumed (Supplementary Materials S1.7)
k	endplates per fibre	1	1	1–5	varied (Supplementary Materials S1.9)
s/R	dispersion radius	—	—	0.15–∞	varied
	threshold law	$\mathrm{Beta}(21.72, 21.72)$ / $\mathrm{Beta}(12.63, 5.41)$			derived

Supplementary Materials S2. Proofs

Throughout this appendix $p(\cdot)$ denotes either p^{SU} or p^{MF}; all results require only that p be continuously differentiable and strictly increasing in a neighbourhood of $\bar{C}_{\mathrm{crit}}$, where $\bar{C}_{\mathrm{crit}}$ is defined by $p(\bar{C}_{\mathrm{crit}}) = \Theta_{\mathrm{sys}}$.

S2.1 The comparison class, and two claims withdrawn

All claims are judged against the stochastic mean-only benchmark

$$Y^{\mathrm{B}} \sim \mathrm{Bernoulli}\big(g(\bar{C})\big), \tag{S33}$$

with g any measurable function of the spatial mean. This is deliberately stronger than the deterministic benchmark $R = g(\bar{C})$ used in earlier comparisons, for the reason made formal in Lemma 1.

Lemma 1 (The benchmark reproduces the entire variance profile)**.** *Let g be calibrated so that $g(\bar{C}) = P(Y{=}1 \mid \bar{C})$ for all $\bar{C}$. Then $\mathrm{Var}(Y^{\mathrm{B}} \mid \bar{C}) = \mathrm{Var}(Y \mid \bar{C})$ for all $\bar{C}$; in particular the benchmark attains the same variance maximum, at the same concentration, with no additional freedom.*

Proof. Both Y and Y^{B} are $\{0,1\}$-valued. For any Bernoulli variable Z with mean μ we have $\mathrm{Var}(Z) = \mu(1-\mu)$: the variance is a deterministic function of the mean. Calibrating the mean therefore calibrates the variance pointwise, so the mean curve, the variance curve, the location of the variance maximum and its value $1/4$ are all reproduced by construction. □

Lemma 1 has an immediate and unwelcome consequence for two claims that a model of this class might appear to support. We record them as observations rather than results.

Remark 3 (Observation 1: the variance peak is not evidence)**.** *Since $Y = \mathbb{I}(\Phi \geq \Theta_{\mathrm{sys}})$ is binary, $\mathrm{Var}(Y) = P(Y{=}1)\,[1 - P(Y{=}1)]$, maximised at $1/4$ when $P(Y{=}1) = 1/2$, which occurs at $\bar{C} = \bar{C}_{\mathrm{crit}}$. This is an identity of the Bernoulli distribution. By Lemma 1 it holds identically for the mean-only benchmark and therefore discriminates nothing. Describing it as a critical transition, a phase transition, or an amplification cascade imports statistical-physics vocabulary onto an arithmetic fact. The informative quantity is the* width *of the peak, not its height, which is the content of Proposition 1.*

Remark 4 (Observation 2: mean-curve agreement is not evidence)**.** *For any target curve there exists a g reproducing it exactly, namely $g = P(Y{=}1 \mid \cdot)$ itself. Agreement between the discrete model and a calibrated Hill curve on the uniform-field dose–response therefore carries no information about architecture, and is not presented as validation anywhere in this work.*

Remark 5 (Claims withdrawn)**.** *Earlier formulations of this framework asserted structural non-equivalence to mean-field models on three counts: the mean curve, the variance peak, and the geometry divergence. Against the benchmark (S33) the first two do not survive, by Observations 3 and 4, and are withdrawn. What survives is the geometry claim (Proposition 2) and, as a positive quantitative statement rather than an impossibility result, the sharpness law (Proposition 1). A third claim is withdrawn in Supplementary Materials S3.1—that the cleavage–effect correlation coefficient discriminates between architectures—and a fourth entry records a superseded derivation rather than a withdrawn claim; Supplementary Table S8 collects all four.*

S2.2 The conditions the sharpness law actually requires

The conditions the result actually requires. It follows that the architecture is not specific to botulinum toxin, and it is worth stating what it does require, because the list is shorter

than the model as presented. Proposition 1 uses three properties and no others: a population of units that is finite and whose members fail independently, or with a correlation that reduces the count by a bounded factor (Supplementary Materials S1.8); a per-unit threshold with dispersion, whatever the underlying mechanism of failure; and a readout that is a threshold on the fraction of units silenced rather than on which units they are. Nothing in the derivation uses cleavage, uptake, or any property of a neurotoxin. Those enter only in fixing the constants—the terminal threshold from quantal transmission, the steepness from quantal content—and in making them measurable.

We instantiate the architecture on upper-face BoNT-A because that is where the three quantities can be anchored to published measurement: the safety factor of neuromuscular transmission fixes the threshold, quantal content fixes the steepness, and motor-unit number estimation makes the unit count accessible. An intervention that silences discrete units by another route, and is judged by a collective functional endpoint, would satisfy the same three conditions and inherit the same exponent, with different constants. We do not develop that here, and note only that the transportable content of this paper is the architecture rather than the pharmacology. What is new here is the identification of the observable consequence in this system: that the relation transports between injection targets with no free parameters, that it is measurable in administered dose through Corollary 3.7, and that it makes threshold dispersion estimable. A hierarchical model $g(\bar{C}, N)$ reproducing the same relation would not be a competitor to this architecture; it would be a reparameterisation of it.

Supplementary Table S8. Four statements that a discrete threshold architecture might appear to support and does not. Each fails against the stochastic mean-only benchmark of (S33) or against its own supporting computation. The first three are claims, and none of them is recoverable by a future measurement: they are excluded rather than conditional. The fourth is a derivation rather than a claim—the conclusion it reached is retained, but the route by which our first revision reached it is inadmissible—and it is collected here because a reader comparing the two revisions would otherwise find the same ordering supported by two incompatible arguments. This appendix records the first two formally, Supplementary Materials S3.1 the third and Supplementary Materials S3.2 the fourth.

Claim not advanced	Why it does not survive	Where
The output-variance maximum at 1/4 is a signature of the architecture	An identity of the Bernoulli distribution, holding for the mean-only benchmark identically once its mean is calibrated	Obs. 1; this appendix
Agreement with a calibrated mean-field curve is evidence of non-equivalence	g can be calibrated to reproduce any target curve by construction, so the agreement carries no information about architecture	Obs. 2; this appendix
The cleavage–effect correlation coefficient discriminates between architectures	Both quantities are monotone in dose, so they correlate; what falls as $N^{-1/2}$ is the informative band, not the correlation, which is set by the assay's own dispersion	Section 4.5, Eq. (21)
The serotype ordering follows from a serotype-specific logistic steepness γ	γ is a function of quantal content and the safety factor, not a free parameter, and β lowers it rather than raising it; $\gamma = 12$ is the superseded rodent value and is unattainable at SF $= 2$. The ordering is retained but re-derived through β, in points of measured cleavage	Supplementary Materials S3.2, Supplementary Table S4

S2.3 *Why the mean-only benchmark is stochastic*

The first is the stochastic mean-only mapping

$$Y^B \sim \text{Bernoulli}(g(\bar{C})), \tag{S34}$$

with g any measurable function of the spatial mean concentration, calibrated to the discrete model's mean curve. This is a stronger benchmark than the deterministic mapping $R = g(\bar{C})$ that such comparisons usually adopt: a deterministic benchmark has zero variance everywhere, so any stochastic model appears to beat it on variance and the resulting non-equivalence claim is vacuous. Because Equation (S34) is Bernoulli, its variance $g(1-g)$ is a deterministic function of its mean, so calibrating the mean calibrates the entire variance profile at no extra cost. Its role here is negative: it establishes what a mapping without a unit count cannot express.

S2.4 Prior art, and two qualifications on geometry non-sufficiency

The mathematics is standard. The derivation is the delta method applied to a binomial quantile, and the underlying result—that the threshold width of a monotone symmetric property on N independent coordinates contracts as $N^{-1/2}$ in the extremal case—is established in probability theory [41–43]. The architecture itself is the k-out-of-N system of reliability theory, and the exact binomial tail we use is the m-out-of-n fusion rule of distributed detection [44]. In population coding the $\sqrt{N}$ gain of an independent population has been standard since [45]. We claim no novelty for any of this.

The architecture is prior in pharmacodynamics. The critical subset model of antiviral action posits a finite population of independent drug targets and a threshold number that must remain functional, states that the slope of the dose–response curve is determined by that population and that threshold, and validates the dependence experimentally by modulating the target count [40,46]. Separately, [47] established that the apparent Hill exponent is the product of effector-unit heterogeneity and subunit shape, that heterogeneity always lowers it, and that no mechanistic interpretation follows from its value—the last of which we adopt rather than rediscover. Attempts to read a unit count off the slope of a concentration–response curve have a longer history in receptor pharmacology and in ion-channel gating [48,49]; the caution [47] states applies to them, and it applies here too, which is why the claim of this paper is the constancy of $n_{\mathrm{eff}}/\sqrt{N}$ across targets and not the value of the coefficient.

Two qualifications keep this honest. First, the effect is not specific to binary terminal responses: a spatially resolved smooth model $\Phi_{\mathrm{SR}} = N^{-1}\sum_i h(C_i)$ also produces it, because $\langle h(C_i)\rangle \neq h(\langle C_i\rangle)$ whenever h is nonlinear. Non-sufficiency of the spatial mean is in that sense an instance of Jensen's inequality, and we claim it as an observation rather than as a result of this architecture; what the architecture supplies is the critical radius s^* and its interior optimum in placement, both conditional on the field family. What Proposition 2 excludes is the mean-only class, (S34), which is nonetheless the class in routine pharmacodynamic use. Second, the magnitude depends on the field family, for which Supplementary Materials S1.18 gives the reason s^* is reported.

Such formulations treat the local concentration as a sufficient statistic for terminal behaviour, assuming that all terminals within a concentration zone respond equivalently [69]. This neglects the discreteness of molecular binding events and the threshold-dependent nature of individual terminal responses [14,15], the spatial heterogeneity of toxin distribution [16,53], and the transition from single-terminal behaviour to emergent collective dynamics [17–19,25].

https://doi.org/10.3390/toxins18090403

S2.5 Proof of Proposition 1 (sharpness law)

Proposition 1 (Sharpness law). *Let p be continuously differentiable and strictly increasing near $\bar{C}_{\mathrm{crit}}$ with $p'(\bar{C}_{\mathrm{crit}}) > 0$, and for $q \in (1/2, 1)$ let $\bar{C}_q$ solve $P(Y{=}1 \mid \bar{C}_q) = q$. Set $\Delta\bar{C}_q := \bar{C}_q - \bar{C}_{1-q}$. Then*

$$\lim_{N\to\infty} \Delta\bar{C}_q \sqrt{N} = \frac{2\, z_q \sqrt{\Theta_{\mathrm{sys}}(1-\Theta_{\mathrm{sys}})}}{p'(\bar{C}_{\mathrm{crit}})} =: \mathcal{K}, \tag{S35}$$

where $z_q = \Psi^{-1}(q)$ and Ψ is the standard normal CDF. The limit $\mathcal{K}$ is independent of N.

Proof. Under a uniform field, (A4) gives $N\Phi \sim \mathrm{Binomial}(N, p(\bar{C}))$, so

$$P(Y{=}1 \mid \bar{C}) = P\big(N\Phi \geq \lceil \Theta_{\mathrm{sys}} N \rceil\big). \tag{S36}$$

Write $p = p(\bar{C})$, $\sigma^2 = p(1-p)$, and let $\rho = E|S_i - p|^3 = p(1-p)\,[p^2 + (1-p)^2]$ be the third absolute central moment of a Bernoulli variable. By the Berry–Esseen theorem, for all x,

$$\left| P\!\left(\frac{N\Phi - Np}{\sigma\sqrt{N}} \leq x\right) - \Psi(x) \right| \leq \frac{C_{\mathrm{BE}}\,\rho}{\sigma^3\sqrt{N}}, \qquad C_{\mathrm{BE}} \leq 0.4748. \tag{S37}$$

Applying (S37) to (S36),

$$P(Y{=}1 \mid \bar{C}) = \Psi\!\left(\frac{\sqrt{N}\,[\,p(\bar{C}) - \Theta_{\mathrm{sys}}\,]}{\sqrt{p(1-p)}}\right) + O(N^{-1/2}). \tag{S38}$$

Two approximation terms are absorbed into the remainder, and both are $O(N^{-1/2})$: the Berry–Esseen term of (S37), and the lattice correction arising because $N\Phi$ is integer-valued, so that $\lceil \Theta_{\mathrm{sys}} N \rceil - \Theta_{\mathrm{sys}} N \in [0, 1)$ perturbs the argument of Ψ by at most $1/(\sigma\sqrt{N})$.

Setting $P(Y{=}1 \mid \bar{C}_q) = q$ and inverting (S38),

$$p(\bar{C}_q) = \Theta_{\mathrm{sys}} + \frac{z_q \sqrt{\Theta_{\mathrm{sys}}(1-\Theta_{\mathrm{sys}})}}{\sqrt{N}} + o(N^{-1/2}), \tag{S39}$$

where $p(1-p)$ has been evaluated at $p = \Theta_{\mathrm{sys}}$; this is legitimate because $p(\bar{C}_q) \to \Theta_{\mathrm{sys}}$ as $N \to \infty$ and $p \mapsto p(1-p)$ is continuous. Since p is continuously differentiable with $p'(\bar{C}_{\mathrm{crit}}) > 0$, it is locally invertible near $\bar{C}_{\mathrm{crit}}$, and a first-order expansion of p^{-1} about Θ_{sys} gives

$$\bar{C}_q = \bar{C}_{\mathrm{crit}} + \frac{z_q \sqrt{\Theta_{\mathrm{sys}}(1-\Theta_{\mathrm{sys}})}}{p'(\bar{C}_{\mathrm{crit}})\sqrt{N}} + o(N^{-1/2}). \tag{S40}$$

Applying (S40) at q and at $1-q$, using $z_{1-q} = -z_q$, and subtracting yields $\Delta\bar{C}_q = 2z_q\sqrt{\Theta_{\mathrm{sys}}(1-\Theta_{\mathrm{sys}})}\,/\,[p'(\bar{C}_{\mathrm{crit}})\sqrt{N}] + o(N^{-1/2})$. Multiplying by $\sqrt{N}$ and letting $N \to \infty$ gives (S35). □

Corollary 1 (Order of the approximation, and a rigorous bound). *Let ε_N bound the uniform error of the normal approximation to $P(Y{=}1 \mid \bar{C})$, comprising the Berry–Esseen term of* (S37) *and the lattice offset $\lceil \Theta_{\mathrm{sys}} N \rceil - \Theta_{\mathrm{sys}} N \in [0, 1)$. Then, to first order in ε_N,*

$$\frac{\big|\Delta\bar{C}_q - \mathcal{K}/\sqrt{N}\big|}{\Delta\bar{C}_q} \leq \frac{\varepsilon_N}{z_q\, \varphi(z_q)}, \tag{S41}$$

with φ the standard normal density. Moreover the true error is of smaller order than this bound:

$$\Delta\bar{C}_q\sqrt{N} \;=\; \mathcal{K}\Big(1-\frac{c_1}{N}+o(N^{-1})\Big). \tag{S42}$$

Proof. For (S41), write $P(Y{=}1 \mid \bar{C}) = \Psi(x(\bar{C})) + R(\bar{C})$ with $|R| \le \varepsilon_N$ and $x(\bar{C}) = \sqrt{N}\,[p(\bar{C})-\Theta_{\text{sys}}]/\sqrt{p(1-p)}$. Solving $P(Y{=}1 \mid \bar{C}_q) = q$ gives $x(\bar{C}_q) = \Psi^{-1}(q-R)$, so $|x(\bar{C}_q) - z_q| \le \varepsilon_N/\varphi(z_q) + O(\varepsilon_N^2)$. Since $dx/d\bar{C} = \sqrt{N}\,p'(\bar{C}_{\text{crit}})/\sqrt{\Theta_{\text{sys}}(1-\Theta_{\text{sys}})} + o(\sqrt{N})$ near $\bar{C}_{\text{crit}}$, the displacement in concentration is at most $\varepsilon_N\sqrt{\Theta_{\text{sys}}(1-\Theta_{\text{sys}})}/[\varphi(z_q)\,p'(\bar{C}_{\text{crit}})\sqrt{N}]$ per quantile. Doubling this and dividing by $\Delta\bar{C}_q = 2z_q\sqrt{\Theta_{\text{sys}}(1-\Theta_{\text{sys}})}/[p'(\bar{C}_{\text{crit}})\sqrt{N}]$ gives (S41).

For (S42), note that the bound just derived is uniform in sign, whereas the leading corrections are not. The Edgeworth skewness term and the lattice offset both displace x by the same amount at q and at $1-q$, because each depends on N and on $p(\bar{C}_{\text{crit}}) = \Theta_{\text{sys}}$ but not on q at leading order. Each quantile is therefore displaced by $O(N^{-1})$ in concentration—the $O(N^{-1/2})$ error in probability divided by the $O(\sqrt{N})$ slope $dx/d\bar{C}$—with a common leading coefficient, and the difference $\bar{C}_q - \bar{C}_{1-q}$ retains only the residual, which is $O(N^{-3/2})$. Dividing by $\Delta\bar{C}_q = O(N^{-1/2})$ gives a relative error $O(N^{-1})$. □

Supplementary Table S9 confirms all three orders numerically. The bound (S41) is $O(N^{-1/2})$ while the error is $O(N^{-1})$, so the ratio between them grows as $\sqrt{N}$; this is a property of a uniform worst-case bound and not an indication that either quantity is wrong.

Supplementary Table S9. Orders of convergence and the rigorous bound. Column 2 is the displacement of a single quantile relative to the closed form, scaled by N; column 3 is the displacement of the width, scaled by $N^{3/2}$; column 4 is the relative width error scaled by N, which is the constant c_1 of (S42). Columns 5–6 give the bound (S41) with and without the lattice term. Quantiles were obtained by inversion to a tolerance of 10^{-15}; the production solver of Supplementary Materials S5.2 works to 10^{-9} and does not resolve the residual beyond $N \approx 10^4$. Computed at the rodent parameterisation, retained here because these are verifications of Proposition 1 rather than parametric results; the proposition and its proof are unchanged at the human reference.

N	$N\,\delta\bar{C}_q$	$N^{3/2}\,\delta(\Delta\bar{C}_q)$	c_1	bound (BE)	bound (BE + lattice)	observed
200	−0.3606	−0.3257	0.6958	16.6%	43.5%	0.348%
1,000	−0.3550	−0.3272	0.6989	7.4%	19.5%	0.070%
5,000	−0.3523	−0.3275	0.6995	3.3%	8.7%	0.014%
20,000	−0.3512	−0.3275	0.6996	1.7%	4.4%	0.003%
100,000	−0.3506	−0.3275	0.6996	0.7%	1.9%	0.001%

Supplementary Table S10. The closed form of Proposition 1 against exact binomial widths, at the human reference under the quantal response ($\bar{C}_{\text{crit}} = 0.409601$, $p'(\bar{C}_{\text{crit}}) = 1.748849$, $\mathcal{K} = 0.377884$). The relative error is c_1/N with $c_1 = 1.080$, constant to within 0.12% across more than two decades: the approximation is $O(N^{-1})$ and not $O(N^{-1/2})$ as the Berry–Esseen term alone would suggest. Supplementary Table S9 reports the same constant at the rodent parameterisation, where it is 0.700.

N	exact width	predicted width	relative error (%)	error $\times N$
200	0.02657704	0.02672044	+0.5396	1.0791
500	0.01686307	0.01689949	+0.2160	1.0799
1,000	0.01193685	0.01194974	+0.1080	1.0802
2,500	0.00755442	0.00755768	+0.0432	1.0804
5,000	0.00534293	0.00534409	+0.0216	1.0804
10,000	0.00377843	0.00377884	+0.0108	1.0804
50,000	0.00168991	0.00168995	+0.0022	1.0805

Corollary 2 (Non-transportability)**.** *Let g be a mean-only mapping calibrated exactly to the discrete dose–response at unit number N_0. Then g reproduces the transition width at N_0 by construction and predicts that same width at every other N, whereas (S35) gives width proportional to $N^{-1/2}$. The relative error of the calibrated mapping at unit number N is therefore $\sqrt{N/N_0} - 1 + o(1)$, which vanishes only at $N = N_0$.*

Remark 6 (Where the discriminating content lies)**.** *Corollary 2 is the operational form of the claim that the architecture is not redundant. It is a statement about* transport *between targets, not about fit quality on any single target, and it is falsifiable: a mean-only mapping that transported correctly across targets of differing N would contradict Proposition 1. The prediction is verified directly in Figure 2 of the main text, where the measured mispredictions of -49.8% and $+123.3\%$ differ from $\sqrt{N/N_0} - 1$ by less than a third of a percentage point, the difference being the finite-size residual of Corollary 1. Note also that (S35) depends on σ_θ through $p'(\bar{C}_{\mathrm{crit}})$, since broader threshold dispersion flattens p; this is the mechanism by which the N-ladder of Supplementary Materials S5.6 carries information about σ_θ.*

Supplementary Table S11 verifies (S35) against exact binomial computation and against direct Monte Carlo simulation, alongside the Berry–Esseen bound of (S37).

Supplementary Table S11. Verification of Proposition 1. Columns 2–3 give quantile concentrations obtained by exact binomial inversion; columns 4–5 give independent Monte Carlo estimates of $P(Y{=}1)$ at those concentrations (4,000 replicates), which should return 0.25 and 0.75. Column 6 is the scaled width, constant at $\mathcal{K} = 0.4681$ to three significant figures above $N = 1{,}000$. Column 7 is the Berry–Esseen bound (S37) at $p = \Theta_{\mathrm{sys}}$, confirming that the residual deviation at small N lies within the theoretical error of the normal approximation. Computed at the rodent parameterisation, retained here because these are verifications of Proposition 1 rather than parametric results; the proposition and its proof are unchanged at the human reference.

N	$\bar{C}_{0.25}$	$\bar{C}_{0.75}$	MC at $\bar{C}_{0.25}$	MC at $\bar{C}_{0.75}$	$\Delta\bar{C}_{0.75}\sqrt{N}$	BE bound
200	0.67765	0.71064	0.260	0.747	0.46651	0.0356
1,000	0.68814	0.70294	0.252	0.754	0.46781	0.0159
10,000	0.69351	0.69820	0.249	0.755	0.46811	0.0050

S2.6 Numerical verification of the sharpness law

The residual is not merely small but of a definite order. Multiplying the relative error by N returns 1.079, 1.080, 1.080, 1.080 and 1.080 at $N = 200$, 10^3, 5×10^3, 10^4 and 5×10^4: the approximation is $O(N^{-1})$, as Supplementary Equation (S45) states and Supplementary Table S10 verifies term by term, and not $O(N^{-1/2})$ as the Berry–Esseen term alone would suggest. Resolving this required inverting the transition to a tolerance of 10^{-15}, since at $N = 10^4$ the quantity being measured is 3×10^{-9} in concentration; the production solver, which works to 10^{-9}, cannot see it. The corresponding rigorous bound of Supplementary Materials S2 evaluates to 43.5% at $N = 200$ and 6.2% at $N = 10^4$, against observed errors of 0.54% and 0.011%. We report both, and state plainly that the gap is the price of a uniform worst-case bound rather than a defect of either quantity.

The two error terms of this paper are the same quantity, which is worth recording because they arise in different results and are verified independently. The residual c_1/N of Supplementary Equation (S45) is a statement about how fast $\Delta\bar{C}_q\sqrt{N}$ approaches its limit; the factor $\sqrt{N/N_0} - 1$ of Corollary 2 is a statement about how far a calibrated mean-only mapping misses on a target of different size (Supplementary Figure S2). The exact relation between the two is

$$1 + \varepsilon_{\mathrm{transport}} = \frac{W_{N_0}}{W_N}\sqrt{N/N_0}, \tag{S43}$$

in which the corollary is the case $W_{N_0} = W_N$. Measured on the three targets of Figure 2, the transport error departs from $\sqrt{N/N_0} - 1$ by 0.24 and 0.31 percentage points, and Supplementary Equation (S43) closes that gap to 3×10^{-3} and 1.5×10^{-2} points using the measured W. The departure from the corollary is therefore the finite-size residual itself, and neither result is approximate where the other is exact.

Supplementary Table S12. Transition width against unit number, by exact binomial inversion, at the human reference of Section 4.11. The quantal response ($\gamma_q = 5.93$) is the reported case; the binary limit is retained as a reference. Widths are expressed as a percentage of the corresponding critical concentration ($\bar{C}_{crit} = 0.4096$ for $\gamma = 5.93$, 0.3862 in the binary limit). The product $\Delta\bar{C}_{0.75}\sqrt{N}$ is constant to within 0.54% over more than two decades in N under the quantal response and 0.32% in the binary limit.

	quantal response ($\gamma = 5.93$)			binary limit (reference)		
N	$\Delta\bar{C}_{0.75}$	$\Delta\bar{C}_{0.75}\sqrt{N}$	5–95% (%)	$\Delta\bar{C}_{0.75}$	$\Delta\bar{C}_{0.75}\sqrt{N}$	5–95% (%)
200	0.026577	0.37586	15.86	0.016063	0.22717	10.15
500	0.016863	0.37707	10.05	0.010179	0.22761	6.43
1,000	0.011937	0.37748	7.11	0.007202	0.22776	4.55
2,500	0.007554	0.37772	4.50	0.004557	0.22785	2.88
5,000	0.005343	0.37780	3.18	0.003223	0.22788	2.04
10,000	0.003778	0.37784	2.25	0.002279	0.22789	1.44
50,000	0.001690	0.37788	1.01	0.001019	0.22791	0.64

Two qualifications belong here. The binary-limit columns of Supplementary Table S12 are retained for reference only, and the widths in them are lower bounds on the quantal ones that this section reports (Section 4.3), and all absolute concentrations are conditional on the assay reading of Section 2.5. Neither touches the exponent, which is what the law asserts.

Supplementary Table S13 gives the same result in the form of Corollary 3.7, computed under the quantal response ($\gamma_q = 5.93$) that Section 4.3 derives. The scale-free product converges to $W = 0.9226$, is within 1% of that value from $N = 200$ upward and within 5% even at $N = 20$, and the corresponding effective Hill exponent runs from 11 to 533 across the range. The two smallest entries are included because, once N is instantiated as an independent-unit count (Section 6), targets of a few tens of units are within range.

Supplementary Table S13. Scale-free transition width and effective Hill exponent under the quantal response at the human reference ($\gamma_q = 5.93$, $\bar{C}_{crit} = 0.4096$). The relative width is measurable in administered dose units without any concentration calibration (Corollary 3.7); its product with $\sqrt{N}$ converges to the invariant $W = \mathcal{K}/\bar{C}_{crit} = 0.9226$, and is within 1% of that value from $N = 200$ upward. n_{eff} is obtained from the exact interquartile ratio rather than from the asymptotic form (15). The last column is the 5–95% band quoted in the clinical reading.

N	$\Delta\bar{C}_{0.75}/\bar{C}_{crit}$ (%)	$W = (\Delta\bar{C}_{0.75}/\bar{C}_{crit})\sqrt{N}$	n_{eff}	5–95% band (%)
20	19.58	0.8758	11.2	48.73
50	12.77	0.9031	17.2	31.42
100	9.13	0.9127	24.1	22.36
200	6.49	0.9176	33.9	15.86
500	4.12	0.9206	53.4	10.05
1,000	2.91	0.9216	75.4	7.11
2,500	1.84	0.9222	119.1	4.50
5,000	1.30	0.9224	168.4	3.18
10,000	0.92	0.9225	238.2	2.25
50,000	0.41	0.9226	532.6	1.01

That $W = 0.9226$ is the value at the reference parameters, and it is not a central estimate. Sampling the declared ranges of Supplementary Table S7 jointly (Supplementary Materials S5, Supplementary Figure S5) rather than one factor at a time gives a median W of 1.037 with a 90% interval of $[0.775, 1.367]$: the reference value sits near the 25th percentile. Threshold dispersion alone carries half the variance of W ($S_1 = 0.502$), five times the next contributor, which reverses the ordering that the one-factor-at-a-time excursions of Table 2 suggest. The one-factor excursions also understate the joint spread by 12%, because σ_θ dominates and the factors interact.

The prefactor also depends on threshold dispersion through $p'(\bar{C}_{\text{crit}})$. Over the range plotted in Supplementary Figure S1(C), $\sigma_\theta = 0.04$–0.20 at $\mu_\theta = 0.50$ (coefficient of variation 0.08–0.40), $\mathcal{K}$ runs from 0.194 to 0.479 in the binary limit—a factor of 2.47, with the human reference at $\sigma_\theta = 0.075$ returning 0.228—while $\bar{C}_{\text{crit}}$ moves by 12.9% across the same range. Width is thus far more informative about σ_θ than location is—the basis for the recovery result of Section 4.7.

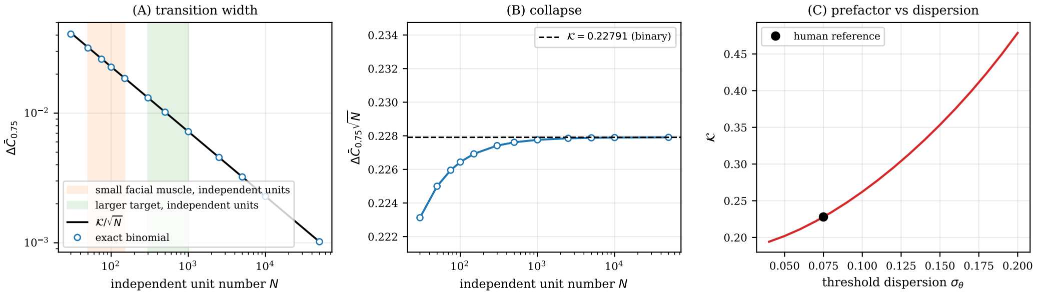

Supplementary Figure S1. Sharpness law. (A) Transition width $\Delta\bar{C}_{0.75}$ against independent-unit number N on logarithmic axes; circles are exact binomial computations, the line is Equation (12). Shaded bands mark plausible ranges for a small facial muscle ($N \approx 50$–150, bracketing the motor-unit count measured in dilator naris [52]) and for a larger target ($N \approx 300$–1,000). The bands are independent-unit counts, not terminal counts, which exceed them by the innervation ratio (Section 6). Within the small-muscle band the $\sqrt{N}$ collapse already holds to 1.3%. **(B)** The product $\Delta\bar{C}_{0.75}\sqrt{N}$, constant to within 0.32% across the range. **(C)** Dependence of the prefactor on threshold dispersion σ_θ, which runs from $\mathcal{K} = 0.194$ at $\sigma_\theta = 0.04$ to 0.479 at $\sigma_\theta = 0.20$, with the human reference at 0.075. Panels (A), (B) and (C) show the binary limit, $\mathcal{K} = 0.228$; the quantal response of Section 4.3 rescales the constant to 0.378 without altering the collapse (Supplementary Table S12).

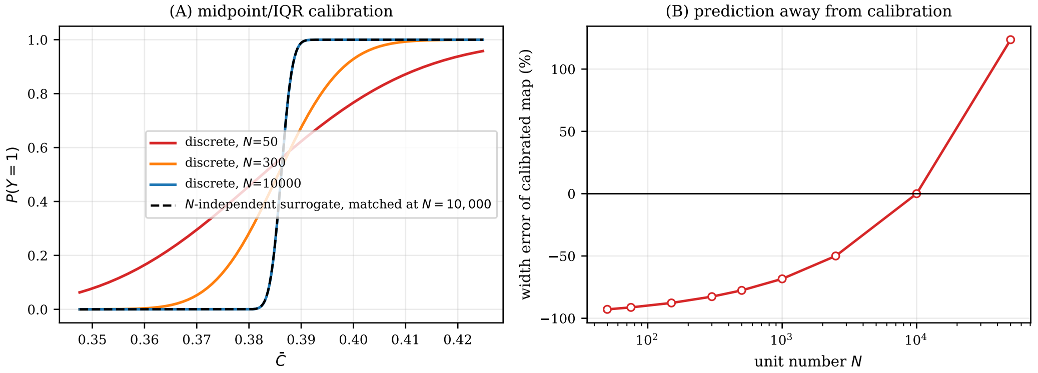

Supplementary Figure S2. Non-transportability of a mean-only mapping. (A) An N-independent surrogate matched to the discrete transition at $N = 10{,}000$ in midpoint and interquartile width (dashed), applied unchanged to targets with $N = 50$ and $N = 300$ (solid). The surrogate is matched on two summary statistics rather than fitted pointwise, so it is not an exact calibration and is not described as one. **(B)** Resulting error in predicted transition width against N; it vanishes only at the matching point and grows as $\sqrt{N/N_0} - 1$.

S2.7 The scale-free width: closed form and boundaries

In the mean-field reduction W has a closed form in which the uptake–cleavage composite cancels identically. Writing $f^* = F_\theta^{-1}(\Theta_{sys})$ and using $\bar{C}_{crit} = -\ln(1-f^*)/\kappa$ together with $p'(\bar{C}_{crit}) = f_\theta(f^*)\,\kappa\,(1-f^*)$,

$$W \;=\; \frac{2\,z_q\sqrt{\Theta_{sys}(1-\Theta_{sys})}}{f_\theta(f^*)\,(1-f^*)\,\ln[1/(1-f^*)]}, \tag{S44}$$

independent of κ.

Two boundaries keep this exact rather than approximate. The invariance holds under a gain constant across units: unit-to-unit heterogeneity in exposure or persistence enters p' and does change W, which is a distinct and testable effect rather than a hidden assumption. And dilution requires care, because it acts in two distinct ways that the model separates. Insofar as it changes the concentration delivered at fixed spatial arrangement, it is a gain and leaves W unchanged. Insofar as it changes the dispersion radius s of Equation (10), it changes the shape of the field and therefore p', and W is not invariant to it. The two are conflated in clinical usage and are kept apart here.

One convention must be fixed, because the discrete responder curve is a binomial tail and not a Hill curve, and the two differ in the tails: n_{eff} is defined here by the interquartile ratio, so a least-squares fit over a wider dose range returns a different number.

S2.8 Order of the approximation, the effective count, and the threshold density

Order of the approximation. The $O(N^{-1/2})$ error in probability does not carry over to the width. Because the quantile spacing in concentration is itself $O(N^{-1/2})$, an error ε in probability displaces a quantile by $O(\varepsilon N^{-1/2})$, and the two quantiles are displaced in the same direction by amounts that agree to leading order, so the difference gains a further half order. The result, derived in this appendix and verified numerically over three decades in N, is

$$\Delta\bar{C}_q\sqrt{N} \;=\; \mathcal{K}\Big(1 - \frac{c_1}{N} + o(N^{-1})\Big), \qquad c_1 = 1.080, \tag{S45}$$

so the closed form is accurate to first order in N^{-1} rather than $N^{-1/2}$. A rigorous but uniform bound, which cannot see the cancellation, is given in this appendix: it is $O(N^{-1/2})$ and exceeds the observed error by two to three orders of magnitude.

The impossibility is relative to a family of targets, not absolute, and the condition is worth stating exactly: it holds for a mapping whose only argument is $\bar{C}$, applied across targets whose unit counts differ and are not carried by the mapping. A mapping that took N as a second argument faces no obstruction; the content of the claim is that N must enter, and that once it does the relation between targets is fixed rather than free.

The relation is not confined to independent units. Within-unit correlation of thresholds, unequal contribution of units to the readout, and sharing of exposure within a motor-unit territory all enter through a single effective count N_{eff}, derived in Supplementary Materials S1 and verified there against direct simulation of the nested model; each rescales the prefactor and none alters the exponent. What the law asserts is the exponent, and it is robust to the three departures from strict independence that a nested architecture introduces.

More precisely, the prefactor depends on the density of the threshold law at the critical quantile, $f_\theta(f^*)$, and not on σ_θ alone. That dependence is real in the mean-field reduction, where matching only the first two moments across the Beta, log-normal and gamma families moves $\mathcal{K}$ by 9.1%. In the primary stochastic-uptake formulation it very nearly vanishes: the same comparison moves $\mathcal{K}$ by 0.14% and W by 0.25%, because Poisson uptake convolves the threshold law and the stochastic slope is no longer governed by $f_\theta(f^*)$ (Supplementary

Materials S1.15). The Beta family of (S16) is therefore adopted because it is bounded on $[0, 1]$ as the model requires, and the constants reported here are not properties of that choice. What does move the measurable width is the dispersion itself, by 35.3% over the range assessed (Table 2).

S2.9 Proof of Proposition 2 (geometry non-sufficiency)

Proposition 2 (Geometry non-sufficiency at matched mean)**.** *Consider the field family (S27) at fixed spatial mean $\bar{C}$. If p is not affine on the range of the field, then there exist dispersion radii $s_A \neq s_B$ with*

$$P(Y{=}1 \mid \bar{C}, s_A) \neq P(Y{=}1 \mid \bar{C}, s_B), \tag{S46}$$

so $\bar{C}$ is not a sufficient statistic for system output. Moreover $E[\Phi \mid \bar{C}, s]$ is non-decreasing in s, so the critical dispersion radius

$$s^*(\bar{C}) := \inf\{\, s > 0 : P(Y{=}1 \mid \bar{C}, s) \geq 1/2 \,\} \tag{S47}$$

is well defined, and block is unattainable for $s < s^*$ at that dose.

Proof. Write $E[\Phi \mid \bar{C}, s] = \langle p(C(r)) \rangle_R$ with $C(r)$ given by (S27). As $s/R \to \infty$ the field converges uniformly to the constant $\bar{C}$, so $\langle p(C(r)) \rangle_R \to p(\bar{C})$ by dominated convergence. As $s/R \to 0$ the field concentrates: for any $\epsilon > 0$ the measure of $\{r : C(r) > \epsilon\}$ tends to zero while $p \leq 1$, so $\langle p(C(r)) \rangle_R \to 0$. Since $p(\bar{C}) > 0$, the map $s \mapsto \langle p(C(r)) \rangle_R$ is non-constant; being continuous in s, it takes distinct values at some $s_A \neq s_B$. By (S38), $P(Y{=}1)$ is a strictly increasing function of $E[\Phi]$ at fixed N and Θ_{sys} throughout the non-degenerate range, so the corresponding outputs differ. This establishes the first claim.

For the second claim, observe that the family (S27) is ordered by dilation: increasing s at fixed mean transfers concentration from the centre towards the periphery, and the resulting profiles are ordered in the majorisation sense, with the uniform field minimal. Where p is concave on the effective range, majorisation implies that $\langle p \rangle$ is non-decreasing in s. Where p has a convex region the ordering is not implied by majorisation alone, and we therefore verify monotonicity numerically over the parameter range used; it holds throughout (Supplementary Table S14). Given monotonicity and continuity, the set $\{s : P(Y{=}1) \geq 1/2\}$ is an up-set and s^* is its infimum. □

Remark 7 (Two qualifications)**.** *First, the effect is not specific to binary terminal responses: a spatially resolved smooth model $\Phi_{\text{SR}} = N^{-1} \sum_i h(C_i)$ produces it whenever h is nonlinear, since $\langle h(C_i) \rangle \neq h(\langle C_i \rangle)$. What Proposition 2 excludes is the* mean-only class *(S33), which is nonetheless the class in routine pharmacodynamic use. Second, the magnitude depends on the field family; we report s^* rather than a uniform-versus-point-mass contrast precisely so that the result is not carried by an unrealisable construction (Supplementary Materials S1.18).*

Supplementary Table S14. Monotonicity of $E[\Phi]$ in the dispersion radius at fixed spatial mean $\bar{C} = 1.5\,\bar{C}_{\text{crit}}$, used in the proof of Proposition 2. The system output switches between $s/R = 0.40$ and $s/R = 0.45$, where $E[\Phi]$ crosses $\Theta_{\text{sys}} = 0.60$; the sharpness of that switch is the finite-N effect of Proposition 1 acting along the geometry axis.

s/R	0.20	0.30	0.40	0.45	0.60	1.00	∞
$E[\Phi]$	0.2438	0.4032	0.5457	0.6121	0.7762	0.9122	0.9373
$P(Y{=}1)$	0.0000	0.0000	0.0000	1.0000	1.0000	1.0000	1.0000

https://doi.org/10.3390/toxins18090403

Supplementary Materials S3. The Serotype Asymmetry in Cleavage–Effect Correlation

Cleavage of a small measured fraction of SNAP-25 by BoNT/A suffices for complete blockade, and across preparations the measured fraction correlates poorly with the degree of neuroparalysis; for BoNT/E, cleavage and inhibition of exocytosis track one another. This appendix shows that both features follow from the architecture without additional assumptions, and states carefully what that does and does not establish.

S3.1 Why a collective threshold decorrelates the assay from the outcome

Combining the assay relation (S24) with the system integration (S36) gives, for each mean dose, a pair $\big(\rho\langle f\rangle(\bar{C}),\, P(Y{=}1 \mid \bar{C})\big)$ that an experimenter could in principle plot against one another. Table 4 of the main text evaluates that pair across the transition, at the human reference with $\rho = 0.2277$ and $N = 10{,}000$: the measured cleavage moves by three percentage points while the outcome moves from certain failure to certain block. The two are not uncorrelated over this range—both are monotone in dose—but the outcome transition occupies a band of measured cleavage narrower than any realistic assay can resolve, and the model is deterministic given $\bar{C}$: the apparent decorrelation is produced by the collective threshold and not by measurement noise or biological variability.

The mechanism is the one already established in Proposition 1, but it must be stated as a claim about resolution rather than about correlation. The outcome transition occupies a window of width $\Delta\bar{C}_q \sim \mathcal{K}/\sqrt{N}$ in dose, which at $N = 10{,}000$ is under 1% of $\bar{C}_{\text{crit}}$; the assay, being an average over units, varies across that same window by 0.60% of its own value. An assay whose between-preparation reproducibility is worse than that cannot rank preparations by outcome, whatever the underlying architecture.

It does not follow, and we do not claim, that the correlation between measured cleavage and outcome is small. Over the conditions of Table 4 the two correlate at $r = 0.85$ and $\rho = 0.93$, as they must, since both are monotone in dose and correlation is invariant to rescaling of either axis. Nor does the correlation fall with N: in simulations of preparations with dispersed doses and a log-normally varying pool fraction it is essentially constant from $N = 10^2$ to $N = 10^4$ at fixed pool-fraction dispersion, and is governed almost entirely by that dispersion. What falls as $N^{-1/2}$ is the ratio of the informative band to the assay's dispersion, which is 0.014 at $N = 10^4$ and a coefficient of variation of 0.30 in the pool fraction. An earlier version of this appendix inferred a prediction about the correlation coefficient from the behaviour of the band; that inference is invalid and is withdrawn.

S3.2 Why the two serotypes differ

The degree of apparent decorrelation is controlled by the width of the informative band relative to what the assay resolves. The dominant-negative product enters the model at exactly one place, as β in (S18), and it moves that band—but not through the logistic steepness, and not in the same direction in the two units in which a band can be measured. Both statements are quantitative and both are in Supplementary Table S4; the second is the reason the derivation given in our first revision is superseded.

The two products differ structurally and not only in degree. BoNT/A cleaves at Gln197–Arg198 and leaves SNAP-25_{1-197}, a nine-residue truncation that is still incorporated into the SNARE complex and inhibits release beyond the loss of intact protein [23]—a large β. BoNT/E cleaves at Arg180–Ile181 and leaves SNAP-25_{1-180}, a twenty-six-residue truncation that does not maintain the association [3] and acts close to pure loss of function—$\beta \approx 0$. What that difference does to the informative band is the subject of the next two paragraphs, and it is not what the phrase "a steeper map" would suggest.

What β actually does. Raising β lowers the unit threshold through (S22) and, by (S23), lowers the logistic steepness γ rather than raising it, because γ is matched at the operating point and the operating point moves with it. The absolute slope of the release map, $\gamma/(4\theta)$, does rise—from 2.97 at $\beta = 0$ to 13.2 at $\beta = 15$—so the dominant-negative product does steepen the map in the sense the molecular argument intends; what it does not do is raise γ. Any derivation of the serotype ordering that treats γ as the serotype-specific parameter is therefore inadmissible, and ours did so. Over $\beta \in [0, 15]$, γ runs from 5.93 down to 3.11 (Supplementary Table S4), and $\gamma = 12$ is not attainable at SF $= 2$ under any β: it is the superseded rodent value.

The band, in the two units it can be measured in. Equation (21) gives the excursion as a fraction of the measured cleavage. In that unit the coefficient $B(\langle f \rangle)\, W$ *rises* with β, from 0.6033 at $\beta = 0$ to 0.9362 at $\beta = 6$ and 1.0068 at $\beta = 15$, because the operating point falls faster than the band does. Multiplying by the measured cleavage itself gives the excursion in points of measured cleavage, $\Delta(\rho\langle f \rangle) = \rho\, h(\langle f \rangle)\, W/\sqrt{N}$ with $h(x) = (1-x)\ln[1/(1-x)]$, and in that unit the coefficient *falls*, from 0.3312 at $\beta = 0$ to 0.1372 at $\beta = 6$ and 0.0704 at $\beta = 15$. The pool fraction does not cancel from the absolute form, but it is common to two serotypes measured in one preparation, so the ratio between them is free of it.

The prediction, and the condition on it. At $\beta = 6$—the value (S22) returns at SF $= 2$ from the threshold of Supplementary Materials S1.16 reading (ii), and not a value chosen here—BoNT/A's band is 2.41 times narrower than BoNT/E's in points of measured cleavage, and the ratio lies between 2 and 3 for β between about 4 and 8. That is the reported ordering, and it is the one we advance. It holds if the between-preparation reproducibility of the assay is an absolute error in points of total SNAP-25, which is the regime when two serotypes are run on one platform and the limiting noise is background and transfer; it reverses if the reproducibility is a coefficient of variation on the measured value, since in relative units the ordering is the opposite one. We state both columns rather than the one that favours the account, and we note that the question is empirical: the error model of a densitometric immunoblot is measurable without reference to this model. It is not, in either unit, a prediction about the correlation coefficient, which Supplementary Materials S3.1 withdraws.

We note one confound that this account does not control. The light chain of BoNT/A persists in the cytosol for months and that of BoNT/E for days [1,24], while the published comparisons are made at a common time after exposure. Part of the observed difference in cleavage–effect correlation may therefore reflect sampling two time courses at a shared time point rather than a difference in β. Discriminating the two requires time-resolved cleavage and paralysis measurements in the same preparation, which is a stronger version of the direct test proposed in Supplementary Materials S3.3.

S3.3 What this does and does not establish

We are deliberate about the evidential weight here, because it is easy to overstate.

What it establishes. A pattern routinely described as paradoxical—that cleavage extent and paralysis extent do not track one another—has a quantitative account that requires no auxiliary hypothesis about the toxin: the outcome transition occupies a band of measured cleavage of relative width $0.603/\sqrt{N}$, which for realistic unit counts falls below the between-preparation reproducibility of the assay. The account is about measurement resolution against a sharp transition, and it does not require the architecture to be stochastic. And the serotype asymmetry, which would otherwise need a separate explanation, follows from the same one-parameter family, in the absolute reading of the band and subject to the condition Supplementary Materials S3.2 states.

What it does not establish. It does not establish which error model the published assays obey, and the serotype ordering is conditional on that. Nor does the ordering survive a relative reading of the band, in which it reverses. Beyond that, this is a consistency account and not a fit. We have not extracted correlation coefficients from the published series and compared them against Table 4; doing so would be a direct test of the band width predicted by Equation (21), and not of the correlation coefficient, which Supplementary Materials S3.1 withdraws. The cited observations come from frog neuromuscular junction and rodent spinal cord cultures rather than mammalian NMJ *in vivo*, and safety factors differ markedly across species. Finally, the account does not compete with the dominant-negative mechanism, for which there is direct molecular evidence; it reduces the amount of work that mechanism must do by attributing part of the effect to measurement and part to architecture.

S3.4 Quantitative form and stability of the informative band

What the architecture predicts, and what it does not: Table 4 is evaluated at one parameter set, which invites the objection that the architecture has been shown capable of producing the pattern rather than shown to produce it. The objection is answerable, but answering it requires stating the mechanism more carefully than a qualitative appeal to the collective threshold allows, and we do so here because the careless version is wrong.

Differentiating the assay relation (6) and using $\langle f \rangle = 1 - e^{-\kappa_{\mathrm{eff}}\bar{C}}$ with $\kappa_{\mathrm{eff}} = \lambda(1 - e^{-\eta})$, the excursion of the measured cleavage across the interquantile outcome window, expressed as a fraction of the measured cleavage itself, is Equation (21) of the main text, in which the pool fraction ρ cancels identically. The coefficient is 0.603 at the reference configuration. Its stability is assessed with γ held out, because γ is not a free parameter but a function of quantal content (Supplementary Materials S1.20) whose own excursion is reported separately in Table 2; the serotype dependence acts through β and is in Supplementary Materials S3.2. Across every other factor of the one-factor-at-a-time grid of Supplementary Table S21 the coefficient spans 0.44 to 0.81, a factor of 1.85, while the parameters themselves vary by factors of four; the largest contributions are the fibre layer and the threshold dispersion, not the uptake stage. The width of the informative band is therefore a property of the cleavage-to-release map and of the architecture rather than of the parameterisation, and it does not depend on the quantity Section 6 identifies as the paper's least secure: ρ cancels identically. We note that $B(\langle f \rangle)$ is not a neutral factor—it moves with $\langle f \rangle$ and hence with $\bar{C}_{\mathrm{crit}}$—so the coefficient is not systematically more stable than W itself.

We are explicit about what this does *not* license, because an earlier formulation of this argument overreached. The correlation between measured cleavage and outcome across a dose series is *not* predicted to be small by the architecture alone: in a deterministic model both quantities are monotone in dose, and over the seven conditions of Table 4 they correlate at $r = 0.85$ (Spearman $\rho = 0.93$). Nor is the correlation predicted to fall with N: simulating preparations with dispersed doses and a log-normally varying pool fraction returns essentially the same correlation at $N = 10^2$, 10^3 and 10^4 (for instance 0.37, 0.37 and 0.39 at a pool-fraction coefficient of variation of 0.30), because in the large-N limit the assay's informativeness is set by its own dispersion and not by the sharpness of the transition. The correlation coefficient is therefore not a discriminating observable: this is the third of the withdrawn claims of Supplementary Table S8, and it is withdrawn rather than conditional, since no measurement of the parameters reinstates it. What the architecture predicts is the width of the informative band, Equation (21), and the ordering of that width between the two serotypes.

We remain careful about the evidential weight. Equation (21) establishes that the decorrelation is architectural rather than chosen, but no correlation coefficients have been

extracted from the published series and compared against model predictions, and the cited observations come from frog neuromuscular junction and rodent spinal cord cultures rather than mammalian NMJ in vivo. What the argument establishes is that a pattern often described as paradoxical is a generic consequence of collective thresholding, and that the serotype asymmetry has a one-parameter explanation within the architecture. The direct test is not a comparison of correlation coefficients, which Supplementary Table S8 records as withdrawn: it is a comparison of the width of the informative band, Equation (21), between the two serotypes at equal N and equal assay, for which time-resolved cleavage and paralysis measurements in the same preparation would be required.

Supplementary Materials S4. Extended Dynamical Regimes

Both extensions in this appendix are phenomenological compressions, not mechanistic derivations. Neither the drift rate nor the functional form of uptake impairment has been quantified experimentally, and both are presented as structural illustrations of how the architecture behaves under repeated exposure.

S4.1 Longitudinal threshold drift

Let the mean terminal threshold increase across treatment sessions,

$$\mu_\theta(t) \;=\; \mu_\theta(0) + \delta_0 t \qquad \text{(constant drift)}, \tag{S48}$$

or $\mu_\theta(t+1) = \mu_\theta(t) + \delta_0 \Phi_t$ (response-dependent drift), with σ_θ held fixed. Because block requires the cleaved fraction to reach the Θ_{sys}-quantile of the threshold law, the minimum effective dose at session t solves $1 - e^{-\kappa \bar{C}_t^*} = f_{\text{sys}}^*(t)$, giving

$$\bar{C}_t^* \;=\; -\frac{1}{\kappa} \ln\Big[1 - F_\theta^{-1}\big(\Theta_{\text{sys}};\, \mu_\theta(t),\, \sigma_\theta\big)\Big]. \tag{S49}$$

At $t = 0$ this returns $\bar{C}_0^* = -\ln(1 - 0.5193)/2.0 = 0.3663$, recovering $\bar{C}_{\text{crit}}^{\text{MF}}$ as it must. The attenuation onset t^* is the first session at which $\bar{C}_t^*$ exceeds the safe-dose ceiling $C_{\max}$, which is set at three times $\bar{C}_{\text{crit}}$ as a declared convention: the model contains no calibration to clinical units, so no dose in it has an absolute referent (Supplementary Materials S1.23).

Because F_θ^{-1} is bounded above by 1 under the Beta law (S16), $\bar{C}_t^*$ diverges as $\mu_\theta(t) \to 1$, so escape from the therapeutic window occurs in finite time for any $\delta_0 > 0$. We note that this is a property of the drift assumption rather than a prediction: it states that *if* thresholds drift monotonically upward without bound, escape follows, which is close to a tautology. The substantive and unresolved question is whether such drift occurs at all.

Why the candidate mechanisms do not settle it. Presynaptic sprouting and collateral reinnervation follow BoNT-A poisoning with a characteristic biphasic time course in which transmission is first restored at newly formed sprouts and subsequently returns to the parent terminals, the sprouts then regressing [62]. This is a *within-cycle* phenomenon that largely resolves before the next injection, and it therefore argues against, rather than for, a simple cumulative reading. Recruitment of the BoNT-A-resistant homologue SNAP-23 [63] can support residual exocytosis [64], but SNAP-23 is not normally dominant at the mature mammalian NMJ, and a treatment-driven upward shift in its expression after repeated exposure has not, to our knowledge, been demonstrated *in vivo*. Both are listed as candidate substrates; neither is an established route. Supplementary Figure S3 shows the bounded threshold trajectory and the corresponding minimum effective dose across sessions.

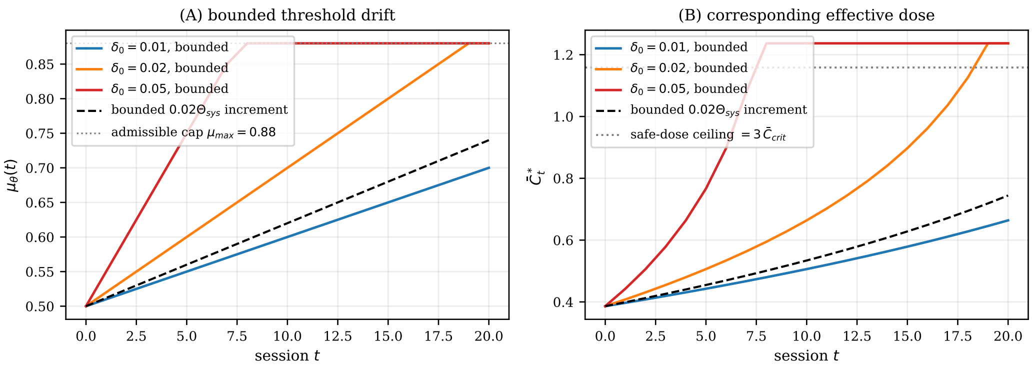

Supplementary Figure S3. Threshold drift. (A) Bounded mean threshold $\mu_\theta(t)$ across sessions for constant drift ($\delta_0 = 0.01, 0.02, 0.05$) and for response-dependent drift ($\delta_0\Phi_t$, $\delta_0 = 0.02$). Trajectories are capped at the admissible maximum $\mu_{\max} = 0.88$, beyond which the Beta threshold law of (S16) cannot accommodate the reference dispersion; the cap is drawn explicitly rather than applied silently. **(B)** The corresponding minimum effective dose $\bar{C}_t^*$, computed from the same bounded trajectory, with the safe-dose ceiling marked. That ceiling is set at three times the critical concentration and is a declared convention: the model contains no calibration to clinical units, so no dose in it has an absolute referent (Supplementary Materials S1.23).

S4.2 *Effective uptake modulation*

Let a fraction $A \in [0, 1)$ of uptake capacity be lost, so that the effective count scale becomes $\lambda_{\text{eff}} = \lambda(1 - A)$. Since the response depends on λ and C only through the product λC in (S6), this is exactly equivalent to a dose reduction, and block requires $\bar{C}(1 - A) \geq \bar{C}_{\text{crit}}$. Hence

$$A^*(\bar{C}) \;=\; 1 - \frac{\bar{C}_{\text{crit}}}{\bar{C}} \tag{S50}$$

in closed form. Expressed in multiples of the critical dose the boundary reduces to the exact identity $A^* = 1 - 1/\text{multiple}$, independent of every parameter of the model: $A^* = 0.167$, 0.333 and 0.600 at 1.2, 1.5 and 2.5 times $\bar{C}_{\text{crit}}$. Above $A^*(C_{\max})$, block is unattainable at any dose within the safe ceiling. That the boundary is parameter-free is worth stating, because it is the sharpest form of the admission below: the ceiling carries no information about the architecture.

What this does and does not show. The existence of a ceiling on attainable effect is *not* a property of the discrete architecture. Any model in which cleavage saturates and the dose is capped at $C_{\max}$ yields the same bound, and a mean-field model with the same $C_{\max}$ yields the same A^*; indeed (S50) contains no reference to N or to σ_θ. What the discrete architecture adds is the *abruptness* of the crossing: because output is binary in Φ, $P(Y{=}1)$ falls from one to zero over an interval in A of width $O(N^{-1/2})$, whereas the mean-only benchmark crosses smoothly. Earlier formulations attributed the ceiling itself to the architecture; that attribution was incorrect and is withdrawn here. Supplementary Figure S4 shows the crossing at the three doses.

Supplementary Materials S5. Computational Verification and Reproducibility

S5.1 *Environment and provenance*

All computations were performed in Python 3.9.6 with NumPy 2.0.2, SciPy 1.13.1 and pandas 2.3.3, in double precision. Random number generation used the PCG64 generator with per-experiment streams derived from an explicit seed (12345 for recovery, 20260826 for verification Monte Carlo, $31337 + S + R + T$ for the power simulation, with S, R and T the subject count, session count and number of target classes, and 20260828 for the subject-

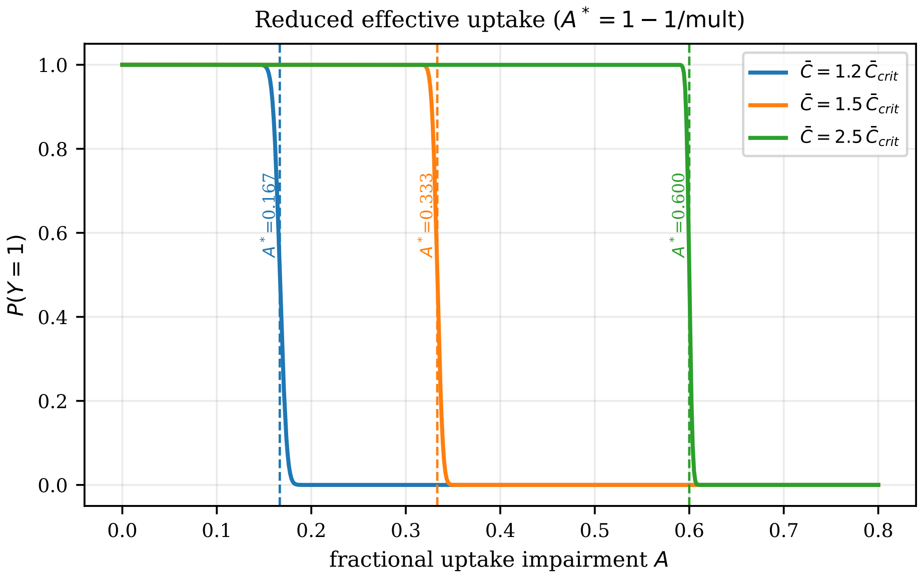

Supplementary Figure S4. Reduced effective uptake. $P(Y{=}1)$ against fractional uptake impairment A at doses of 1.2, 1.5 and 2.5 times $\bar{C}_{\mathrm{crit}}$, with $A^*(\bar{C}) = 1 - \bar{C}_{\mathrm{crit}}/\bar{C}$ marked at 0.167, 0.333 and 0.600. Expressed in multiples of the critical dose the boundary is the exact identity $A^* = 1 - 1/\text{multiple}$, and $P(Y{=}1)$ at A^* is the same at all three doses because the effective dose returns to $\bar{C}_{\mathrm{crit}}$ by construction. The shaded region is unreachable at any of the three doses.

level checks, and `20260901` for the cohort duration check of Supplementary Materials S7.6). Every tabulated quantity in this work is a deterministic function of the parameters except four: the Monte Carlo columns of Tables S11, S15, S16 and S1, which are verifications rather than results; the synthetic recovery of Supplementary Materials S5.6; the design simulation of Supplementary Materials S6.3; and the between-subject convolution check of Supplementary Materials S7.6, in which the finite-size term is integrated exactly and only the between-subject convolution is simulated. Each of the four verification columns falls within its own reported standard error of the analytic value, which is what those tables verify, and an independent run under a different stream reproduces them to that tolerance. The released code comprises the analysis scripts and their shared modules; the manifest is the authoritative list, and the principal entry points are the following. `model_v9.py` implements the model core and `common.py` the shared readout functions on which the others depend. `recovery_v9.py` runs the recovery experiments and `genfigs_v10.py` the figures reproduced here; its predecessor `genfigs_v9.py` runs on different defaults, generates no figure in this paper, and is retained in the archive for provenance only. `graded_v9.py` produces Supplementary Table S19.`fiber_v10.py` supplies the fibre layer of Equation (9), the quantal grounding of Supplementary Materials S1.12 and Supplementary Materials S1.20, and the conditionality machinery of the main text, and `emit_numbers_v10.py` regenerates `numbers_v10.json` from it. `block1_beta.py` produces the β sweep of Supplementary Table S4, `run_power4.py` the design simulation of the main text, `p21_discriminator.py` the architecture comparison of Supplementary Materials S5.10, and `p27_subject_mc.py` the two subject-level Monte Carlo checks of Supplementary Materials S1.7 and of the main text. The latter is worth one remark for anyone repeating it: the individual critical dose must be simulated with each unit carrying its own realisation as well as its own threshold, since replacing the unit state by its probability discards the within-unit component of the Bernoulli variance and narrows the simulated interquartile range by a factor $\sqrt{\mathrm{Var}_\theta(q)/[p(1-p)]}$—which is 0.49 under the binary reading and 0.32 under the quantal one, and is a trap rather than a disagreement. Earlier versions of this work reported Supplementary Table S6(b)

without releasing the code that produced it, and one row of that table ($\bar{m}_0 = 30$) did not reproduce; the corrected value is used throughout and the computation is now covered by the manifest.

Provenance is machine-checkable rather than asserted. Each run records, in the checkpoint it writes, the seed, the protocol, the parameter bounds, the library versions and the SHA-256 digest of every analysis script; a manifest of digests covers every released file, including the shared modules and any script not named above; and a test suite accompanies the code. Resuming an interrupted run verifies that metadata before reusing any stored result, so results produced under different protocols cannot be combined—a failure mode that is easy to introduce, silent when it occurs, and capable of mixing protocols within a single reported table.

S5.2 Reference implementation

The listing below is the numerical core. It exploits the observation of Supplementary Materials S1.5 that the threshold weights are independent of concentration.

```
def theta_cdf(x, mean, sd):
    """Beta CDF with given mean and sd; support [0,1] by construction."""
    nu = mean * (1 - mean) / sd**2 - 1.0
    a, b = mean * nu, (1 - mean) * nu
    return betainc(a, b, np.clip(x, 0.0, 1.0))

def p_su(C, kappa, lam, mu, sd):
    """p^SU(C) = sum_k Pois(k; lam*C) * F_theta(1 - exp(-eta*k)).
    The weights w_k do not depend on C: compute once, then contract."""
    eta = kappa / lam
    m   = lam * np.atleast_1d(C)
    kmax = int(pdtrik(1 - 1e-12, m.max())) + 8
    ks = np.arange(kmax + 1.0)
    w  = theta_cdf(1.0 - np.exp(-eta * ks), mu, sd)      # C-independent
    logpmf = ks * np.log(m[:, None]) - m[:, None] - gammaln(ks + 1.0)
    return np.exp(logpmf) @ w

def py_from_p(p, N, Theta_sys):
    """P(Bin(N,p) >= ceil(Theta*N)) exactly, via regularised beta."""
    k = int(np.ceil(Theta_sys * N)) - 1
    return betainc(k + 1.0, N - k, p)
```

Listing 1: Terminal silencing probability and system integration.

S5.3 Numerical architecture, parameter structure and continuous benchmarks

Relation to Stochastic Reaction Modeling

The discrete formulation is conceptually related to stochastic descriptions of biochemical reaction networks [70], but the resemblance is limited in the way Section 3.10 makes precise: such frameworks derive their content from the randomness of individual reaction events, whereas the present model derives its content from the finiteness of the unit population and the heterogeneity of terminal thresholds.

Computational Environment

Simulations were implemented in Python 3.9.6 using NumPy 2.0.2 for array operations, with pseudo-random numbers from the PCG64 generator [71]; SciPy 1.13.1 was used for analytical verification of the tabulated results (see Supplementary Materials S5.4) and is not a dependency of the simulation engine itself (see Supplementary Files S1–S3 for the complete annotated source code). Seeds are fixed per experiment rather than globally and are listed in Supplementary Materials S5.1; all results are exactly reproducible given identical library versions. No GPU or multi-process code was used.

Numerical Architecture

The unit silencing probability is the exact Poisson mixture of Supplementary Materials S1, evaluated as a single matrix–vector product because the threshold weights do not depend on concentration; the system response under a uniform field is the exact binomial tail, obtained from the regularised incomplete beta function. Under a spatially heterogeneous field the unit states are independent but not identically distributed, so the exact binomial tail does not apply and the first three cumulants of the Poisson–binomial are used with a continuity-corrected Edgeworth expansion, stated as such wherever those numbers appear.

Each verification run instantiated $N = 10{,}000$ units, drew thresholds $\theta_i \sim \text{Beta}(21.72, 21.72)$ from (S16), drew uptake counts $K_i \sim \text{Poisson}(\lambda C_i)$, evaluated $S_i = \mathbb{I}(f_i \geq \theta_i)$, and recorded Φ and Y, in a vectorised implementation with batch size 2,000 (this appendix).

Parameter Structure

Parameter values and biologically motivated ranges are summarized in Supplementary Table S7. The reference condition used $\kappa = 2.0$, $\lambda = 35$, $\mu_\theta = 0.50$, $\Theta_{\text{sys}} = 0.60$, and the threshold coefficient of variation $\sigma_\theta/\mu_\theta = 0.15$, which is carried over from the earlier parameterisation as a declared assumption rather than a measurement (Section 4.11). Sensitivity was assessed by varying each parameter independently in the ranges in Supplementary Table S7 while holding all the others at reference values. For uptake-robustness analysis, the same one-factor-at-a-time grid was evaluated in parallel under the reduced mean-field uptake model and under an uptake-refined stochastic-uptake formulation, so that shifts in $\bar{C}_{\text{crit}}$, $P(Y{=}1)$, $\text{Var}(Y)$, and geometry-dependent contrasts could be attributed specifically to the mean-field replacement of the uptake count rather than to changes in the underlying parameter grid.

Primary and reference models. Throughout this work the stochastic-uptake (SU) formulation, in which $K_i \sim \text{Poisson}(\lambda C_i)$ is retained at each unit (Supplementary Materials S1), is the primary model. The mean-field (MF) reduction, which replaces the count by its expectation before applying the threshold, is retained as the analytically tractable reference: its closed forms admit direct derivation of both propositions and of the dynamical extensions. The two differ by 5.4% in $\bar{C}_{\text{crit}}$ at the reference count scale, and by more only in the degenerate corner of Section 4.6. All numerical values are reported in pairs $(\bar{C}^{\text{MF}}_{\text{crit}}, \bar{C}^{\text{SU}}_{\text{crit}})$; standalone MF values should not be read as calibrated predictions.

The SU and MF formulations were additionally compared side by side with respect to $P(Y{=}1)$, the transition width and the location of $\bar{C}_{\text{crit}}$, over $\lambda \in [1, 500]$ with the composite $\kappa = \lambda\eta$ held fixed so that the mean-field prediction is unchanged by construction (Supplementary Table S18, Section 4.6).

Continuous Mean-Field Benchmark

The continuous benchmark was the Hill-type model:

$$E_{\text{MF}} = \frac{E_{\max}\,\bar{C}^{\,n}}{EC_{50}^{n} + \bar{C}^{\,n}}, \tag{S51}$$

calibrated by least-squares fitting to the discrete model mean curve independently for each spatial field geometry.

The Hill formulation is a canonical representative of the mean-field class [13], so the comparison is structural rather than pharmacological, and its fitted parameters carry no mechanistic interpretation. Structural non-equivalence is assessed by comparing $E_{\text{MF}}(\bar{C})$ against $P(Y = 1)$ across the full concentration range and across field geometries.

Spatially Resolved Continuous Benchmark

To assess whether geometry dependence is specific to the discrete threshold architecture or is already reproduced by spatially resolved smooth models, we introduced a Class II benchmark in which the Hill function of Supplementary Equation (S51) was applied unit by unit:

$$\Phi_{\mathrm{SR}}(\{C_i\}) = \frac{1}{N}\sum_{i=1}^{N} \frac{E_{\max}\, C_i^n}{EC_{50}^n + C_i^n}, \tag{S52}$$

with Hill parameters calibrated to the uniform-field discrete curve (identical to the Class I benchmark). The system output was then $Y_{\mathrm{SR}} = \mathbb{I}(\Phi_{\mathrm{SR}} \geq \Theta_{\mathrm{sys}})$. The benchmark was evaluated across the diffusion field of Equation (10) at dispersion radii spanning $s/R = 0.2$ to the uniform limit, which separates geometry effects attributable to spatial resolution alone (Class II) from those attributable to the discrete threshold response.

S5.4 *Numerical accuracy*

Three checks bound the numerical error.

(i) The Poisson sum (S6) is truncated where the residual tail falls below 10^{-12}, with eight additional terms retained; agreement with an independent scalar implementation is 1.4×10^{-14} over randomised parameter draws.

(ii) The radial quadrature of (S27) uses 80-node Gauss–Legendre in $u = r^2/R^2$; agreement with a 400-node computation is below 10^{-14}.

(iii) Under a uniform field the system integration uses the regularised incomplete beta function, so $P(Y{=}1)$ is exact to machine precision. Under a heterogeneous field the unit states are independent but not identically distributed, the binomial tail does not apply, and we use the first three cumulants of the Poisson–binomial with a continuity-corrected Edgeworth expansion; the reference implementation records this in its output metadata. Every geometry quantity in this work—Supplementary Table S14, the critical dispersion radius, and the fractionation results—rests on that approximation and not on an exact tail. We therefore bound it. The Poisson–binomial tail over the exact vector of $N = 10^4$ unit probabilities is computable by discrete Fourier transform, and was validated against the exact binomial tail under a uniform field to 10^{-13}. Compared against it at $\bar{C} = 1.5\,\bar{C}_{\mathrm{crit}}$ and $s/R = 0.40, 0.44, 0.45$, the continuity-corrected Edgeworth expansion departs by at most 5.4×10^{-6} in $P(Y{=}1)$, and the resulting displacement of s^* is 9×10^{-7} in s/R—one part in 4.8×10^5. The Gauss–Legendre quadrature contributes a further 5.7×10^{-9}, so the 80 nodes of Supplementary Materials S1.18 are effectively exact. The approximation is thus controlled at a level several orders below any quantity reported from it.

S5.5 *Verification of the critical regime*

Supplementary Table S15 verifies the analytical dose–response against direct Monte Carlo simulation of the binomial population, and Supplementary Table S16 documents Monte Carlo convergence at $\bar{C}_{\mathrm{crit}}$, where estimator variance is maximal.

S5.6 *Synthetic recovery: design, criteria, and scope*

Because the framework is not fitted to empirical data, we assessed how well target quantities can be recovered from aggregate outputs. Four design choices determine what the results mean, and each is stated rather than left implicit.

Observation model. Observables are binomial responder counts at $R = 625$ replicates per dose point. The obvious alternative—perturbing exact probabilities with additive Gaussian noise and clipping the result to $[0, 1]$—biases the observation near 0, near 1 and

Supplementary Table S15. Analytical and Monte Carlo agreement across the three regimes at $N = 10{,}000$ (20,000 replicates). $z_{\mathrm{sys}} = \sqrt{N}\,(p - \Theta_{\mathrm{sys}})/\sqrt{p(1-p)}$ is the standardised distance from the collective threshold. The transition is complete within ±3% of $\bar{C}_{\mathrm{crit}}$, which is the finite-N sharpness of Proposition 1 expressed in dose units. Computed at the rodent parameterisation, retained here because these are verifications of Proposition 1 rather than parametric results; the proposition and its proof are unchanged at the human reference.

**Regime**	$\bar{C}$	$p(\bar{C})$	$P(Y{=}1)$ analytic	$P(Y{=}1)$ MC	$\mathrm{Var}(Y)$	z_{sys}
Sub-critical	0.6750	0.569975	0.0000	0.0000	0.0000	−6.06
Critical	0.6959	0.600000	0.5043	0.5019	0.2500	0.00
Supra-critical	0.7168	0.628948	1.0000	1.0000	0.0000	+5.99

Supplementary Table S16. Monte Carlo convergence at $\bar{C} = \bar{C}_{\mathrm{crit}}$, the point of maximal estimator variance. The analytical value is 0.5043. Standard errors follow the expected $n^{-1/2}$ decay and every 95% interval covers the analytical value. Computed at the rodent parameterisation, retained here because these are verifications of Proposition 1 rather than parametric results; the proposition and its proof are unchanged at the human reference.

replicates n	$\hat{P}(Y{=}1)$	standard error	95% interval
1,000	0.50200	0.01581	±0.03099
5,000	0.49180	0.00707	±0.01386
20,000	0.50975	0.00353	±0.00693
100,000	0.50254	0.00158	±0.00310

near the variance maximum, and corresponds to no experiment. Binomial sampling is both unbiased and the actual sampling law of a responder-status study.

Observable blocks. The dose–response curve, the multi-N ladder, the geometry grid and the uptake-modulation boundary are separate blocks. An output-variance block is deliberately *absent*: since Y is Bernoulli, $\mathrm{Var}(Y) = P(Y{=}1)[1 - P(Y{=}1)]$ exactly, so a variance observable carries no information the mean curve does not already carry. Treating the two as independent channels, and adding independent noise to each, manufactures information that no measurement supplies and inflates apparent recovery.

Geometry at fixed physical doses. Candidates are evaluated at the same physical concentrations used to generate the data. Rescaling the evaluation doses by each candidate's own $\bar{C}_{\mathrm{crit}}$ compares predictions and data at different physical conditions, and can produce discrepancies as large as unity in probability.

Optimiser and failure handling. The system threshold enters through $\lceil \Theta_{\mathrm{sys}} N \rceil$, so the objective is piecewise constant in Θ_{sys} and gradient-based optimisers report convergence while leaving that coordinate at its starting value. Powell's method is used instead. Starts that fail remain failures and are counted; they are never replaced by fallback parameter values. Per-experiment random streams, and checkpoint metadata recording seed, protocol, bounds, library versions and SHA-256 digests of the analysis scripts, make resumed runs deterministic and make it impossible to combine results generated under different protocols.

S5.7 *What the recovery study settles, and what it does not*

Three distinct questions are easily conflated—structural identifiability, practical identifiability, and recovery performance—and conflating them is the commonest way for a recovery study to overstate what it has shown. The logic is asymmetric: good recovery is evidence for practical identifiability under the design used and establishes nothing structural, while poor recovery is ambiguous in isolation, since it may reflect structure, noise, design or numerical failure. That asymmetry is why substituting fallback values for

Supplementary Table S17. Recovery performance, PR1–PR7 with the matched control PR5b, at the human reference. Each experiment uses 12 pseudo-datasets × 8 optimiser starts; all 96 starts converged in every experiment and no dataset failed. "Truth" is the generating model and "fit" the model fitted to the pseudo-data (MF = mean-field reduction, SU = stochastic uptake). **Pseudo-observations are generated under the binary terminal response**, since the recovery machinery carries no γ; the results therefore speak to the design and not to the graded operating point reported elsewhere. Errors are median relative errors of the target across pseudo-datasets. The graded columns repeat the identical experiment under the quantal terminal response with γ known exactly; only PR5 and PR5b were rerun, and the remaining rows are left blank rather than carried over. The recovery class in the last column refers to the graded readout, which is the operating point the paper uses. Class names describe recovery performance under this design and noise level; see Supplementary Materials S5.7 for why they are not statements of identifiability.

					Binary readout		Graded readout		
**Exp.**	**Truth / fit**	**Observable blocks**	**Target**	**True value**	**Med. err. (%)**	**IQR (%)**	**Med. err. (%)**	**IQR (%)**	**Recovery**
PR1	MF / MF	dose	κ	2.0000	68.10	32.28	—	—	Poorly recovered
PR2	SU / SU	dose	$\bar{C}_{\mathrm{crit}}$	0.386192	0.01	0.02	—	—	Well recovered (composite)
PR3	SU / MF	dose	$\bar{C}_{\mathrm{crit}}$	0.386192	0.02	0.01	—	—	Well recovered (composite)
PR4	SU / SU	dose	$\Delta\bar{C}_{0.75}$	0.002279	1.74	4.17	—	—	Well recovered (composite)
PR5	SU / SU	**multi-N ladder**	σ_θ	0.0750	**1.87**	**5.43**	**9.43**	**37.36**	**Poorly recovered (graded)**
PR5b	SU / SU	dose (single N)	σ_θ	0.0750	**4.25**	**10.18**	**19.53**	**55.22**	Poorly recovered (graded)
PR6	SU / SU	dose + geometry	s^*	0.345308	2.84	3.73	—	—	Well recovered (composite)
PR7	SU / SU	dose + uptake	A^*	0.742551	0.00	0.00	—	—	Well recovered (composite)

failed optimiser starts destroys the inference entirely: a total failure is then recorded as a perfect recovery. We therefore name the classes after recovery performance, and settle the structural question separately where it can be settled.

The structural question for κ: what we can and cannot say. Optimising the noiseless mean-field curve over $(\kappa, \mu_\theta, \sigma_\theta)$ from widely separated starting points returns the generating parameters every time, with residual root-mean-square error of order 10^{-15}, and no alternative parameter vector was found within the Beta family that reproduces the curve. We state precisely what this is and is not. It is strong numerical evidence *against* a structural degeneracy, obtained under the same logic that governs the rest of this section: a search that fails to find an alternative constrains, but does not prove, that none exists. It is not a proof of structural identifiability, which would require an algebraic argument on the forward map, and we do not offer it as one. An earlier version of this appendix described κ as structurally identifiable on the strength of this search; that was the same inference this section warns against, in the opposite direction, and it is withdrawn.

What defeats estimation is the flatness of the surface. Fixing κ and re-optimising $(\mu_\theta, \sigma_\theta)$, the residual stays below 2×10^{-3}—an order of magnitude under the observational noise of 625 binomial replicates—for every κ from 0.80 to 2.92, and below the noise itself out to $\kappa = 4.54$, a factor of 5.7. The 68% median error of PR1 is the quantitative consequence of that ridge, not a numerical artefact: all 96 starts converged.

The design comparison, which is the strongest claim available. PR5 and PR5b share generating model, observation model, optimiser, protocol and seed, and differ only in whether curves from five terminal counts or one are supplied. Under the binary readout, median error on σ_θ falls from 4.25% to 1.87% and the interquartile range from 10.18% to 5.43%. Because the arms are matched in every other respect, that twofold separation is attributable to the design. It does not survive the operating point of the main text: under the

quantal terminal response the same two experiments give 9.43% and 19.53%, both classified as poorly recovered, and the ratio on the median falls to 0.96× once γ carries the uncertainty its quantal derivation permits. What the comparison establishes, and all it establishes, is the direction—the N-dependence predicted by Proposition 1 carries information about threshold dispersion that a mapping containing no N cannot supply—and not the factor of two.

Two results that cut against us. $\bar{C}_{\mathrm{crit}}$ is recovered to 0.02% even under model misspecification (PR3), which is expected—it is the median effective concentration, read almost directly off the curve—and is not evidence that the architecture is identified. And PR2, PR4, PR6 and PR7 recover *functionals* of the response rather than parameters; recovering a functional constrains the underlying parameters only weakly. We add what the recovery study does not test, since its scope is easily overstated. The generating and fitted models coincide, so the results speak to estimation under correct specification only. Robustness to a misspecified threshold family, to within-unit correlation, to error in N itself, and to an imperfectly defined endpoint has not been assessed, and each of these would degrade recovery in ways the present design cannot bound.

S5.8 Three questions, and the uptake–cleavage composite

Three questions, not one. *Structural identifiability* (whether distinct parameter vectors give the identical noiseless response) is algebraic; *practical identifiability* is relative to a stated noise level and design; *recovery performance*—what Supplementary Table S17 reports—is the median error of a point estimate under one design, one noise model and one optimiser. Good recovery is evidence for the second and establishes nothing about the first, and poor recovery is ambiguous in isolation (Supplementary Materials S5.7). We therefore name the classes after recovery performance and settle the structural question separately where we can.

The uptake–cleavage composite. For κ the structural question has a clean answer, and it is not the one a recovery experiment on its own would suggest. Optimising the noiseless mean-field curve from widely separated starting points returns the generating parameters every time, to a residual of order 10^{-15}: no alternative $(\kappa, \mu_\theta, \sigma_\theta)$ reproduces the curve within the family examined. We report this as numerical evidence against a structural degeneracy rather than as proof of structural identifiability, which would require an algebraic argument on the forward map. What defeats estimation is the flatness of the surface rather than a degeneracy. At the observational noise achievable with 625 binomial replicates (standard deviation $\simeq 0.020$ near the transition), every κ between 0.80 and 4.54—a factor of 5.7—remains compatible with the data once μ_θ and σ_θ are re-optimised. The 68% median error of PR1 is the quantitative consequence of that flat ridge, and not a failure of the optimiser: all 96 starts converged.

S5.9 Sensitivity tables

How the ranges of Table 2 were chosen. Excursions there are computed as $(W_{\max} - W_{\min})/W_{\min}$ over a grid rather than at the endpoints, three of the factors being non-monotone. Five rows need their range explained, and two of the five differ from the sweep recorded in Supplementary Table S7 for a reason worth stating once: that table records the widest range over which each parameter was ever assessed, whereas this column records the range over which the excursion of W was computed. The uptake count scale is swept over $\lambda \in [1, 500]$ in Supplementary Table S18 to locate the degenerate corner, but the excursion is taken over 10–100, which is the interval on which the expected count at the operating point stays above the threshold of Supplementary Materials S1.23 and the model is therefore defined; below it the apparent excursion measures the degeneracy and not

the sensitivity. The number of endplates per fibre is tabulated to $k = 5$ in Supplementary Table S3 and the excursion is taken over the same range, the variation being non-monotone with its extremes at $k = 1$ and $k = 2$. The response steepness is not free: it follows quantal content through (19), and the range assessed is the one implied by $\bar{m}_0 = 20$–30 quanta per impulse [69]; over the wider illustrative range $\gamma \in [3, 12]$ the excursion would be 121%, which overstates what the derivation permits. The threshold dispersion is assessed to 0.175 although Section 4.11 excludes that value on independent grounds, so 35.3% is a conservative upper bound. The excursion row is computed over 0.075–0.175; that is, from the human reference upward, the wider 0.05–0.175 of Supplementary Table S7 is what the joint sampling draws from, and Supplementary Figure S1(C) plots a wider sweep still, 0.04–0.20, in the binary limit. The three are stated separately rather than merged because they answer different questions. That the excursion range is one-sided about the reference, unlike those of μ_θ and $\Theta_{\rm sys}$, is conservative in the direction that matters: extending it below 0.075 would enlarge the excursion of the factor that already leads the column, not reduce it. And β acts through μ_θ and γ, which carry their own rows, its excursion being quoted at fixed expected count (Supplementary Materials S1.14). The uptake–cleavage composite appears as $\eta = \kappa/\lambda$ and not as κ, because W is exactly invariant to a joint rescaling of (κ, λ)—that is, to a pure dose gain, as Corollary 3.7 requires—and moves only through the per-event yield relative to the threshold dispersion. The joint sampling uses 20,000 draws, the first-order indices summing to 0.926 with the residual being interaction; the one-factor excursions are misleading as a ranking, not wrong as excursions, and they understate the joint spread by 12%. Entries marked — were not sampled jointly: β is a modelling choice fixed at 0 at the reference rather than a parametric uncertainty, and ρ enters no dimensionless quantity. The $\Theta_{\rm sys}$ range is centred on an interior minimum of W; over $[0.30, 0.80]$ the excursion is 23.92%.

Supplementary Table S18. Sensitivity of the mean-field reduction to the uptake count scale λ, at fixed composite $\kappa = \lambda\eta = 2.0$, at the human reference. **Evaluated in the binary limit**: the sweep concerns the uptake gate and the degeneracy at low counts, which is a property of the entry stage and not of the terminal response, and the binary reading isolates it. At the reference $\lambda = 35$ the graded operating point is $\bar{C}_{\rm crit} = 0.4096$ with $E[K] = 14.34$; the binary values tabulated here are 0.3862 and 13.52. Because the mean-field prediction depends only on κ, $\bar{C}^{\rm MF}_{\rm crit} = 0.3663$ throughout and the entire discrepancy is a property of λ. The last column is diagnostic: when a single productive entry is by itself almost sufficient to silence a unit, the threshold θ_i ceases to do any work and the model collapses onto the Poisson gate (S5). That corner is more severe at the human reference than at the rodent one—+150% against +44% at $\lambda = 1$, with a single entry sufficing with probability one—because the halved threshold is easier to clear on one event.

λ	η	$E[K]$ at $\bar{C}_{\rm crit}$	CV(K)	$\bar{C}^{\rm SU}_{\rm crit}$	shift	P(one entry suffices)
1	2.0000	0.9	1.04	0.9163	+150.2%	1.00
2	1.0000	1.0	1.02	0.4764	+30.1%	0.961
5	0.4000	2.3	0.66	0.4567	+24.7%	1.1×10^{-2}
10	0.2000	4.2	0.49	0.4209	+14.9%	1.1×10^{-6}
20	0.1000	8.0	0.35	0.3977	+8.6%	6.5×10^{-12}
**35**	**0.0571**	**13.5**	**0.27**	**0.3862**	+**5.4**%	1.3×10^{-16}
50	0.0400	19.1	0.23	0.3811	+4.0%	9.1×10^{-20}
100	0.0200	37.4	0.16	0.3744	+2.2%	4.9×10^{-26}
500	0.0040	184.0	0.07	0.3681	+0.5%	5.2×10^{-41}

Supplementary Table S21 summarises the one-factor-at-a-time grid over which every result above was checked.

Supplementary Table S19. Graded terminal response (S28): transition width recomputed at $N =$ 10,000, human reference. The binary limit gives $\Delta\bar{C}_{0.75} = 0.002279$. Graded responses widen the transition monotonically, so the binary idealisation is the narrowest transition compatible with the architecture and $\mathcal{K}$ is a lower bound. The requirement is more demanding here than at the rodent parameterisation: agreement within 10% requires $\gamma \gtrsim 20$, and the quantally derived reference $\gamma = 5.93$ departs by 66%.

γ	$\Delta\bar{C}_{0.75}$	deviation	γ	$\Delta\bar{C}_{0.75}$	deviation
2	0.011346	+398%	10	0.002896	+27%
3	0.006846	+200%	12	0.002726	+20%
4	0.005135	+125%	16	0.002544	+12%
**5.93**	**0.003778**	**+66%**	20	0.002454	+7.7%
6	0.003750	+65%	30	0.002359	+3.5%
8	0.003186	+40%	50	0.002308	+1.3%
			∞	0.002279	—

Supplementary Table S20. Critical dispersion radius under fractionated deposition with the ring radius optimised, against the fixed convention $r_0 = 0.5R$ of Supplementary Table S24. Each cell gives the optimal r_0/R and the resulting s^*/R; $n = 1$ places the single site at the centre and has no ring. The optimum lies between $0.60R$ and $0.72R$ throughout and moves inward as the dose rises, the sole exception being $n = 3$ at $1.1\,\bar{C}_{crit}$, where block is only just attainable. The gain over $r_0 = 0.5R$ grows with the number of sites and falls with dose, so it is largest in exactly the regime the fractionation argument concerns: many sites at a contained dose. The optimum is broad—at $n = 9$ and $2.0\,\bar{C}_{crit}$, s^*/R varies by 1.4% over an interval in r_0 of width $0.12R$—so the tabulated r_0 should be read as a range near 0.6–0.7 and not to three decimals, while the value of s^*/R is robust. Binary limit, human reference, $N = 10^4$.

$\bar{C}/\bar{C}_{crit}$	$n = 1$	$n = 3$	$n = 5$	$n = 7$	$n = 9$
1.1	0.6328	0.881 / 0.4486	0.721 / 0.2566	0.652 / 0.1961	0.648 / 0.1799
1.5	0.4407	0.696 / 0.3094	0.679 / 0.1896	0.613 / 0.1508	0.648 / 0.1386
2.0	0.3792	0.656 / 0.2664	0.674 / 0.1664	0.604 / 0.1339	0.702 / 0.1218
2.5	0.3492	0.639 / 0.2451	0.672 / 0.1544	0.601 / 0.1251	0.701 / 0.1131
3.0	0.3305	0.632 / 0.2317	0.671 / 0.1468	0.599 / 0.1194	0.701 / 0.1076
4.5	0.2997	0.621 / 0.2094	0.667 / 0.1339	0.597 / 0.1096	0.697 / 0.0982
Reduction in s^*/R relative to $r_0 = 0.5R$					
1.1	—	19.1%	51.5%	61.8%	64.3%
1.5	—	8.7%	26.9%	33.4%	35.4%
3.0	—	3.9%	15.5%	19.9%	24.0%
4.5	—	3.2%	14.0%	18.0%	22.2%

Supplementary Table S21. One-factor-at-a-time parameter grid. Each row varies a single parameter from the reference configuration of Supplementary Table S7, and the sharpness law was verified to hold at every grid point. Rows are ordered by the excursion of the measurable scale-free width W, which is given in Table 2 of the main text. The β row is evaluated at fixed $E[K]$ (Supplementary Materials S1.14); $\eta = \kappa/\lambda$ replaces κ because W is exactly invariant to a joint rescaling of the two. All $\bar{C}_{crit}$ ranges are the graded reading.

**Factor**	**Values**	$\bar{C}_{crit}$ range
γ	5.69, 5.93, 6.86 ($\bar{m}_0 = 20, 25, 30$)	0.41–0.41
σ_θ	0.075, 0.125, 0.175	0.40–0.42
λ	10, 35, 100	0.39–0.45
η	0.029, 0.057, 0.114	0.21–0.82
β	0, 6, 15	0.04–0.41
k	1, 2, 3	0.41–0.61
Θ_{sys}	0.50, 0.60, 0.70	0.36–0.47
μ_θ	0.40, 0.50, 0.60	0.30–0.54
N	20–50,000	unchanged

S5.10 The discriminator computation

Supplementary Table S22 gives the quantities behind Table 3 of the main text. All three families are evaluated on the same per-unit response—the human reference under the quantal terminal reading—so that the only difference between the first two is the rule that converts silenced units into an outcome. The fixed reserve is $c = 21$, chosen so that $N - c + 1 = \lceil \Theta_{\text{sys}} N \rceil = 30$ at $N = 50$; any other calibration shifts the second column vertically without changing its slope.

Quantiles are exact, not simulated. For a count threshold k on N units, $P(\text{Bin}(N, p) \geq k) = I_p(k, N - k + 1)$, so the per-unit probability at quantile q is the inverse regularised incomplete beta function $p_q = I^{-1}(k, N - k + 1; q)$ and the corresponding dose is $p^{-1}(p_q)$; for the multi-hit family, $P(\text{Poisson}(\lambda) \geq n) = P(\text{Gamma}(n, 1) \leq \lambda)$ gives λ_q in closed form. The reference row reproduces $\bar{C}_{\text{crit}} = 0.40960070646423\,93$ and $W(N = 10^4) = 0.9224672756988732$ to full double precision, and the computation aborts if it does not.

Supplementary Table S22. The three architectures across target size. Columns 2–4 concern the fractional threshold alone: the median effective dose in model units, its relative displacement from the mean-field $\bar{C}_{\text{crit}} = 0.409601$, and that displacement multiplied by N. The product is constant to within 4.1% across a fiftyfold range, which is the signature of an $O(N^{-1})$ finite-size correction rather than a trend; the total excursion of D_{50} over the range is 3.62%. Columns 5–6 give the median dose of the two alternatives normalised at $N = 50$, and columns 7–9 the interquartile width in absolute dose units. The fixed-count family is undefined at the extremes: at $N \leq 20$ the requirement $k = N - c + 1 \leq 0$ is met at zero dose, and at $N \gtrsim 940$ at the reference parameters it demands a silenced fraction above the attainable ceiling $\phi_{\max} = 0.9787$ and cannot be met at any dose. That threshold moves between roughly 330 and 5900 across the declared ranges of γ and μ_θ, so the row is bounded by the ceiling in every case but not at the tabulated size; the tabulated range is the one at the reference.

	**Fractional threshold**			**D_{50}, normalised**		**Interquartile width, absolute**		
N	D_{50}	rel. displ.	$\times N$	fixed count	multi-hit	fractional	fixed count	multi-hit
20	0.394981	-3.569×10^{-2}	-0.7139	—	0.3960	0.08022	—	5.978
50	0.403605	-1.464×10^{-2}	-0.7319	1.0000	1.0000	0.05232	0.05232	9.504
75	—	—	—	1.2261	1.5033	0.04302	0.05732	11.654
100	0.406577	-7.381×10^{-3}	-0.7381	1.3899	2.0067	0.03739	0.06260	13.465
200	0.408082	-3.707×10^{-3}	-0.7413	1.8434	4.0201	0.02658	0.08645	19.060
500	0.408992	-1.486×10^{-3}	-0.7432	2.8671	10.0603	0.01686	0.21519	30.153
1000	0.409296	-7.439×10^{-4}	-0.7439	—	20.1274	0.01194	—	42.651
Log–log slope of the absolute width, $d \ln(\text{IQR}) / d \ln N$						-0.4887	$+0.6149$	$+0.5020$

The last row is the second discriminator of the main text, and it is the one that needs no normalisation: the fractional width contracts at very nearly the $-1/2$ of Proposition 1, while both alternatives expand. The sign, not the magnitude, carries the argument.

Detectability follows from the Fisher information for $\ln D_{50}$ at a dose placed exactly at D_{50}, which is the most informative single placement: $\text{SE}(\ln D_{50}) = 2/(n_{\text{eff}}\sqrt{n_{\text{arm}}})$. Comparing two independent targets at $\alpha = 0.05$ two-sided with power 0.80 requires a log ratio of at least $2.802\sqrt{2}$ standard errors. Supplementary Table S23 evaluates this at the two responder curves for which published dose-ranging data exist. Because the dose is placed at the median, these are upper bounds on the precision obtainable from a given number of subjects, not the yield of a realistic multi-dose design.

S5.11 Supporting figures

S5.12 Scope of the results

Every quantity reported in this supplement is internal to the model. No parameter has been estimated from empirical data, and no numerical value should be read as a calibrated pharmacological prediction. The results intended to carry weight are structural: the $N^{-1/2}$ sharpness law with its prefactor (Proposition 1), its non-transportability corollary

Supplementary Table S23. Smallest detectable displacement of the median effective dose between two targets, at the effective Hill exponents recoverable from published glabellar dose-ranging studies. The multi-hit prediction of 20–50× is detectable with a margin of about an order of magnitude; the fixed-count prediction of 2.87× is marginal at forty subjects per arm and out of reach at twenty. The steeper of the two exponents is the multi-point refit of the abobotulinumtoxinA study; the flatter is retained as an illustration of how quickly precision degrades with a shallow curve and is not offered as a measurement, since the study it derives from does not sample its own transition.

**Responder curve**	n_{eff}	n per arm	**Detectable ratio**
Steepest available	1.486	20	3.30×
Steepest available	1.486	40	2.32×
Illustrative flat curve	0.596	20	19.56×
Illustrative flat curve	0.596	40	8.19×

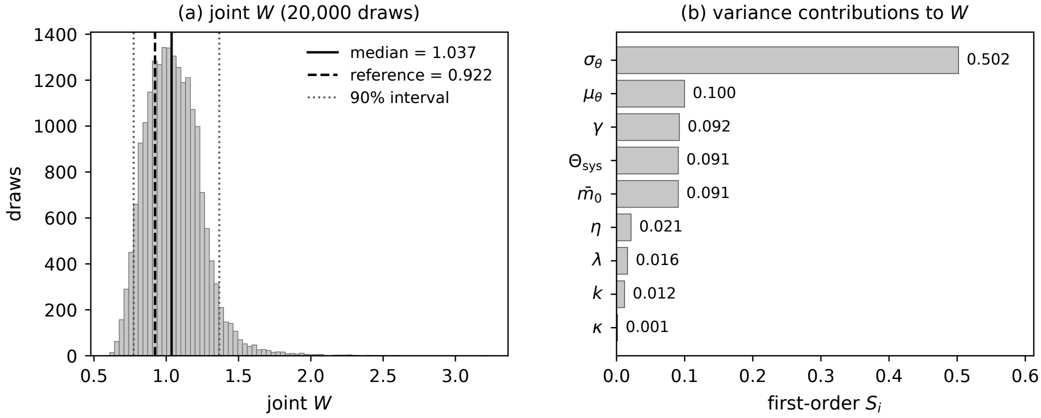

Supplementary Figure S5. Joint parameter uncertainty in the scale-free width W. **Left:** The distribution of W under simultaneous sampling of the declared ranges of Supplementary Table S7, with the reference value and the central 90% interval marked. The reference sits near the lower quartile rather than at the centre, so $W = 0.9225$ should be read as one configuration and not as a central estimate. **Right:** First-order contributions to the variance of W. Threshold dispersion carries more than half of it, about five times the next factor, and the uptake count scale and the loading parameter are negligible—an ordering that the one-factor excursions of Table 2 of the main text do not reproduce, because an excursion between the endpoints of an interval is not a contribution to variance under joint sampling.

(Corollary 2), and geometry non-sufficiency at matched mean dose (Proposition 2). The three claims explicitly withdrawn—the variance maximum, mean-curve agreement, and the cleavage–effect correlation coefficient—are collected in Supplementary Table S8 and recorded here in Observation 5 and Supplementary Materials S3.1, so that they are not inadvertently reintroduced.

Supplementary Materials S6. Spatial Geometry and Experimental Design

S6.1 Geometry and the critical dispersion radius

Under the diffusion field of Equation (10) at fixed spatial mean, the silenced fraction depends strongly on the dispersion radius. At $\bar{C} = 1.5\,\bar{C}_{\mathrm{crit}} = 0.5793$ reference units, $E[\Phi]$ rises from 0.403 at $s/R = 0.30$ to 0.937 in the uniform limit (Supplementary Figure S6), and $P(Y{=}1)$ switches from 0 to 1 between $s/R = 0.40$ and $s/R = 0.45$. Mean concentration alone is therefore not sufficient to determine the outcome, establishing Equation (16).

The clinically useful summary is the critical dispersion radius. Doses are expressed as multiples of $\bar{C}_{\mathrm{crit}}$ rather than in absolute reference units, because so expressed the result is

nearly invariant between the two parameterisations of Section 4.11—at 1.5 $\bar{C}_{crit}$ and a single site, 0.441 against 0.449— whereas in absolute units it is not. At a single site, $s^*/R = 0.633$ at 1.1 $\bar{C}_{crit}$, 0.441 at 1.5×, 0.379 at 2.0×, 0.331 at 3.0× and 0.300 at 4.5×. The dose dependence is weak and strongly sublinear: quadrupling the dose from 1.1 to 4.5 times the critical concentration reduces the required dispersion radius by only 53%. Within the model, dose escalation is thus an inefficient substitute for adequate spatial distribution, which is consistent with the clinical observation that dilution volume and placement influence outcome independently of total units [53]. This is a structural correspondence and not a calibrated prediction: s^* is expressed in units of the muscle radius, and its mapping onto injection volume requires tissue-specific diffusion data the model does not contain and that detailed transport simulations are beginning to supply [36].

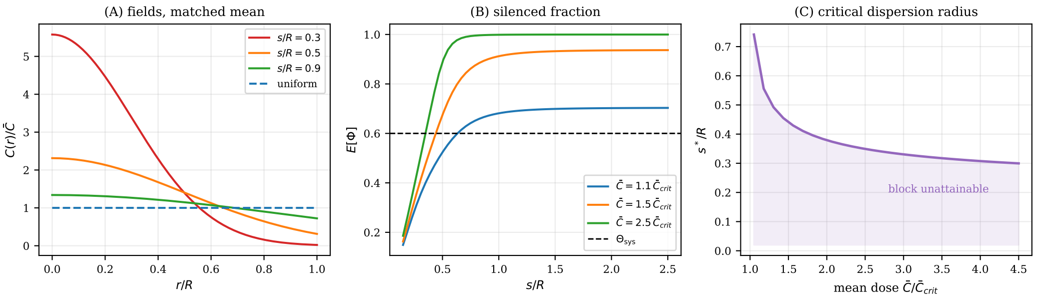

Supplementary Figure S6. Geometry at matched mean dose. (A) Concentration profiles $C(r)$ for several dispersion radii, all with identical spatial mean. **(B)** $E[\Phi]$ against s/R at three doses; the horizontal line is Θ_{sys}. **(C)** Critical dispersion radius s^* against dose, showing weak sublinear dependence.

S6.2 Fractionated deposition

Equation (10) places the whole dose at one point, whereas clinical protocols distribute it over several sites—three to five for the glabellar complex. Since Proposition 3.9 makes the spatial arrangement consequential at matched mean dose, the number and placement of sites are predicted to matter independently of the total units delivered. We therefore generalise the field to a superposition of n kernels at positions $\mathbf{x}_k$, normalised so that the spatial mean is $\bar{C}$ for every n and every s:

$$C(\mathbf{x}) = \frac{\bar{C} \sum_{k=1}^{n} e^{-\|\mathbf{x}-\mathbf{x}_k\|^2/2s^2}}{\left\langle \sum_{k=1}^{n} e^{-\|\mathbf{x}-\mathbf{x}_k\|^2/2s^2} \right\rangle_R}, \tag{S53}$$

with one site at the centre and the remainder on a ring of radius r_0. The normalisation holds the administered dose fixed, so the comparison across n is a comparison of distribution and not of amount, and $n = 1$ recovers Equation (10) identically.

Supplementary Table S24 reports the resulting critical dispersion radius. Two readings follow, and they point in opposite directions.

Whether fractionation dominates escalation depends on a quantity Supplementary Equation (S53) leaves implicit, and we resolve it rather than assume it. The field holds s fixed as n grows, which is the limit in which the dispersion radius is set by tissue diffusion and is independent of how much liquid is deposited at each site; holding the volume per site fixed, that limit multiplies the total volume by n. The opposite limit holds the total volume fixed, so each bolus is n times smaller and its radius scales as $s_n = s_1 n^{-\alpha}$ with $\alpha = 1/3$ for a spherical deposit and $1/2$ for a planar one. The two limits bracket the physics, and they disagree in sign.

At fixed s, five sites at 1.5 $\bar{C}_{crit}$ reach block under a tighter radius ($s^*/R = 0.2593$) than one site at 4.5 $\bar{C}_{crit}$ (0.2997): a 13.5% smaller requirement at one third of the toxin, rising to

36.7% at the optimal ring radius. At matched total volume the comparison reverses. The single-site equivalent radius the fractionated arm requires is $s_1 = s^* n^\alpha$, which at $r_0 = 0.5R$ gives 0.4434 under $\alpha = 1/3$ and 0.5798 under $\alpha = 1/2$, against 0.2997 for escalation—margins of −48.0% and −93.5%. Optimising the ring radius brings the first case to −8.2%, close to parity, but does not restore a positive margin under either exponent.

The reading is therefore that the lever is volume rather than site count, and fractionation helps only insofar as it raises the total volume delivered or the dispersion radius is set by diffusion rather than by the bolus. That converges from inside the model on what the transport literature reports from outside it: injection volume is the dominant determinant of spread [35,36]. We state the condition rather than select the limit that favours the architecture. Placement carries comparable weight, is robust to both limits, and behaves in a way the model was not built to produce. At $n = 5$ and $1.5\,\bar{C}_{\text{crit}}$, moving the ring from $r_0 = 0.2R$ to $0.7R$ takes s^*/R from 0.4215 to 0.1903, a reduction of 55% at unchanged dose. But the dependence is not monotone: s^*/R reaches an interior minimum of 0.1896 near $r_0 = 0.68R$ and rises again beyond it, to 0.2257 at $r_0 = 0.9R$. The optimum is a general feature rather than an artefact of one configuration, sitting between $0.60R$ and $0.72R$ across every dose and site number examined and moving inward as the dose rises (Supplementary Table S20), and its origin is geometric: sites placed too near the rim deposit part of the dose where there are fewer units to silence. Fractionated deposition therefore has a best ring radius near two-thirds of the target radius, and pushing sites further towards the periphery makes matters worse—a prediction that is directional, requires no concentration calibration, and contradicts the natural assumption that more peripheral is always better.

Two qualifications follow, and the second is the clinically useful one. Because Supplementary Table S24 is computed at the fixed convention $r_0 = 0.5R$, it understates the effect of fractionation: at the optimum, five sites at $1.5\,\bar{C}_{\text{crit}}$ beat one site at $4.5\,\bar{C}_{\text{crit}}$ by 36.7% rather than 13.5%, and nine sites at $1.5\,\bar{C}_{\text{crit}}$ beat one site at $3.0\,\bar{C}_{\text{crit}}$ by 58.1% rather than 35.1%. And the optimum is broad rather than sharp: at $n = 9$ and $2.0\,\bar{C}_{\text{crit}}$, s^*/R varies by 1.4% across an interval in r_0 of width $0.12R$, so a placement error of $\pm 0.05R$ costs under one percent. The ring radius is therefore poorly determined and need not be—which is the opposite of what an optimisation result usually implies, and is why we report it as a range rather than to three decimals. In the opposite direction the sites merge: below $r_0 \approx 0.1R$ they behave as one to within 1% of the single-site value, and below $0.2R$ to within 5% (Figure 4B).

But fractionation has a floor. At a tissue dispersion of $s/R = 0.20$ no number of sites up to 13 achieves block at $1.5\,\bar{C}_{\text{crit}}$, while 2.0× requires seven and 3.0× requires five (Figure 4C). The two levers are therefore not interchangeable in either direction, and the model does not license the general claim that more sites can substitute for dose.

The qualification of Section 4.2 applies with full force here, and units on a disc with sites on a ring are not the anatomy of any real target. What Supplementary Equation (S53) establishes is the ordering of the two levers within this architecture, not a posology.

S6.3 The design of the discriminating experiment

We verified this by simulating 20,000 subjects with independently drawn threshold populations, each unit carrying its own realisation as well as its own threshold: the interquartile range of D_i agrees with the binomial computation at $N = 200$ and at $N = 10{,}000$, under both the binary and the quantal terminal reading, to within the Monte Carlo error of the check itself (0.83% on an interquartile range at this number of subjects). The agreement is exact by construction and what is measured is the sampling noise around it (Supplementary Materials S5).

Supplementary Table S24. Critical dispersion radius s^*/R under fractionated deposition, Supplementary Equation (S53), at total dose held fixed and ring radius $r_0 = 0.5R$, at the human reference in the binary limit. Odd n places one site at the centre and $n-1$ on the ring; $n = 1$ reproduces the single-site field of Equation (10) identically. Doses are expressed as multiples of $\bar{C}_{\rm crit}$ rather than in absolute reference units: so expressed, s^*/R is nearly invariant between the human and rodent parameterisations (at $1.5\,\bar{C}_{\rm crit}$ and $n = 1$, 0.441 against 0.449), which the absolute units obscure. The ring radius $r_0 = 0.5R$ is held fixed across the table so that the comparison across n isolates the number of sites from their placement. It is a convention and not an optimum: s^*/R has an interior minimum in r_0, and at the optimum the entries for $n \geq 5$ fall by 14–64% below those tabulated here (Supplementary Table S20). The table therefore understates what fractionated deposition achieves at fixed s. It is computed in the diffusion-limited regime, in which s does not scale with the volume of the individual bolus; at matched total volume the entries for $n > 1$ must be multiplied by n^α with $\alpha \in [1/3, 1/2]$, which reverses the comparison against dose escalation (Section 4.2).

$\bar{C}/\bar{C}_{\rm crit}$	$\bar{C}$	$n = 1$	$n = 3$	$n = 5$	$n = 7$	$n = 9$
1.1	0.4248	0.6328	0.5546	0.5289	0.5138	0.5039
1.5	0.5793	0.4407	0.3390	0.2593	0.2262	0.2146
2.0	0.7724	0.3792	0.2822	0.2054	0.1766	0.1685
2.5	0.9655	0.3492	0.2566	0.1853	0.1591	0.1514
3.0	1.1586	0.3305	0.2411	0.1738	0.1491	0.1416
4.5	1.7379	0.2997	0.2162	0.1557	0.1336	0.1263

Equivalently, $1/n_{\rm eff}^2$ is linear in $1/N$ with an intercept fixed by $\sigma_{\log}$:

$$\frac{1}{n_{\rm eff}^2} = \left(\frac{W}{2\ln 3}\right)^2 \frac{1}{N} + \left(\frac{z_q\,\sigma_{\log}}{\ln 3}\right)^2. \tag{S54}$$

Supplementary Equation (S54) carries a further consequence, and it is worth stating separately because it is a bound rather than a decomposition. Since the second term does not fall with N, the first cannot drive the aggregate steepness above

$$n_{\rm eff} \leq \frac{\ln 3}{z_q\,\sigma_{\log}}, \tag{S55}$$

whatever the target size: heterogeneity alone caps the steepness a cohort curve can display, and no architecture exceeds it. At the anchored $\sigma_{\log} = 0.308$ the cap is 5.29 asymptotically and 4.74 at $N = 20$. This is testable against Table 5 in the one direction those data permit: the lower bound of 2.7 recovered from the male study lies below the cap, so the published curves are compatible with the architecture rather than merely uninformative about it, and a cohort exponent reported materially above 5 at any target size would contradict Equation (23) directly.

The design must separate the two components, not average over them. Section 3.12 previously located the test in aggregate population data and described it as a within-subject comparison. Neither is right. Aggregate parallel-group data cannot carry it, for the reason just given, and a within-subject curve does not exist, because a single subject receiving a single dose in a single target yields one outcome, not a responder-rate curve.

What the reading above makes possible is a hierarchical design. The dose–concentration gain is a property of the subject and is therefore shared across the targets treated in one session; the threshold realisation is a property of each target and is independent between them. We simulated that design directly: a mixed-effects probit [55,56] on $\log D$ with subject as a random intercept and a target-specific residual $\sigma_t = A_\sigma N_t^{-b}$, estimated by profile likelihood over b. The architecture predicts $b = 1/2$; a mean-only mapping, containing no N, implies $b = 0$.

The obvious version of the design fails. With one dose per subject per target, 400 subjects across three target classes give a standard error on b of 0.39: the two hypotheses are not separated at two standard errors, and no realistic sample size separates them. A parallel-group study cannot carry this test, and we say so plainly rather than leaving the reader to discover it.

What rescues it is a property of the model already stated above. Because unit thresholds are drawn once per muscle per subject and not resampled, $D_{s,t}$ is a fixed quantity, so repeated sessions at different doses bracket it rather than resampling it. Upper-face practice supplies exactly this structure, since patients return at three- to four-month intervals over years. With eight sessions per subject and target, 80 subjects across three target classes spanning fiftyfold in N give a standard error on b of $\simeq 0.19$ against $b = 0$; four sessions give $\simeq 0.32$. The power grid was run at 40 and at 160 subjects and not at 80, and the two behave very differently: every cell at forty subjects returned profile estimates on the boundary of the search range, so b is not estimable there and no standard error should be quoted for it, while every cell at a hundred and sixty was estimable. We therefore specify 160 subjects for the design and report the figures at 80 as an interpolation that has not been verified by simulation. These are Monte Carlo estimates from 40 replicates and carry a relative error of some 11%, so we quote them to two figures. At that standard error the design separates $b = 1/2$ from $b = 0$ at 2.7 standard deviations, not the 4.9 an earlier version of this calculation implied. Two qualifications belong with those figures. The estimator carries a finite-sample downward bias ranging from -0.06 to -0.27 according to the design, which is of the same size as the standard error, so the design must be calibrated by simulation against its own realisation before any point estimate is reported. And a Wald statement is not appropriate for a variance-scaling parameter estimated by profile likelihood at these sample sizes: the decision should rest on a profile-likelihood interval or on a simulated type I error rate at a declared level, which the figures above bound rather than replace. Simulated at a declared level, the design's power against $b = 0$ exceeds 0.75 in every estimable cell and reaches unity at the widest ladder; its power to separate $b = 1/2$ from $b = 1/3$ never exceeds 0.35, and falls to 0.03 at the fiftyfold ladder recommended above, because widening the range of N adds downward bias faster than it adds leverage. The design establishes that unit number sets the width; it does not measure the exponent, and the two should not be conflated when the study is planned. The test is therefore feasible, but it is a longitudinal titration study and not a dose-ranging trial, and nothing in the published literature has that shape.

What would invalidate the design. The estimator assumes that the target-specific residual carries only the finite-size term, $\sigma_t = A_\sigma N_t^{-b}$, and three departures put variance into that residual with no N-dependence, biasing b towards zero and therefore towards the mean-only hypothesis. First, injection geometry: if dispersion radius or site placement differs systematically between targets, the prefactor differs with them through p' (Section 4.2), so the protocol must fix volume, dilution and site count per target class rather than follow ordinary practice. Second, dispersion in N_{eff} itself: the one published facial estimate carries a test–retest coefficient of variation of 29.6% [52], and error in N_t attenuates the regression of $\log \sigma_t$ on $\log N_t$ in the standard way, so N should be measured per subject and target rather than assigned by class. Third, the endpoint: Θ_{sys} is endpoint-dependent by construction, and a graded endpoint contributes an N-independent width term through Supplementary Equation (S32), so a wrinkle scale in one target and a movement amplitude in another compare different collective thresholds on differently smoothed readouts and the collapse is not expected to hold. The design is thus demanding in ways a dose-ranging trial is not, and we state the requirements rather than presenting feasibility alone.

S6.4 What the collapse test requires of the targets compared

The test is a parametric restriction and can be stated as one: across J targets the architecture spends a single parameter, the constant W, where a mean-only mapping spends J, one Hill exponent per target. The collapse is therefore a hypothesis with $J-1$ degrees of freedom, testable by the usual comparison of nested fits, and what is at stake is not whether a mean-only mapping can describe each target but whether it must be re-fitted for each. Three consequences follow without further assumption. *(a)* A Hill curve calibrated on one target mispredicts the transition width on a target of different N by the factor $\sqrt{N/N_0}-1$ derived in Supplementary Materials S2, so a single fitted Hill exponent cannot serve two targets of different size. *(b)* At fixed N, targets with broader terminal threshold dispersion show proportionally wider transitions, which jointly with the collapse identifies σ_θ. *(c)* The collapse isolates N only if the prefactor is common across the targets compared. What matters is therefore not how uncertain each parameter is—which is what Table 2 reports—but how much it differs *between* the targets, since a parameter with a wide range that is common to both contaminates neither. Two qualify. Quantal content differs with fibre type and enters through (19), moving W by 10.8% across the measured range $\bar{m}_0 = 20$–30; the number of endplates per fibre differs with muscle and moves it by 10.0%. Orbicularis and corrugator differ markedly in fibre composition, so the comparison should match targets on fibre type or estimate quantal content independently. The remaining factors—the uptake stage, the per-event yield, the collective threshold—are properties of the toxin, the preparation and the endpoint, and are common across targets treated in one session provided the protocol holds them so. Population size and per-junction reliability are thereby separable sources of sharpness, which a mapping in $\bar{C}$ alone cannot distinguish.

The design that carries the test is itself a contribution rather than a caveat. Section 4.8 specifies it: a longitudinal titration across targets of differing independent-unit count, with the subject-level gain absorbed as a random effect, motor-unit number estimated per subject and target, and volume, dilution, site count and endpoint held fixed across the comparison. To our knowledge no such protocol has been specified for a pharmacodynamic architecture in human muscle, and the requirements are stated with the power calculation and the contaminants ordered rather than left to be discovered.

S6.5 The three empirical tests, and what $P(Y{=}1 \mid \bar{C})$ is

(i) Sharpness collapse across targets. Responder endpoints collected in targets of differing size yield relative transition widths whose product with $\sqrt{N}$ should be constant (Corollary 3.7). The design is hierarchical rather than within-subject in the literal sense—a single subject yields one outcome per target, not a curve, and it is the subject-level random effect that removes the shared gain—and a parallel-group comparison cannot detect the effect at any sample size (Section 4.8). No single-terminal measurement is needed; N is estimated by motor-unit number estimation (Section 6).

(ii) Non-transportability. A Hill curve calibrated on one target should mispredict transition width on another, in the direction and by the magnitude (S35) specifies—by half at a fourfold smaller target and by a factor of two at a fivefold larger one (Figure 2). A finding that a single calibrated mapping transports across targets of different size would falsify the architecture directly.

(iii) Dilution-volume dependence of outcome variance. Prediction 2 implies that more focal deposition at equal total dose raises the probability of sub-threshold outcome. Retrospective series with paired dose and dilution records could be interrogated for this pattern, but only if the doses sit near the transition: at the reference parameters the contrast between dispersion radii is largest at about 1.2 $\bar{C}_{\text{crit}}$ and is exactly zero at twice that dose and above, where every arm saturates. A null result obtained at responder rates of 95–100%

therefore carries no information at any sample size. Geometry-independent outcomes at a dose near the transition would contradict Proposition 2.

None of these is decisive on its own, and each admits alternative explanations. Their value is that they are testable now.

What $P(Y{=}1 \mid \bar{C})$ is. One clarification is needed before the decomposition, because it determines what kind of experiment can measure the width. Unit thresholds are properties of a given muscle in a given subject: they are drawn once, not resampled at each observation. Under that reading $\Phi_i(\bar{C})$ is a deterministic increasing step function for subject i, and the subject has a sharp critical dose $D_i = \inf\{\bar{C} : \Phi_i(\bar{C}) \geq \Theta_{\text{sys}}\}$ with no within-subject randomness at all.

The arithmetic is unchanged: $D_i \leq \bar{C}$ if and only if $\Phi_i(\bar{C}) \geq \Theta_{\text{sys}}$, and $N\Phi_i(\bar{C})$ is binomial, so $P(Y{=}1 \mid \bar{C})$ as computed throughout this paper is exactly the distribution function of D_i across threshold realisations. The interpretation, however, changes and matters. $P(Y{=}1 \mid \bar{C})$ is not a within-subject response probability; it is the between-subject distribution of the individual critical dose. The finite-size term of (23) is therefore itself a component of between-subject variance rather than a separate channel, and the question the decomposition answers is whether it can be separated from the others.

The model separates two pairs of manoeuvres that clinical usage conflates. Raising the number of sites at fixed volume per site and raising it at fixed total volume have opposite consequences (Section 4.2), so what the architecture identifies as the lever is volume rather than site count. And dilution acts through two channels that are one manoeuvre at the syringe: insofar as it changes the concentration delivered at fixed spatial arrangement it is a dose gain and leaves the falsifiable content untouched, while insofar as it changes the dispersion radius it changes the shape of the field and does not (Corollary 3.7). A clinician varying dilution is varying both at once, and the model says which of the two the outcome follows. This is structurally consistent with the observation that dilution volume and injection placement affect outcome independently of dose [53], with the qualification stated in Supplementary Materials S6.1.

S6.6 Endpoint dependence, the coefficient that does not discriminate, and the published exponents

One objection is best met here rather than deferred, because it is the sharpest one available. The collective threshold is endpoint-dependent by construction: a wrinkle scale, a movement amplitude and a force measurement place their detection thresholds at different silenced fractions, so Θ_{sys} is not a single number given by the tissue. It does not follow that the distinction drawn here is a statement about measurement rather than about mechanism. The endpoint fixes the *value* of the requirement; it does not determine whether that requirement scales with the population. Under a fractional threshold $\bar{C}_{\text{crit}}$ solves $p(\bar{C}) = \Theta_{\text{sys}}$ at every N, whatever value the endpoint assigns to Θ_{sys}, so potency is invariant; under a fixed reserve the required fraction $(N - c + 1)/N$ rises with N whatever value the endpoint assigns to c. The discriminator is a comparison of scaling and not of level, and it survives the choice of endpoint. What does not survive it is the mixing of endpoints across the targets being compared, which is a constraint on the experiment—stated as such in Section 6—and not on the claim.

One quantity does *not* discriminate, and we say so because it is the natural candidate. The coefficient $2 \ln 3/W = 2.382$ of (15) is matched by multi-hit target theory, which returns $2 \ln 3/(2z_{0.75}) = 1.629$—inside the interval $[1.61, 2.84]$ that the joint parameter uncertainty of Table 2 admits for our own value. Nor does its constancy across targets separate them, since the multi-hit coefficient is constant too. Measuring $n_{\text{eff}}/\sqrt{N} \approx 2.4$ is therefore consistent with either architecture, and the coefficient should not be read as a signature of this one.

The observed exponents are at least 1.5–2.7, and their upper end is not identified. The reason is visible in Table 5: in all three studies the responder rate at the lowest dose tested already exceeds one half, so the lowest arm lies above the median effective dose and the transition was never sampled. Above that point the studies saturate, three of the four male arms reporting complete response. The exponents of 2.8 and 4.4 quoted in earlier versions of this table are recovered exactly by replacing those ceiling arms with a continuity-corrected 19.5/20; they are therefore logarithms of a tie-breaking convention rather than measurements, and substituting 0.999 for 0.975 turns 4.4 into 9.1 without altering a single datum. Refitting all four arms by binomial likelihood returns 1.49 (95% CI $[0.49, 2.53]$) for abobotulinumtoxinA and a lower bound of 2.7 with no upper limit for the male study. Published glabellar dose-ranging data therefore cannot estimate the transition width under any architecture. What they can do is bound it, and the bound is the subject of the next section.

Two features of the anchor should be stated, and they pull in opposite directions. It comes from cervical dystonia rather than from a facial target, so transporting it across muscle, indication and population is an assumption, which is why the sensitivity of every quantity below to $\sigma_{\log}$ is reported rather than asserted. And the trial population was selected for prior tolerance and response, which truncates the requirement distribution and if anything understates $\sigma_{\log}$: that makes the finite-size share quoted next an upper bound rather than a conservative one, while making the ceiling of (S55) less binding than the true value would.

One independent signal bears on that transport and points the other way, and we record it because it was not sought. Propagating $\sigma_{\log} = 0.308$ onto the duration axis through the elasticity of Section 4.10 accounts for a dispersion of 0.347 in $\log T$ on its own. The within-arm dispersion of time to relapse recoverable from the published male series runs from 0.270 to 0.585 across the four arms, so at the narrower arms the observed dispersion sits *below* what the anchor alone would produce. Two readings are open and we do not choose between them: either the anchor does not transport from cervical dystonia to the glabella, or those arms operate well above twice the critical dose, where the elasticity has fallen and the propagation is smaller than the operating point implies. Either way, $\sigma_{\log} = 0.308$ should be read as an anchor with a direction of doubt attached and not as a measurement.

S6.7 What the sharpness law forbids, and secondary non-response

Its value is that it is a constraint, not a capability. The sharpness law forbids rather than permits: once a mapping is calibrated on one injection target, the architecture fixes the transition on a target of different unit number with no remaining freedom. A mean-only mapping contains no N and makes no such commitment. That asymmetry is what makes the comparison meaningful, and it is why we rest the paper on this result rather than on the variance maximum or the reproduction of a mean curve—neither of which discriminates against a stochastic mean-only benchmark (Observations 1 and 2).

Two parameters of that simulation are design choices rather than model quantities, and we state them because the figures below cannot be reproduced without them. Subjects differ in their dose requirement with a between-subject dispersion of $\sigma_{\log} = 0.55$ on $\log D$, and the doses of the repeated sessions are drawn uniformly over ± 1.2 in $\log D$ about the population median requirement. The dispersion is set above the value anchored in Section 4.8, $\sigma_{\log} = 0.308$, and is conservative where it matters: repeating the simulation at the anchored value leaves the standard error on b essentially unchanged and reduces the finite-sample downward bias from -0.29 to -0.06, so the design separates $b = 1/2$ from

$b = 0$ more sharply there, not less. Neither reading separates $b = 1/2$ from $b = 1/3$, which is the conclusion this section reaches at both values.

We deliberately do not claim that the framework explains secondary non-response. The drift extension shows that a monotone upward threshold shift produces finite-time escape from the therapeutic window, but the drift rate is unmeasured, the functional form is chosen for parsimony, and the compensatory mechanisms invoked—sprouting and reinnervation [62], SNAP-23 recruitment [63,64], compensatory facilitation—are not established as cumulative across sessions. Indeed, sprouting after BoNT-A poisoning follows a biphasic course that largely resolves within a treatment cycle [62], which argues against a simple cumulative reading. The extension is a formal demonstration that a non-immunological route to attenuation exists within this model class, not evidence that it operates clinically.

Supplementary Materials S7. Duration Under a Minimal Recovery Law

Section 2.10 states that the model as constructed has no time axis. This appendix adds the smallest extension that supplies one, derives what the architecture then implies about the duration of effect, and states which of those implications survive and which do not. Two of them do not, and both are reported here in full.

S7.1 The extension, and the reduction that makes it exact

Let the cleaved fraction recover exponentially from its value at peak,

$$f_i(t) \;=\; f_{\max,i}\, e^{-t/\tau}, \tag{S56}$$

with τ common across units, and let a unit remain silenced while $f_i(t) \geq \theta_i$. Define the duration of effect as

$$T \;:=\; \inf\{\, t > 0 \;:\; \Phi(t) < \Theta_{\mathrm{sys}} \,\}. \tag{S57}$$

The condition $f_{\max,i} e^{-t/\tau} \geq \theta_i$ is equivalent to $f_{\max,i} \geq \theta_i\, s$ with $s := e^{t/\tau}$. Time therefore enters *only* as a multiplicative rescaling of the threshold law, so no new response function is required: the unit silencing probability at time t is $p(\bar{C}, s)$, the function already used throughout with $\theta_j \mapsto \theta_j / s$. Conditional on the field the unit states remain independent Bernoulli, $N\Phi(t)$ remains binomial, and the quantiles of T are obtained by exact inversion of the binomial tail in s rather than by simulation, with the quantile index reversed because p decreases in t. All values reported below are exact inversions; no Monte Carlo enters any tabulated quantity. At $t = 0$ the extension reproduces the static model bitwise, which is the first of the gates the computation enforces.

S7.2 The duration invariant, and why its exactness is dimensional

Proposition 1 applies verbatim with s in place of $\bar{C}$, and the scale-free product on the time axis,

$$W_T \;:=\; \frac{\Delta T_q}{T_{50}}\,\sqrt{N}, \tag{S58}$$

converges to 1.4401 at the human reference under the quantal response (Supplementary Table S25). The residual is $O(N^{-1})$ and not $O(N^{-1/2})$: the relative error multiplied by N stays within $[1.516, 1.546]$ across $N = 20$–10^4, an excursion of 2%, while the same residual multiplied by $\sqrt{N}$ spans $[0.015, 0.339]$, an excursion of 229%. This is the same order the closed form attains on the dose axis (Supplementary Table S10), and it is the sense in which the architecture is inherited rather than merely analogous.

The recovery timescale τ cancels identically—across $\tau = 30$–180 days the product varies by 3.7×10^{-15}, which is 31 units in the last place. We are explicit that this is a

dimensional identity and not a result: since $T_q = \tau \ln s_q$ with s_q independent of τ, the ratio in (S58) is scale-free by construction, and Supplementary Materials S7.4 confirms that the same exactness holds for a linear and for a sigmoidal recovery law. What the cancellation does establish is that the falsifiable content on the time axis, like that on the dose axis, does not require the scale to be measured. Supplementary Figure S7 shows both the scale relation and the collapse.

S7.3 The closed form, and where it breaks

Carrying the delta method through gives, in the mean-field reduction,

$$W_T \;=\; \frac{2z_q\sqrt{\Theta_{\rm sys}(1-\Theta_{\rm sys})}}{f_\theta(f^*)\,f^*\,\ln(f_{\max}/f^*)}, \tag{S59}$$

with $f^* = F_\theta^{-1}(\Theta_{\rm sys})$. Against the mean-field computation (S59) behaves as its analogue does on the dose axis: the relative error times N is constant to 2.1% under the quantal reading and 3.3% under the binary one. Against the *full* model it does not, missing by -4.76% and -12.26% respectively at $N = 10^4$, and the discrepancy does not vanish in N because it is the mean-field error itself: (S59) cancels the Poisson uptake that the full model retains. Substituting the exact slope $|p'(s^*)|\,s^*\ln s^*$ for $f_\theta(f^*)f^*\ln(f_{\max}/f^*)$ restores $O(N^{-1})$ agreement with the full model, giving the limit 1.44028 against the computed 1.44005. We report both rather than adjusting the first, because the comparison isolates which ingredient fails and shows that the structure does not.

S7.4 Sensitivity to the recovery law: what is withheld

The exactness of the τ cancellation invites the conclusion that the constant is a property of the architecture. It is not. Replacing (S56) by a linear law $f(t) = f_{\max}(1 - t/T_{\rm rec})$ and by a sigmoidal law, both at matched first moment, gives

$$W_T \;=\; 1.4401 \quad (\text{exponential}), \qquad 1.1779 \quad (\text{linear}), \qquad 0.8073 \quad (\text{sigmoidal}),$$

an excursion of 44%; the τ cancellation holds to the same 3.7×10^{-15} under all three, confirming that it is dimensional. Because W_T is itself scale-free, that excursion cannot be removed by any choice of moment matching.

The contrast with the dose axis is the point, and it is not a matter of degree. There, matching the first two moments across the Beta, log-normal and gamma families moves W by 0.25% (Supplementary Materials S1.15), because Poisson uptake convolves the threshold law before the collective integration; no such convolution acts on the time axis, where the recovery law enters after it. The recovery law in human facial muscle is not measured. We therefore report the structure of (S58) and withhold its value, and Table 1 carries a status for that which is distinct from the conditional quantities of Table 2: no substitution of a measured parameter recovers a withheld quantity, because what is unmeasured is a functional form and not a number.

Heterogeneity of τ *between units* within one target, by contrast, is benign: at coefficients of variation of 0.1, 0.2 and 0.3 the invariance survives intact and the value moves by $+0.65\%$, $+2.75\%$ and $+6.48\%$, an order of magnitude below the sensitivity to the form.

S7.5 Duration in a cohort: the decomposition transports in two terms, not three

Equation (23) separates the observed relative width on the dose axis into a finite-size term and a between-subject term. On the time axis the same separation holds,

$$w_{\rm obs}^2 \;=\; w_{\rm fs}(N)^2 \;+\; w_{\rm bs}(\sigma_\tau, \sigma_{\log})^2, \tag{S60}$$

reproducing exact computation to within 3.20% over 105 cells, with the residual vanishing in N.

What does *not* hold is the natural further resolution of the between-subject term into the dispersion of the recovery timescale and the propagated dispersion of the dose gain, added in quadrature. That form misses by -6.9% to $+5.5\%$, and the reason is identifiable: the gain does not propagate log-normally onto $\ln T$, its local elasticity $\mathrm{d}\ln T_{50}/\mathrm{d}\ln D$ running from 1.73 to 0.71 across the quartiles of the gain itself. The elasticity at the operating point is 1.0786, recovered numerically and not assumed; the chord between the tabulated extremes of Supplementary Table S26 is 1.2072 and is not the same quantity. We report (S60), which holds, rather than adjusting the coefficients of the three-term form, which does not.

S7.6 How much of the observed variance the finite-size term carries

Supplementary Table S27 evaluates the finite-size share on the time axis against the share the manuscript reports on the dose axis at the same anchored $\sigma_{\log} = 0.308$. At $\sigma_\tau = 0.30$ the two axes very nearly coincide: the term exceeds a tenth of the variance below $N = 44$ and a quarter below $N = 14$, against $N \approx 44$ and $N \approx 15$ on the dose axis. The coincidence is not structural—it is the value of σ_τ at which the two happen to cross—and at $\sigma_\tau = 0.10$ the time axis carries 1.76 times the dose-axis share while at 0.50 it carries 0.56 times. Supplementary Figure S8 plots both the share and the ratio between the two axes.

Calibrating σ_τ, and why it cannot be done cleanly. No measurement of the between-subject dispersion of the recovery timescale exists, and we invert it from published relapse data as the manuscript inverts $\sigma_{\log}$ from a dose interquartile range. Two routes are available. The first combines the elasticity implied by the male dose-ranging series, $\ln(24.2/17.6)/\ln(80/20) = 0.230$, with the relapse fractions of the female series, and returns a total dispersion of $\ln T$ between 0.104 and 0.228; it presupposes that an elasticity measured over one dose range transfers to another, which the extension itself says it does not, since the elasticity falls with dose. The second uses the within-arm dispersion of time to relapse in the male series alone, two time points within the same group and no dose model, and returns 0.270 to 0.585 across the four arms with a mean of 0.400. We adopt the second as the working value and the union $[0.104, 0.585]$ as the interval.

That dispersion is not σ_τ. The anchored gain already propagates 0.347 onto $\ln T$ at the operating point, and the finite-size term at $N = 75$ contributes 0.121; subtracting both leaves $\sigma_\tau \approx 0.158$, and at the extreme arms the residual runs from negative to 0.456. The attribution that does not subtract—charging the whole observed dispersion to σ_τ while leaving the gain term standing—double-counts, inflates the between-subject total and therefore *lowers* the finite-size share; it is the conservative choice and we report it as the primary one. The share depends only on the between-subject total and not on how it is split.

The negative finding. We undertook this calculation expecting the duration endpoint to be the more accessible of the two, since time to relapse is recorded in every registration trial while the dose–response transition is not (Section 4.8). It is not. At $N_{\mathrm{eff}} = 75$ the finite-size share on the time axis lies between 2.71% under the conservative attribution and 9.71% under the favourable one, against 6.17% on the dose axis, and the ratio between the axes spans 0.22 to 1.92 once the three recovery laws are included—the break-even σ_τ itself moving from 0.311 to 0.251 to 0.171 across them. The share is in fact *more* sensitive to the recovery law than the invariant is, by 51% against 44%, because at small shares it scales as W_T^2.

What can be said is bounded, and we say only that. The finite-size term does not vanish on this axis either: under the most conservative attribution it remains above 5% of the observed variance up to $N = 39$ and above 10% up to $N = 17$, and under the most

favourable up to $N = 157$ and $N = 74$. Duration is therefore measurable in a cohort in small targets and not in large ones, which is the conclusion the dose axis reaches, by the same mechanism, and to no better resolution. A study designed to interrogate the architecture through duration would not be easier than one designed to interrogate it through dose, and we record that as a result rather than leaving it to be rediscovered.

One incidental figure. Between 1.2% and 1.7% of simulated subjects have $T = 0$: the collective threshold is not met even at peak, so they contribute no duration at all and are excluded from the quantiles rather than entered as zeros. At a dose of exactly $\bar{C}_{\mathrm{crit}}$ the lower quartile of duration does not exist for the same reason, and that cell is recorded as a failure rather than filled (Supplementary Table S26). It is what a median effective dose means, read on the time axis.

Supplementary Table S25. The duration invariant against unit number, at the human reference under the quantal response, by exact binomial inversion in $s = e^{t/\tau}$. The product converges to 1.44005 against the closed form with exact slope, 1.44028. The last column is the relative residual multiplied by N: constant to 2% across the range, which establishes the order as $O(N^{-1})$; the same residual multiplied by $\sqrt{N}$ varies by 229%. Values are independent of τ to 31 units in the last place.

N	$\Delta T_{0.75}/T_{50}$	$(\Delta T_{0.75}/T_{50})\sqrt{N}$	residual $\times N$
20	0.29937	1.33881	1.5159
50	0.19762	1.39741	1.5300
75	0.16297	1.41134	1.5364
100	0.14184	1.41843	1.5402
200	0.10106	1.42925	1.5442
500	0.06421	1.43584	1.5455
1,000	0.04548	1.43806	1.5459
10,000	0.01440	1.44005	1.5459

Supplementary Table S26. Median duration and relative width against dose, at $N = 10^4$ and $\tau = 90$ days, under exponential recovery. Duration rises by a factor of 4.93 across the range with a local elasticity of 1.0786 at the operating point ($2\,\bar{C}_{\mathrm{crit}}$). The absolute interquartile width is nearly flat, so the contraction of the relative width is the median growing rather than the dispersion shrinking. At $\bar{C}_{\mathrm{crit}}$ exactly the lower quartile does not exist, because the collective threshold is not met at peak in half the population; the cell is recorded as a failure and left empty.

$D/\bar{C}_{\mathrm{crit}}$	T_{50} (days)	$\Delta T_{0.75}$ (days)	$\Delta T_{0.75}/T_{50}$	status
1.0	—	—	—	failed at $q = 0.25$
1.2	10.827	0.5486	0.050664	ok
1.5	22.571	0.5249	0.023254	ok
2.0	35.037	0.5045	0.014401	ok
2.5	42.505	0.4953	0.011652	ok
3.0	47.174	0.4908	0.010405	ok
4.5	53.396	0.4871	0.009122	ok

Supplementary Table S27. Percentage of the observed variance carried by the finite-size term, on the time axis against the dose axis, at the anchored $\sigma_{\log} = 0.308$ and under exponential recovery. The dose column is the quantity plotted in Figure 5. At $\sigma_\tau = 0.30$ the two axes nearly coincide; the crossing is a value of σ_τ and not a structural property, and it moves to 0.251 and 0.171 under linear and sigmoidal recovery.

	duration, by σ_τ			dose
N	0.10	0.30	0.50	(23)
20	29.18	18.73	10.74	19.78
50	15.22	9.13	4.98	8.98
75	10.88	6.39	3.44	6.17
100	8.47	4.92	2.63	4.70
200	4.49	2.56	1.35	2.41
500	1.86	1.05	0.55	0.98
1,000	0.94	0.53	0.28	0.49

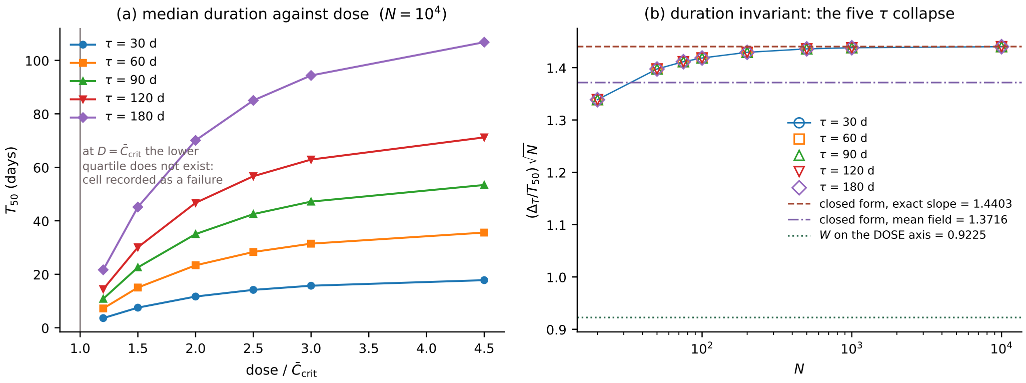

Supplementary Figure S7. Duration under exponential recovery. (A) Median duration against dose, for five recovery timescales. The curves are related by a pure scale, which is the cancellation of Supplementary Materials S7.2 seen directly; the vertical line marks $\bar{C}_{\mathrm{crit}}$, at which the lower quartile ceases to exist. **(B)** The scale-free product against unit number: the five timescales collapse onto one another to 31 units in the last place, and the product converges to the closed form evaluated at the exact slope. The mean-field closed form and the dose-axis constant W are drawn for comparison; that the second is a different number is expected, since the two are widths of different quantities.

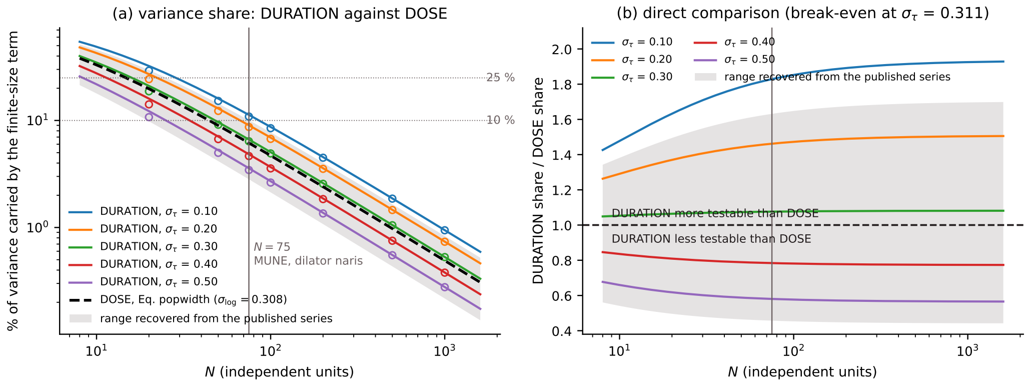

Supplementary Figure S8. Duration in a cohort. (A) Percentage of observed variance carried by the finite-size term against unit number, for five values of the between-subject dispersion of the recovery timescale, with the dose-axis curve of Figure 5 drawn as a dashed line for comparison and the shaded band spanning the dispersion recoverable from the published series. Circles are exact computations; lines are (S60). **(B)** The ratio between the two axes. The duration endpoint is the more accessible one only below a break-even σ_τ of 0.311, and that break-even moves to 0.251 and 0.171 under the other two recovery laws, so the sign of the comparison is not determined by the data.
